# ACAD8 deficiency promotes pathological cardiac hypertrophy in response to pressure overload by regulating histone isobutyrylation

**DOI:** 10.1038/s41467-026-72949-w

**Published:** 2026-05-11

**Authors:** Jing-Yi Wang, Xin-Yan Zhao, Xin Sun, Yu-Fei Zhang, Li-Hong Sun, Xiang Wei, Ding-Sheng Jiang, Hui-Yu Wang, He-Ping Wang, Ke-Xin Si, Xiaoqiang Tang, Hou-Zao Chen, De-Pei Liu

**Affiliations:** 1 State Key Laboratory of Common Mechanism Research for Major Diseases, Department of Biochemistry & Molecular Biology, Institute of Basic Medical Sciences & School of Basic Medicine, Chinese Academy of Medical Sciences & Peking Union Medical College, Beijing, China; 2 Jiangxi Provincial People’s Hospital, The First Affiliated Hospital of Nanchang Medical College, Jiangxi, China; 3 Center for Experimental Animal Research, Institute of Basic Medical Sciences, Chinese Academy of Medical Sciences and Peking Union Medical College, Beijing, China; 4 Division of Cardiovascular Surgery, Tongji Hospital, Tongji Medical College Huazhong University of Science and Technology, Wuhan, China; 5 Key Laboratory of Birth Defects and Related Diseases of Women and Children of MOE, West China Second University Hospital, Sichuan University, Chengdu, China; 6 Institute of Aging and Health, Chinese Academy of Medical Sciences, Beijing, China

**Keywords:** Cardiac hypertrophy, Heart failure

## Abstract

Branched-chain amino acids play critical roles in cardiac physiology and diseases. Genetic deficiency in the valine catabolic enzyme ACAD8 is clinically associated with isobutyryl-CoA deregulation and cardiomyopathy in humans, but its roles in cardiac disease remain undefined. Here, we show that the levels of ACAD8 are reduced in humans and male mice with cardiac hypertrophy. Cardiomyocyte-specific *Acad8* knockout in male mice exacerbates cardiac hypertrophy and cardiac dysfunction underwent pressure overload. Mechanistically, *Acad8* deficiency leads to the accumulation of its substrate isobutyryl-CoA, which enhances histone isobutyrylation, chromatin accessibility and TEAD2 enrichment at promoter regions of hypertrophy-related genes, which increases the sensitivity to hypertrophic stress in mouse hearts and cardiomyocytes. Conversely, ACAD8 overexpression in cardiomyocytes suppresses isobutyryl-CoA accumulation and histone isobutyrylation, thereby attenuating cardiac hypertrophy and dysfunction. These results elucidate the roles of ACAD8 deficiency in cardiac diseases and reveal histone isobutyrylation in transcription regulation and cardiac pathology.

## Abstract (web summary)

Valine metabolism disruption is linked to heart disease, but the mechanisms are unclear. Here, the authors show that ACAD8 deficiency drives isobutyryl-CoA accumulation and histone isobutyrylation, increasing chromatin accessibility and gene expression to promote cardiac hypertrophy, while ACAD8 overexpression reverses these effects.

## Introduction

Heart failure, which is a global health concern, remains a leading cause of mortality and morbidity^1,2^. The heart is a high-energy-consuming organ that utilises a variety of substrates, including fatty acids, glucose, ketone bodies, amino acids, and lactate, for energy metabolism. Pathological cardiac hypertrophy due to abnormal energy metabolism is an important feature of heart failure^3–5^.

Most studies on cardiovascular metabolism have focused on the use of glucose and fatty acids as fuels^6,7^. However, some currently evidences suggest that changes in valine metabolism are associated with cardiovascular disease^8–10^. Notably, valine significantly accumulates in both cardiac tissue and plasma of heart failure patients, suggesting that altered valine metabolism may contribute to the development of pathological cardiac hypertrophy^11,12^. Acyl-CoA dehydrogenase family member 8 (ACAD8) specifically mediates the α, β-dehydrogenation of isobutyryl-CoA in the valine degradation pathway^13^. Clinically, the dysregulation of isobutyryl-CoA levels due to isobutyryl-CoA dehydrogenase deficiency (IBDD) caused by ACAD8 mutations is associated with multiple disorders, including cardiomyopathy, hypotonia, and developmental delay^14–16^. Intracellular short-chain acyl-CoAs can form post-translational modifications (PTMs) on histone lysine residues, altering chromatin structure and gene transcription, which plays a significant role in cardiac diseases^17–20^. However, the correlation between ACAD8 deficiency, isobutyryl-CoA dysregulation and pathological cardiac hypertrophy, as well as the underlying mechanism, remains unknown.

In this study, we demonstrated that the expression of ACAD8 was decreased in the hearts of patients with hypertrophic cardiomyopathy and in the hearts of mice with pathologic cardiac hypertrophy. Using a mouse model of cardiac hypertrophy, we found that cardiomyocyte-specific *Acad8* knockout promoted pathological cardiac hypertrophy and cardiac dysfunction. Our findings demonstrated that the accumulation of isobutyryl-CoA and the concomitant alterations in histone isobutyrylation induced by ACAD8 deficiency promoted the expression of hypertrophy-associated genes. Furthermore, AAV9-mediated cardiomyocyte-specific ACAD8 overexpression (ACAD8-OE) reduced isobutyryl-CoA levels and attenuated the expression of hypertrophy-associated genes, ultimately rescuing cardiac hypertrophy and cardiac dysfunction.

## Results

### Valine catabolism is impaired in pathological cardiac hypertrophy

**Fig. 1:**
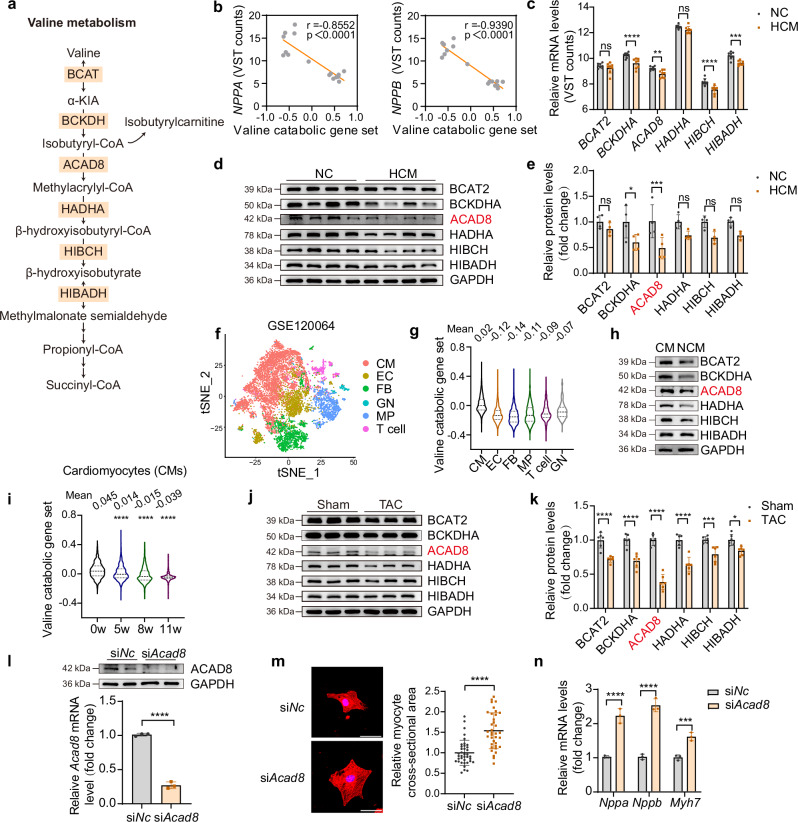
Valine catabolism pathway is deregulated in pathological cardiac hypertrophy. **a** Valine metabolic pathway. BCAT, branched-chain aminotransferase; BCKDH, branched-chain α-keto acid dehydrogenase; ACAD8, isobutyryl-CoA dehydrogenase; HADHA, hydroxyacyl-CoA dehydrogenase trifunctional multienzyme complex subunit α; HIBCH, 3-hydroxyisobutyryl-CoA hydrolase; HIBADH, 3-hydroxyisobutyrate dehydrogenase. **b** Correlation between valine catabolism gene set scores and NPPA/NPPB expression in human hearts (GSE133054; *n* = 16). **c** Expression of valine catabolic genes in non-failing control (NC; *n* = 8) and hypertrophic cardiomyopathy (HCM; *n* = 8) patients (GSE133054). From left to right: *P* >  0.9999, ****P < 0.0001, **P = 0.0078, *P *= 0.3399, ****P = < 0.0001, ***P  =  0.0001, ns, not significant, by two-way ANOVA with Bonferroni post hoc correction. **d**,**e** Western blotting of valine catabolic proteins. From left to right: *P* = > 0.9999, *P  = 0.0163, ***P = 0.0009, P  =  0.2337, P  =  0.1044, P  =  0.2144, respectively, ns, not significant, by two-way ANOVA with Bonferroni post hoc correction. *n*  =  4 mice/group. **f**,**g** tSNE plot of cardiac cell types (**f**) and valine catabolism gene set scores (**g**) across cardiomyocytes (CMs), endothelial cells (ECs), fibroblasts (FBs), macrophages (MPs), T cells and granulocytes (GNs) in mouse hearts (GSE120064). From left to right: *n*  =  5656, 1858, 1775, 1413, 606, 181 cells. **h** Western blotting of valine catabolic proteins in CMs and non-cardiomyocytes (NCMs). **i** Valine catabolism gene set scores in CMs at different stages of cardiac hypertrophy (GSE120064). ****P  <  0.0001, compared with 0 week, by one-way ANOVA with Bonferroni post hoc correction. From left to right: *n*  =  934, 2016, 1775, 1143, 398 cells. **j**,**k** Western blotting of valine catabolic proteins in mice 4 weeks after Sham or TAC surgery. ****P  <  0.0001, ***P  =  0.0006, *P  =  0.0153, by two-way ANOVA with Bonferroni post hoc correction. *n*  =  6 mice/group. **l** Acad8 knockdown efficiency in NRCMs assessed by Western blotting and qRT-PCR. ****P <  0.0001, by two-tailed unpaired Student’s t-test. *n*  =  3 biologically independent samples. **m** Cell size in siNc (*n* = 38) and siAcad8 (*n* = 36) NRCMs. Scale bar: 30 μm. ****P <  0.0001, by two-tailed Mann-Whitney test. **n** Expression of hypertrophic genes in NRCMs. ****P  <  0.0001, ***P  =  0.0004, by two-way ANOVA with Bonferroni post hoc correction. *n*  =  3 biologically independent samples. In violin plots (**g**,**i**) centerline: median; box: 25–75th percentiles; bounds: min/max; curvature: data density. Data are presented as mean values  ±  SD. GAPDH served as a loading control for western blots in **d**,**h**,**j**. Source data are provided as a Source Data file.

Valine catabolism occurs within cellular mitochondria and involves several different types of enzymes^21^ (Fig. 1a). To investigate the relationship between valine catabolism and cardiac hypertrophy, we analysed the correlation between the valine catabolism gene set score and the atrial natriuretic peptide (*NPPA*) and brain natriuretic peptide (*NPPB*) expression levels using the GSE133054 dataset. Pearson correlation analysis revealed a significant negative correlation between the valine catabolism gene set score and the *NPPA* and *NPPB* mRNA levels in the hearts of control and hypertrophic cardiomyopathy patients (Fig. 1b). Furthermore, analysis of data from the GSE133054 dataset revealed that the mRNA expression of several valine catabolism-associated genes was decreased in the hearts of patients with hypertrophic cardiomyopathy (Fig. 1c). Moreover, a decreasing trend in the protein levels of genes that are involved in valine catabolism was validated in the hearts of patients with hypertrophic cardiomyopathy (Fig. 1d, e and Supplementary Table 1). To elucidate the role of valine catabolism in the progression of cardiac hypertrophy in mice, we conducted single-cell RNA-seq analysis of the hearts of mice that were subjected to transverse aortic constriction (TAC) using the GSE120064 dataset. We observed that the valine catabolism-associated gene set was most highly expressed in cardiomyocytes (Fig. 1f, g). The protein level was validated in cardiomyocytes and non-cardiomyocytes isolated from mouse hearts (Fig. 1h). Further analyses revealed that the cardiomyocyte valine catabolism gene set score was consistently decreased at 5, 8, and 11 weeks (Fig. 1i). Similarly, we observed reduced protein levels of key valine catabolism enzymes in hypertrophic mouse hearts (Fig. 1j, k).

In particular, we noted the most significant decrease in the protein level of ACAD8 in both the hearts of patients with hypertrophic cardiomyopathy and the hearts of mice with cardiac hypertrophy (Fig. 1d–e, j–k). This finding suggested that ACAD8 may play a pivotal role in pathological cardiac hypertrophy. To explore the direct effect of ACAD8 deficiency on cardiomyocyte hypertrophy, we used *Acad8*-targeted siRNA to knock down *Acad8* expression in neonatal rat cardiomyocytes (NRCMs). The results indicated that *Acad8* knockdown directly induced cardiomyocyte hypertrophy, as evidenced by increase in cardiomyocyte size and the expression of hypertrophy-associated genes (Fig. 1l–n). These findings suggest that impaired valine catabolism, particularly ACAD8 deficiency, may play a crucial role in cardiac hypertrophy.

### Cardiomyocyte-specific *Acad8* knockout exacerbates cardiac hypertrophy in response to pressure overload in mice

**Fig. 2:**
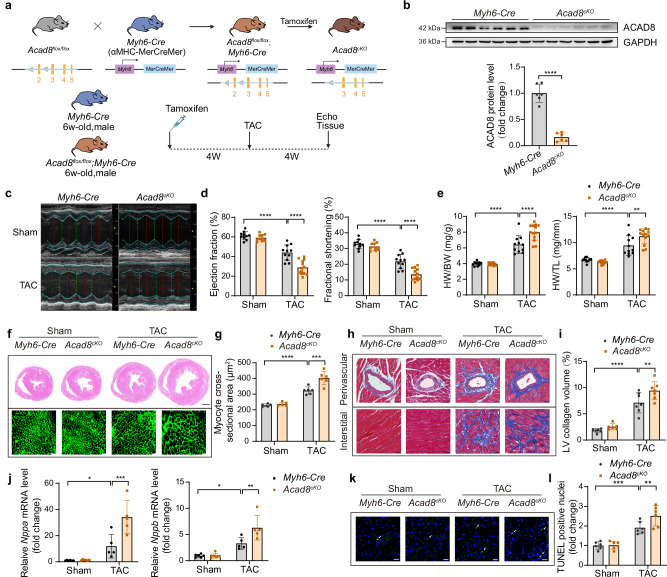
Cardiomyocyte-specific *Acad8* knockout exacerbates TAC-induced pathological cardiac hypertrophy. **a** Schematic overview of the establishment of the cardiomyocyte-specific *Acad8* knockout mice and the TAC-induced cardiac hypertrophy mouse model. Created in BioRender. Lab, 5. (2026) https://BioRender.com/cmg81kg. **b** Western blotting (top) and quantification (bottom) of ACAD8 protein expression in the hearts of *Myh6-Cre* and *Acad8*^*cKO*^ mice. ****P <  0.0001, by two-tailed unpaired Student’s *t*-test. *n * =  6 mice/group. **c** Representative echo images of *Myh6-Cre* and *Acad8*^*cKO*^ mice 4 weeks after Sham or TAC surgery. **d**,**e** Ejection fraction, fractional shortening (**d**), heart weight-to-body weight (HW/BW), and heart weight-to-tibia length (HW/TL) (**e**) of *Myh6-Cre* and *Acad8*^*cKO*^ mice 4 weeks after Sham or TAC surgery. ****P  <  0.0001, **P  =  0.0027, by two-way ANOVA with Bonferroni *post hoc* correction. *n * =  10 (*Myh6-Cre*-Sham), 10 (*Acad8*^*cKO*^-Sham), 11 (*Myh6-Cre*-TAC), 13 (*Acad8*^*cKO*^-TAC) mice/group. **f** Representative images of H&E staining (top) and WGA staining (bottom) of the hearts of *Myh6-Cre* and *Acad8*^*cKO*^ mice 4 weeks after Sham or TAC surgery. Scale bars: 1 mm (top) and 30 μm (bottom). **g** Quantification of the cardiomyocyte cross-sectional area based on WGA staining. ****P  <  0.0001, ***P  =  0.0001, by two-way ANOVA with Bonferroni *post hoc* correction. *n*  =  5 (*Myh6-Cre*-Sham), 5 (*Acad8*^*cKO*^-Sham), 6 (*Myh6-Cre*-TAC), 6 (*Acad8*^*cKO*^-TAC) mice/group. **h** Representative image of Masson staining and **i**, quantification of cardiac fibrosis. ****P  <  0.0001, **P  =  0.0097, by two-way ANOVA with Bonferroni *post hoc* correction. *n * =  6 (*Myh6-Cre*-Sham), 6 (*Acad8*^*cKO*^-Sham), 7 (*Myh6-Cre*-TAC), 7 (*Acad8*^*cKO*^-TAC) mice/group. Scale bar: 20 μm. **j** mRNA expression levels of hypertrophic genes. From left to right: *P  = 0.0494, ***P  = 0.0003, *P  = 0.0116, **P  = 0.0028, respectively, by two-way ANOVA with Bonferroni *post hoc* correction. *n*  =  6 (*Myh6-Cre*-Sham), 6 (*Acad8*^*cKO*^-Sham), 5 (*Myh6-Cre*-TAC), 5 (*Acad8*^*cKO*^-TAC) mice/group. **k** Representative images and **l**, quantitative analysis of TUNEL staining. ***P  = 0.0003, **P  = 0.0098, by two-way ANOVA with Bonferroni *post hoc* correction. *n * =  6 mice/group. Arrows indicate TUNEL-positive cells (green) colocalized with DAPI-stained nuclei (blue). Scale bar: 20 μm. Data are presented as mean values  ±  SD. Source data are provided as a Source Data file.

To investigate the role of valine catabolism and ACAD8 in pathological cardiac hypertrophy, we generated a cardiomyocyte-specific *Acad8*-knockout mouse (*Acad8*^*cKO*^) model. We induced pathological cardiac hypertrophy in these mice with TAC surgery (Fig. 2a). Four weeks after tamoxifen-induced gene knockout, the ACAD8 protein expression in the hearts of *Acad8*^*cKO*^ mice was significantly lower than that in the hearts of control mice (Fig. 2b).

Early cardiac response can be observed as early as 3–7 days after TAC surgery^22–24^. We observed that *Acad8*^*cKO*^ mice exhibited more pronounced cardiac hypertrophy compared to *Myh6-Cre* controls five days post TAC surgery (Supplementary Fig. 1a–c), accompanied by significant functional impairment (Supplementary Fig. 1d, f). To fully evaluate the effects of ACAD8 on pathological response to pressure overload, we next performed further assessments at 4 weeks post-TAC. Cardiac function assessment via echocardiography revealed that, compared with *Myh6-Cre* mice, *Acad8*^*cKO*^ mice exhibited exacerbated TAC-induced cardiac dysfunction, as manifested by decreases in the cardiac ejection fraction (EF) and fractional shortening (FS) (Fig. 2c, d). Additionally, the heart weight-to-body weight (HW/BW) and heart weight-to-tibia length (HW/TL) ratios were further increased in *Acad8*^*cKO*^ mice compared with *Myh6-Cre* mice after TAC surgery (Fig. 2e). We subsequently analysed the cross-sectional areas of mouse hearts and the surface areas of cardiomyocytes by haematoxylin‒eosin (H&E) and wheat germ agglutinin (WGA) staining, respectively. The results revealed that the cross-sectional areas and cardiomyocyte sizes were significantly increased in the hearts of *Acad8*^*cKO*^ mice after TAC surgery (Fig. 2f, g). Moreover, Masson staining revealed increased cardiac fibrosis in *Acad8*^*cKO*^ mice after TAC surgery (Fig. 2h, i). Finally, qRT-PCR analysis revealed increased expression of hypertrophy marker genes (*Nppa* and *Nppb*) in *Acad8*^*cKO*^ mice after TAC (Fig. 2j). Cardiac hypertrophy and heart failure progression are frequently accompanied by cell death^25^. Thus we also assessed apoptosis in cardiac tissue using TUNEL assays. The results demonstrated that *Acad8*^*cKO*^ mice exhibited exacerbated TAC-induced cell death compared to *Myh6-Cre* controls (Fig. 2k, l).

The *Acad8*^*cKO*^ mice did not exhibit spontaneous cardiac hypertrophy within eight weeks of analysis post-tamoxifen injection. However, these hearts showed significantly enhanced susceptibility to pressure overload-induced myocardial hypertrophy following TAC surgery. RNA sequencing revealed that *Acad8*^*cKO*^ hearts under baseline conditions exhibited upregulation of cardiomyopathy-related genes, along with elevated expression of specific cardiac hypertrophy markers (e.g., *Nppa*, *Ankrd1*) (Supplementary Fig. 1g, h). Additionally, genes associated with Ca²⁺ handling (e.g., *Atp2a2*, *Ryr2*) and fibrosis-related genes (e.g., *Nfkb1*) also displayed an upward trend, suggesting a predisposition to contraction dysfunction and fibrosis (Supplementary Fig. 1j). Collectively, these findings demonstrate that ACAD8 deficiency directly promotes pathological cardiac hypertrophy.

### ACAD8 deficiency in TAC-operated hearts leads to a significant accumulation of isobutyryl-CoA and promotes histone isobutyrylation

**Fig. 3:**
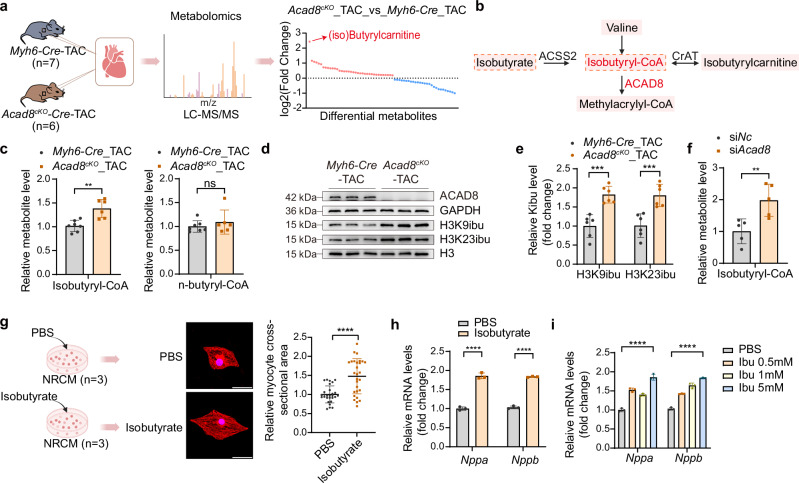
*Acad8* deficiency promotes both isobutyryl-CoA accumulation and histone isobutyrylation and isobutyryl-CoA donor promotes cardiomyocyte hypertrophy. **a** Schematic of metabolomics-based experimental design for left ventricles of *Myh6-Cre*-TAC and *Acad8*^*cKO*^-TAC hearts. The scatter plot shows the variation in the difference multiples of the identified significantly differentially abundant metabolites. Created in BioRender. Lab, 5. (2026) https://BioRender.com/25zpni7. **b** Schematic displaying key metabolic reactions that directly regulate isobutyryl-CoA levels. Isobutyryl-CoA can be produced via the valine catabolic pathway and is converted into methylacrylyl-CoA by ACAD8. In addition, isobutyrate can be metabolised by ACSS2 to yield isobutyryl-CoA. CrAT interconverts short-chain acyl-CoAs and their corresponding acylcarnitines. ACAD8, acyl-CoA dehydrogenase family member 8; ACSS2, acetyl-CoA synthetase 2; CrAT, carnitine acetyltransferase. **c** MS-based quantification of isobutyryl-CoA and n-butyryl-CoA levels in mice hearts. **P = 0.0015, *P* = 0.5459, ns, not significant, by two-tailed unpaired Student’s *t*-test. *n * =  7 (*Myh6-Cre*-TAC), 6 (*Acad8*^*cKO*^-TAC) mice/group. **d** Western blotting with GAPDH serves as a loading control and **e** Quantification of H3k9ibu and H3k23ibu levels in mice hearts. From left to right: ***P  = 0.0001, ***P  = 0.0002, respectively, by two-way ANOVA with Bonferroni *post hoc* correction. *n * =  6 mice/group. **f** MS-based quantification of isobutyryl-CoA levels in NRCMs transfected with si*Nc* or si*Acad8*. **P = 0.0091, by two-tailed unpaired Student’s *t*-test. *n * = 5 mice/group. **g** Representative images and quantification of the sizes of NRCM cell size after treatment with PBS or isobutyrate (5 mM). Scale bar: 30 μm. ****P <  0.0001, by two-tailed Mann-Whitney test. *n*  =  28 cells/group. Created in BioRender. Lab, 5. (2026) https://BioRender.com/huf3kbv. **h** mRNA expression of hypertrophic genes in NRCMs after isobutyrate treatment with PBS or isobutyrate (5 mM). ****P  <  0.0001, by two-way ANOVA with Bonferroni *post hoc* correction. *n * =  3 biologically independent samples. **i**, mRNA expression of hypertrophic genes in NRCMs after isobutyrate treatment with increasing concentrations of isobutyrate. ****P  <  0.0001, by two-way ANOVA with Bonferroni *post hoc* correction. *n*  =  3 biologically independent samples. Data are presented as mean values  ±  SD. Source data are provided as a Source Data file.

Intracellular metabolites play crucial roles in cell signalling, gene expression and cell growth^19,26,27^. To explore the mechanism underlying cardiac pathological hypertrophy in *Acad8*^*cKO*^ mice, we performed targeted metabolomic analysis of hearts from both *Myh6-Cre* and *Acad8*^*cKO*^ mice that were subjected to TAC surgery (Fig. 3a). A total of 294 metabolites were identified during the analysis. Based on the significance threshold of p value < 0.05, 64 differentially abundant metabolites were identified; among these metabolites, we observed a marked increase in the abundance of (iso)butyrylcarnitine (a mixture of two isomers: n-butyrylcarnitine and isobutyrylcarnitine) in the hearts of *Acad8*^*cKO*^ mice that were subjected to TAC (Fig. 3a). Acylcarnitines are important lipid biomarkers that reflect the acyl-CoA status^28^. Notably, *Acad8* deficiency leads to the accumulation of its substrate, isobutyryl-CoA, that binds to carnitine to form isobutyrylcarnitine; this process clinically manifests as an increase in the (iso)butyrylcarnitine levels^14,29^ (Fig. 3b). We further validated the accumulation of isobutyrylcarnitine in *Acad8*-deficient hearts (Supplementary Fig. 2a).

Based on the above findings, we hypothesised that isobutyryl-CoA may serve as a metabolic signal driven by *Acad8* deficiency and promotes pathological cardiac hypertrophy. To test this hypothesis, we measured the content of isobutyryl-CoA with mass spectrometry (MS). *Acad8*^*cKO*^ hearts post TAC surgery showed significantly elevated isobutyryl-CoA levels compared to control mice (Fig. 3c and Supplementary Fig. 2b). Although previous studies have demonstrated the level of isobutyryl-CoA is much higher than n-butyryl-CoA in mammalian cells^15^, we also measured the levels of n-butyryl-CoA and exclude potential interference from n-butyryl signals in our metabolic data. The results showed that *Acad8* deficiency did not affect n-butyryl-CoA levels in the hearts of TAC-operated *Acad8*^*cKO*^ mice and in *Acad8* knockdown NRCMs (Fig. 3c and Supplementary Fig. 3a). Furthermore, we observed a global elevation in protein isobutyrylation, including both histone and non-histone proteins, in TAC-operated hearts (Supplementary Fig. 2c, d). Intracellular isobutyryl-CoA can act as an acyl donor for lysine isobutyrylation on histone H3^15^. Indeed, we observed elevated isobutyrylation of H3K9 and H3K23 (H3K9ibu and H3K23ibu) in TAC-operated *Acad8*^*cKO*^ mouse hearts (Fig. 3d, e). Additionally, we also provided evidence that siRNA-mediated *Acad8* knockdown increased the isobutyryl-CoA levels (Fig. 3f), which enhanced H3K9ibu and H3K23ibu in NRCMs (Supplementary Fig. 3b–d).

To understand the pathological effect of isobutyryl-CoA on cardiomyocytes, we treated NRCMs with the isobutyryl-CoA donor isobutyrate, and we observed that isobutyrate promoted cardiomyocyte hypertrophy and induced hypertrophy-associated gene expression (Fig. 3g, h). Furthermore, isobutyrate increased the expression of hypertrophy-associated genes in NRCMs in a dose-dependent manner (Fig. 3i), with no significant effects on cell viability (Supplementary Fig. 3e). Consistent with these findings, treatment with valine also induced a hypertrophic response in vitro. The results showed that NRCMs cultured in media supplemented with increasing concentrations of valine resulted in a dose-dependent upregulation of key hypertrophy-related genes, including *Nppa*, *Nppb*, and *Myh7* (Supplementary Fig. 4a, b). This hypertrophic effect of valine was likely mediated by elevated intracellular levels of isobutyryl-CoA, which was accompanied by increased levels of the H3K9ibu and H3K23ibu in NRCMs (Supplementary Fig. 4c–f). These results collectively indicate that elevated isobutyryl-CoA and histone isobutyrylation play critical roles in promoting the development of cardiac hypertrophy.

Given the mitochondrial localisation of ACAD8, we further investigated whether *Acad8*^*cKO*^ exerted its effects by inducing mitochondrial alterations in cardiac tissue. Transmission electron microscopy (TEM) analysis of cardiac tissue revealed significant reductions in mitochondrial length and mitochondrial density in TAC-operated mice, and *Acad8*^*cKO*^ exacerbated these TAC-induced mitochondrial abnormalities (Supplementary Fig. 5a, b). We further assessed mitochondrial reactive oxygen species (mROS) levels using MitoSOX staining of heart tissue sections. The results revealed that *Acad8*^*cKO*^ significantly exacerbated TAC-induced mROS accumulation in cardiac tissue (Supplementary Fig. 5c, d). We further assessed whether *Acad8* deficiency affects mitochondria-associated metabolites. The levels of acylcarnitines derived from medium- and long-chain fatty acids were significantly reduced in TAC-operated *Acad8*^*cKO*^ hearts, indicating impaired fatty acid oxidation (Supplementary Fig. 5e). While acetyl-CoA levels exhibited a downward trend, this change did not reach statistical significance (Supplementary Fig. 5f). However, circulating Krebs cycle metabolites (e.g., succinate, fumarate, and malate) remained unchanged in TAC-operated *Acad8*^*cKO*^ hearts, and levels of α-ketoglutarate increased, which suggests the presence of metabolic compensation mechanisms (Supplementary Fig. 5g). We found a trend towards increased glycolytic metabolites in TAC-operated *Acad8*^*cKO*^ hearts. Furthermore, significant accumulation of multiple amino acids (e.g., ornithine, phenylalanine, and methionine) and widespread alterations in amino acid turnover-related metabolites were observed (Supplementary Fig. 5h, i). Collectively, *Acad8* deficiency exacerbated TAC-induced mitochondrial impairment in the heart.

### Isobutyryl-CoA accumulation due to ACAD8 deficiency promotes cardiomyocyte hypertrophy via regulating chromatin accessibility and TEAD2 enrichment

**Fig. 4:**
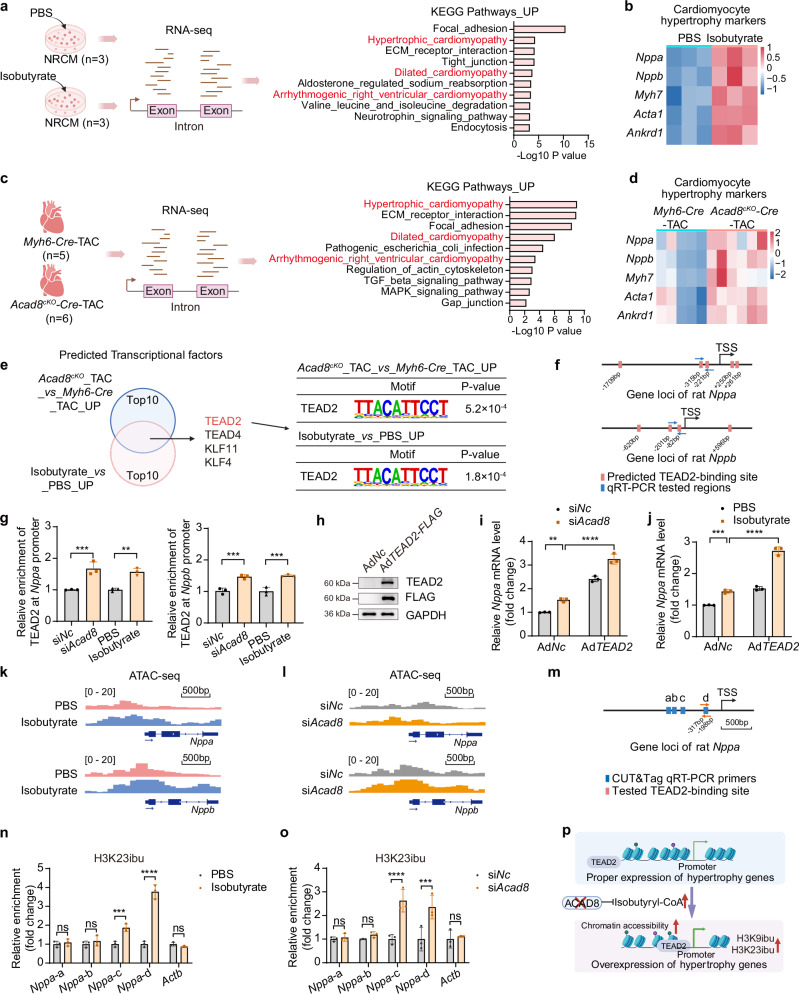
*Acad8* deficiency and isobutyrate promote expression of hypertrophic genes via regulating chromatin accessibility and TEAD2 enrichment. **a**,**b** RNA-seq of NRCMs treated with PBS (*n* = 3) or isobutyrate (*n* = 3), with KEGG enrichment of upregulated genes (**a**); heatmaps of differentially expressed hypertrophic genes (**b**). Differentially expression genes (DEGs) were analysed with The Benjamini-Hochberg (FDR value < 0.05) using DESeq2. Cartoon was created in BioRender. Lab, 5. (2026) https://BioRender.com/huf3kbv. **c**,**d** RNA-seq of left ventricles of *Myh6-Cre*-TAC (*n* = 5) and *Acad8*^*cKO*^-TAC (*n* = 6) hearts, with KEGG enrichment of upregulated genes (**c**); heatmaps of differentially expressed hypertrophic genes (**d**). DEGs were analysed with the Benjamini-Hochberg (FDR value < 0.05) using DESeq2. Cartoon was created in BioRender. Lab, 5. (2026) https://BioRender.com/dps8hl5. **e**, Shared transcription factors in **a** and **b**. DEGs in (**a** and **b)** were subjected for transcription factor prediction using TRANSFAC and JASPAR PWM database. **f** Predicted binding sites and validation regions for TEAD2 in the *Nppa* and *Nppb* promoter regions. Positions relative to the TSS are indicated. **g** CUT&Tag and qRT-PCR analysis of TEAD2 enrichment after isobutyrate treatment or *Acad8* knockdown. From left to right: ***P  = 0.0003, **P  = 0.001, ***P  = 0.0004, ***P  = 0.0002, by one-way ANOVA with Bonferroni *post hoc* correction, n = 3 biologically independent samples. **h** TEAD2 protein expression in NRCMs. GAPDH serves as a loading control. **i, j**, Hypertrophic gene expression in NRCMs after *Acad8* knockdown or isobutyrate treatment, followed by *TEAD2* overexpression. **P  = 0.002, ****P  <  0.0001, ***P  = 0.0002, ns, not significant, by two-way ANOVA with Bonferroni *post hoc* correction, *n* = 3 biologically independent samples. **k, l** IGV images showing ATAC-seq peaks at *Nppa* and *Nppb* loci in NRCMs with isobutyrate treatment or *Acad8* knockdown. **m** Validation sites for isobutyrylation levels at the *Nppa* promoter region. *Nppa*-a region: −882bp ~ −773bp; *Nppa*-b region: −838bp ~ −717bp; *Nppa*-c region: −735bp ~ −608bp; *Nppa*-d region: −317bp ~ −198bp. **n**,**o** CUT&Tag and qRT-PCR analysis for H3K23ibu enrichment at *Nppa* promoter regions after isobutyrate treatment or *Acad8* knockdown. ***P  = 0.0001, ****P  <  0.0001 (**n**), ****P  <  0.0001, ***P  = 0.0002 (**o**), ns, not significant, by two-way ANOVA with Bonferroni *post hoc* correction, *n* = 3 biologically independent samples. **p** ACAD8 deficiency elevates isobutyryl-CoA and histone isobutyrylation, increasing chromatin accessibility, enhancing TEAD2 binding and hypertrophic gene expression. Cartoon was created in BioRender. Lab, 5. (2026) https://BioRender.com/ac6zka9. Data are presented as mean values  ±  SD. Source data are provided as a Source Data file.

We performed RNA sequencing analysis of isobutyrate-treated NRCMs, and KEGG pathway analysis revealed that the expression of genes that are related to multiple cardiomyopathies was upregulated (Fig. 4a). RNA sequencing data also suggested that the expression of cardiomyocyte hypertrophy markers (*Nppa*, *Nppb*, *Myh7*, *Acta1*, and *Ankrd1*) was significantly increased after isobutyrate treatment (Fig. 4b). The RNA sequencing results revealed that the hearts of *Acad8*^*cKO*^ mice that were subjected to TAC exhibited an upregulation of cardiomyopathy pathway-related genes and cardiac hypertrophy markers (Fig. 4c, d). TRANSFAC and JASPAR PWM analyses were performed to identify transcription factors regulating the genes upregulated in the two datasets described above. The results identified TEA domain transcription factor 2 (TEAD2) as the most promising transcription factor that could participate in the effects of both *Acad8* deficiency and isobutyryl-CoA accumulation (Fig. 4e). TEAD2 is a member of the TEA domain family. It plays important roles in cardiac hypertrophy^30,31^. Using the JASPAR database for transcription factor binding site prediction, we identified potential TEAD2 binding motifs within the promoter regions of both *Nppa* and *Nppb* (Fig. 4f). Subsequent CUT&Tag assays combined with qRT-PCR validation demonstrated that both isobutyrate treatment and *Acad8* knockdown significantly enhanced TEAD2 binding in the promoter regions of *Nppa* and *Nppb* (Fig. 4f, g). To validate the function of TEAD2 in cardiac hypertrophy, we overexpressed it in NRCMs and found that TEAD2 overexpression (OE) promoted the expression of *Nppa* induced by *Acad8* knockdown and isobutyrate treatment (Fig. 4h–j).

However, neither isobutyrate treatment nor *Acad8* deficiency significantly altered *Tead2* expression levels (Supplementary Fig. 6a, b). Histone modifications play pivotal roles in cardiac pathophysiology by dynamically regulating chromatin architecture and transcriptional programs^17,19,32,33^. Increased isobutyryl-CoA and isobutyrylation modifications may promote chromatin opening, potentially enhancing TEAD2 binding at the promoters of hypertrophy genes. To test this hypothesis, we further used ATAC-seq to assess changes in the chromatin accessibility of the *Nppa* and *Nppb* genes. The results showed that isobutyrate treatment resulted in increased chromatin accessibility in the promoter regions of *Nppa* and *Nppb* (Fig. 4k). Additionally, *Acad8* knockdown increased chromatin accessibility at the promoter regions of *Nppa* and *Nppb* (Fig. 4l). We further confirmed that both isobutyrate treatment and *Acad8* knockdown promoted the enrichment of H3K9ibu and H3K23ibu at the promoter region of the hypertrophy-related gene *Nppa* (Fig. 4m–o and Supplementary Fig. 7a–c). These findings suggest that *Acad8* deficiency causes cardiomyocyte hypertrophy via the accumulation of isobutyryl-CoA to regulate histone isobutyrylation, chromatin accessibility and TEAD2 enrichment at promoter regions of targeted genes (Fig. 4p).

### ACAD8 overexpression attenuates TAC-induced cardiac hypertrophy

**Fig. 5:**
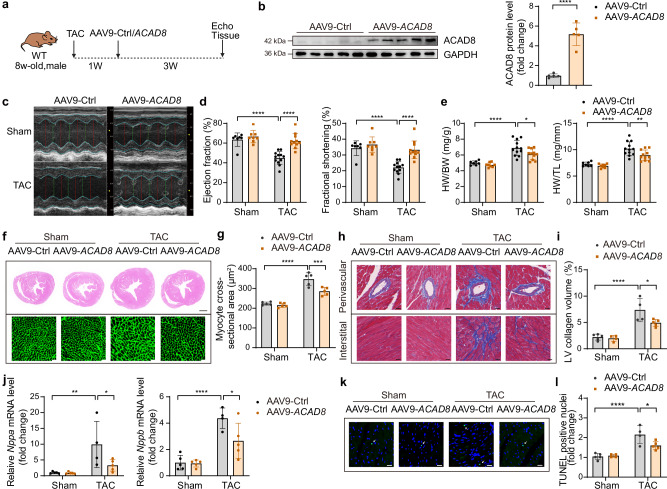
AAV9-mediated ACAD8-OE in cardiomyocytes ameliorates TAC-induced pathological cardiac hypertrophy. **a** Schematic of AAV9-cTnT-mediated cardiomyocyte-specific ACAD8-OE and TAC-induced cardiac hypertrophy mouse model. Created in BioRender. Lab, 5. (2026) https://BioRender.com/w01zyqt. **b** ACAD8 protein overexpression efficiency of AAV9-cTnT-*ACAD8* in the hearts of TAC mice. ****P  <  0.0001, by two-tailed unpaired Student’s *t*-test. *n * =  5 mice/group. **c** Representative echo images of mice infected with AAV9-cTnT-Ctrl or AAV9-cTnT-*ACAD8* after Sham or TAC surgery. **d**,**e** Ejection fraction, fractional shortening, heart weight-to-body weight (HW/BW), and heart weight-to-tibia length (HW/TL) of mice infected with AAV9-cTnT-Ctrl or AAV9-cTnT-*ACAD8* after Sham or TAC surgery. ****P  <  0.0001, *P  =  0.02, **P  =  0.0082, by two-way ANOVA with Bonferroni *post hoc* correction. n  =  7 (AAV9-cTnT-Ctrl-Sham), 8 (AAV9-cTnT-*ACAD8*-Sham), 12 (AAV9-cTnT-Ctrl-TAC), 11 (AAV9-cTnT-*ACAD8*-TAC) mice/group. **f** H&E (top) staining and WGA (bottom) staining of the hearts. Scale bars: 1 mm (top) and 30 μm (bottom). **g** Quantification of the cardiomyocyte cross-sectional area based on WGA staining in the hearts. ****P  <  0.0001, ***P  =  0.0004, by two-way ANOVA with Bonferroni *post hoc* correction. n  =  5 mice/group. **h** Representative image of Masson staining and **i** quantification of cardiac fibrosis in mice. Scale bar: 20 μm. ****P  <  0.0001, *P  =  0.0135, by two-way ANOVA with Bonferroni *post hoc* correction. *n * =  5 (AAV9-cTnT-Ctrl-Sham), 4 (AAV9-cTnT-*ACAD8*-Sham), 4 (AAV9-cTnT-Ctrl-TAC), 5 (AAV9-cTnT-*ACAD8*-TAC) mice/group. **j** mRNA expression of hypertrophic genes. From left to right: **P  = 0.0028, *P  = 0.0223, ****P  <  0.0001, *P  = 0.0161, respectively, by one-way ANOVA with Bonferroni *post hoc* correction. *n*  =  5 (AAV9-cTnT-Ctrl-Sham), 5 (AAV9-cTnT-*ACAD8*-Sham), 4 (AAV9-cTnT-Ctrl-TAC), 5 (AAV9-cTnT-*ACAD8*-TAC) mice/group. **k** Representative images and **l** quantitative analysis of TUNEL staining in heart sections. Arrows indicate TUNEL-positive cells (green) colocalized with DAPI-stained nuclei (blue). scale bar: 20 μm. ****P  <  0.0001, *P  =  0.0119, by two-way ANOVA with Bonferroni *post hoc* correction. *n * =  5 (AAV9-cTnT-Ctrl-Sham), 4 (AAV9-cTnT-*ACAD8*-Sham), 4 (AAV9-cTnT-Ctrl-TAC), 5 (AAV9-cTnT-*ACAD8*-TAC) mice/group. Data are presented as mean values  ±  SD. Source data are provided as a Source Data file.

Next, we investigated whether ACAD8-OE in cardiomyocytes could suppress pathological cardiac hypertrophy. We established a mouse model of TAC-induced pathological cardiac hypertrophy, and subsequently administered intravenous injections of AAV9-cTnT-Ctrl or AAV9-cTnT-*ACAD8* (where cTnT is a cardiomyocyte-specific promoter) seven days after TAC (Fig. 5a). The efficiency of AAV9-cTnT-*ACAD8*-OE was verified at the protein level (Fig. 5b). Cardiomyocyte-specific ACAD8-OE effectively suppressed TAC-induced cardiac dysfunction, as shown by improvements in the EF and FS (Fig. 5c, d) as well as an reduce in the heart weight (Fig. 5e). Histological assessments via H&E staining, Masson staining and WGA staining confirmed that cardiac hypertrophy and fibrosis were reduced by AAV9-cTnT-*ACAD8* in mice with cardiac hypertrophy (Fig. 5f–i). The expression levels of hypertrophy-related genes were also decreased in TAC model mice following AAV9-cTnT-*ACAD8* treatment (Fig. 5j). Additionally, cardiomyocyte-specific ACAD8-OE attenuated TAC-induced cell death compared to AAV9-control mice, as evidenced by reduced TUNEL^+^ cardiomyocytes (Fig. 5k, l). Collectively, these findings demonstrate that cardiomyocyte-specific ACAD8-OE inhibited TAC-induced cardiac hypertrophy and restored cardiac function.

### ACAD8-OE protects against TAC-induced pathological cardiac hypertrophy by reducing isobutyryl-CoA levels and attenuating histone isobutyrylation

**Fig. 6:**
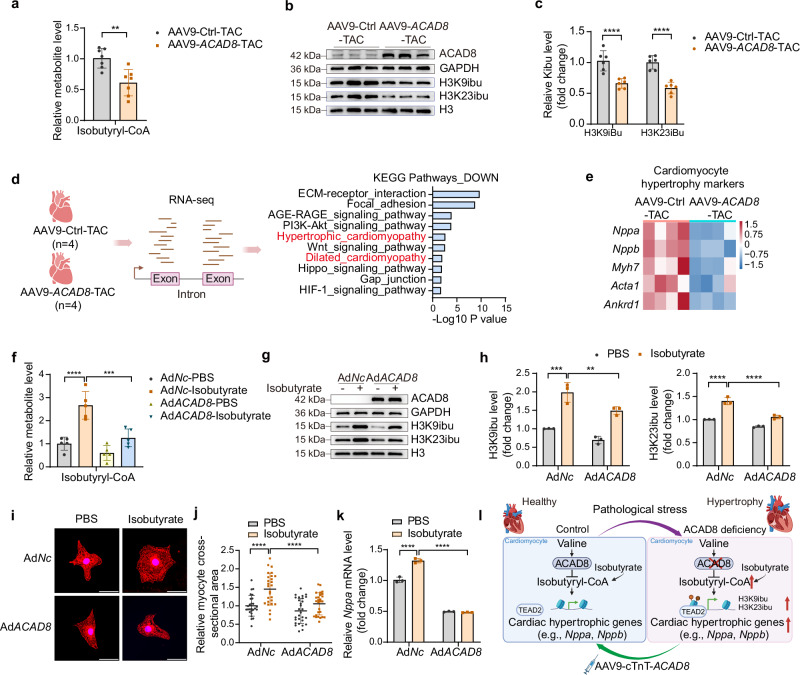
ACAD8-OE protects against TAC-induced pathological cardiac hypertrophy by reducing isobutyryl-CoA levels. **a** Quantification of isobutyryl-CoA levels in AAV9-Ctrl-TAC and AAV9-*ACAD8*-TAC hearts. **P  <  0.0019, by two-tailed unpaired Student’s *t*-test. *n*  =  7 mice/group. **b** Western blotting with GAPDH serves as a loading control and **c** Quantification of H3K9ibu and H3K23ibu levels in in AAV9-Ctrl-TAC and AAV9-*ACAD8*-TAC hearts. ****P  <  0.0001, by two-way ANOVA with Bonferroni *post hoc* correction. *n*  =  6 mice/group. **d** Schematic of RNA-seq design for left ventricles of AAV9-Ctrl-TAC and AAV9-*ACAD8*-TAC hearts, with KEGG enrichment analysis of downregulated genes. Created in BioRender. Lab, 5. (2026) https://BioRender.com/j890fmd. **e** Heatmaps of differentially expressed hypertrophic genes. **f**, Isobutyryl-CoA levels in NRCMs infected with the indicated adenovirus, followed by PBS or isobutyrate treatment. ****P  <  0.0001, ***P  =  0.001, by two-way ANOVA with Bonferroni *post hoc* correction. n  =  5 mice/group. **g** Western blotting with GAPDH serves as a loading control and **h** Quantification of H3K9ibu and H3K23ibu levels in NRCMs treated with PBS or isobutyrate (5 mM) after infection with Ad*Nc* or Ad*ACAD8* (*n* = 3; **p < 0.01, ****p < 0.0001). ***P  =  0.0001, **P  =  0.0083, ****P  <  0.0001, by two-way ANOVA with Bonferroni *post hoc* correction. *n * =  3 biologically independent samples. **i**,**j** Representative images and quantification of NRCM cell size after Ad*Nc* or Ad*ACAD8* infection, followed by PBS or isobutyrate treatment. Scale bar: 30 μm. ****P  <  0.0001, by two-way ANOVA with Bonferroni *post hoc* correction. *n * =  26 (Ad*Nc*-PBS), 27 (Ad*ACAD8*-PBS), 29 (Ad*Nc*-isobutyrate), 27 (Ad*ACAD8*-isobutyrate) cells/group. **k**, Nppa mRNA expression in NRCMs after Ad*Nc* or Ad*ACAD8* infection, followed by PBS or isobutyrate treatment. ****P  <  0.0001, by two-way ANOVA with Bonferroni *post hoc* correction. *n*  =  3 biologically independent samples. **l** Summary graph showing the mechanism by which ACAD8 deficiency exacerbates TAC-induced pathological cardiac hypertrophy. ACAD8 deficiency in cardiomyocytes leads to the accumulation of isobutyryl-CoA, histone isobutyrylation, chromatin opening and TEAD2 enrichment, which promote the expression of hypertrophy-associated genes. Conversely, ACAD8-OE attenuates cardiac hypertrophy by reducing isobutyryl-CoA levels. Created in BioRender. Lab, 5. (2026) https://BioRender.com/b0r07g2. Data are presented as mean values  ±  SD. Source data are provided as a Source Data file.

To investigate the mechanistic basis underlying the protective effects of *ACAD8*-OE against pathological cardiac hypertrophy, we first measured the levels of isobutyryl-CoA in AAV9-Ctrl-TAC and AAV9-*ACAD8*-TAC hearts and the results showed that *ACAD8*-OE reduced the levels of isobutyryl-CoA in the heart (Fig. 6a). We also validated the effect of *ACAD8*-OE on the isobutyrylation of histone H3. Our results revealed that ACAD8-OE led to a decrease in isobutyrylation of H3K9 and H3K23 in hearts of TAC-challenged mice (Fig. 6b, c). We further performed RNA sequencing on hearts from AAV9-Ctrl-TAC and AAV9-*ACAD8*-TAC mice. KEGG pathway analysis revealed the downregulation of genes related to various cardiomyopathies and a significant reduction in cardiac hypertrophy markers in AAV9-*ACAD8*-TAC hearts (Fig. 6d, e). We also found that acetyl-CoA levels showed an increase in AAV9-*ACAD8*-TAC hearts, potentially attributable to overall improvements in cellular metabolism (Supplementary Fig. 8a). TEM of cardiac tissue showed that *ACAD8*-OE ameliorated the TAC-induced reductions in mitochondrial length and mitochondrial density (Supplementary Fig. 8b, c). Additionally, *ACAD8*-OE alleviated TAC-induced mROS accumulation in cardiac tissues (Supplementary Fig. 8d, e), which was consistent with a rebound of endogenous *Acad8* expression (Supplementary Fig. 8f).

The further MS data showed that ACAD8-OE reduced intracellular isobutyryl-CoA accumulation (Fig. 6f). ACAD8-OE also rescued the isobutyrate-induced increase in isobutyrylation of H3K9 and H3K23 (Fig. 6g, h). We next investigated whether ACAD8-OE in NRCMs could directly mitigate the effects of isobutyrate on cardiomyocyte hypertrophy. The results revealed that ACAD8-OE could directly repress isobutyrate-induced increase in cell size and expression of hypertrophic marker genes (Fig. 6i–k). Taken together, these findings suggest that ACAD8-OE ameliorates pathological cardiac hypertrophy by reducing isobutyryl-CoA levels and histone isobutyrylation.

## Discussion

In this study, we demonstrated that ACAD8 expression was decreased in the hearts of patients with hypertrophic cardiomyopathy and mice with cardiac hypertrophy. Using a mouse model of TAC-induced cardiac hypertrophy, we found that cardiomyocyte-specific *Acad8* knockout promoted TAC-induced cardiac hypertrophy and dysfunction. *Acad8* deficiency led to isobutyryl-CoA accumulation, histone isobutyrylation, chromatin opening and TEAD2 enrichment at regions of target genes, subsequently promoted the sensitivity of cardiomyocytes and hearts to hypertrophic stress. Furthermore, AAV9-mediated cardiomyocyte-specific ACAD8 overexpression reversed the TAC-induced cardiac hypertrophy and cardiac dysfunction.

BCAA levels and their metabolites are associated with cardiovascular disease^11,21,34–36^. The accumulation of BCAA and BCKA due to defective BCAA catabolism promotes the development of cardiac hypertrophy and heart failure through multiple mechanisms^34,37–40^. Currently available methods for studying branched-chain amino acids often involve models in which metabolism-associated genes that are shared by the three kinds of BCAAs are knocked out^34,38,41^. However, knocking out these genes may have confounding effects, as evidence from several studies indicates distinct physiological and metabolic effects for each of the three BCAAs^42–44^. Therefore, elucidating the respective roles of the metabolism of these three kinds of BCAAs in pathological cardiac hypertrophy and heart failure is warranted. Our study highlights the critical role of valine catabolism in cardiac hypertrophy. We observed reduced expression of valine catabolism-associated genes in the hearts of patients with hypertrophic cardiomyopathy and mice with cardiac hypertrophy. However, the other BCAA metabolic pathways may also play some roles in cardiac hypertrophy. Given that the three BCAAs share some metabolic enzymes, potential interactions between these pathways likely exist. Future studies should systematically investigate the distinct roles of valine, leucine, and isoleucine metabolism in hypertrophic remodelling to elucidate their specific contributions.

To further investigate the correlation between valine metabolism and cardiac hypertrophy, we focused on ACAD8, which regulates the critical third step of valine catabolism. The protein levels of ACAD8 were significantly reduced in the hearts of patients with hypertrophic cardiomyopathy and mice with cardiac hypertrophy. *Acad8* knockdown in NRCMs directly induced cardiomyocyte hypertrophy. To further investigate the contribution of intracellular valine metabolism to pathological myocardial hypertrophy, we used cardiomyocyte-specific *Acad8*-knockout mice for further studies. While no overt cardiac phenotype was observed under non-stressed conditions during the study period (eight weeks post-tamoxifen injection), *Acad8* deficiency upregulated hypertrophy-related genes at baseline and increased susceptibility to pathological cardiac remodelling when exposed to pressure overload. Whether long-term *Acad8* deficiency could lead to spontaneous cardiac hypertrophy warrants further investigation.

Valine metabolic intermediates, such as 3-hydroxyisobutyric acid (3-HIB), monomethyl-branched-chain fatty acid derivatives, and propionyl-CoA, are involved in metabolic and cardiovascular diseases^8,9,18,43,45,46^. Our study revealed that isobutyryl-CoA levels were significantly increased in the hearts of *Acad8*-deficient mice and in *Acad8* knockdown NRCMs. This finding provides key pathological insights for ACAD8 deficiency and isobutyryl-CoA deregulation in human patients^14,16^. Our results demonstrated that *Acad8* deficiency exacerbates TAC-induced hypertrophy by promoting isobutyryl-CoA accumulation. Future studies will specifically investigate the levels of isobutyryl-CoA in hypertrophic cardiac tissues to elucidate its clinical significance further. The direct linking between isobutyryl-CoA and cardiomyocyte hypertrophy was confirmed in vitro because isobutyryl-CoA donor isobutyrate can directly increase cardiomyocyte size and expression of hypertrophic genes. Based on our data and previous studies, the isobutyryl-CoA level induced by exogenous isobutyrate in cardiomyocytes in vitro in this study is comparable with those responses in heart tissues in vivo^15,18^. Given the complexity of in vivo metabolic networks, isobutyrate treatment may partially recapitulate in vivo physiopathological isobutyryl-CoA-mediated effects, but we cannot completely exclude additional effects of isobutyrate. As well, while our study focused on isobutyryl-CoA accumulation as the primary mediator linking ACAD8 deficiency to cardiac hypertrophy, we acknowledge that other downstream metabolites in valine catabolism may also contribute to cardiac hypertrophy. Future studies should investigate whether these metabolites accumulate in hypertrophic hearts and define their specific roles in disease progression.

Isobutyryl-CoA can act as an acyl donor for isobutyrylation of histones, and isobutyrate treatment affects a wide range of cellular processes^15,47^. Our study demonstrated that the isobutyrylation of histone H3K9 and H3K23 were significantly elevated in the hearts of *Acad8*-deficient mice subjected to TAC surgery. Similarly, knockdown of *Acad8* in NRCMs promoted isobutyrate-induced isobutyrylation of H3K9 and H3K23. Both isobutyrate treatment and *Acad8* knockdown promoted the enrichment of H3K9ibu and H3K23ibu at the promoter region of the hypertrophy-related gene *Nppa*. This finding directly links the alterations in H3K9ibu/H3K23ibu to the transcriptional control of hypertrophy-related genes, offering mechanistic insight for the role of *Acad8* deficiency in driving pathological cardiac hypertrophy. Future studies should further elucidate the regulatory mechanisms of histone isobutyrylation on chromatin remodelling and gene expression, including direct profiling of histone isobutyrylation in cardiac tissue to further validate their functional roles in vivo.

The transcription factor TEA domain family (TEAD1–4) are key regulators of cardiovascular development and pathological hypertrophic responses^30,48,49^. The role of TEAD2 in pathological cardiac hypertrophy has recently been demonstrated^31,50^. Here we identified TEAD2 as the key transcriptional regulator responding to histone isobutyrylation. Both isobutyrate treatment and knockdown of *Acad8* in NRCMs promoted TEAD2 binding at the promoter regions of *Nppa* and *Nppb*. ATAC-seq revealed that isobutyrate treatment and *Acad8* deficiency resulted in chromatin opening at the promoter regions of *Nppa* and *Nppb*, which may explain the enhanced binding of TEAD2. These findings suggest that TEAD2 may act as a responder to isobutyryl-CoA and histone isobutyrylation, as thus play a regulatory role in the transcriptional control of genes involved in hypertrophy. Collectively, *Acad8* deficiency leads to isobutyryl-CoA accumulation and histone isobutyrylation, which may alter chromatin accessibility and the binding of transcription factors such as TEAD2 within the promoter regions of hypertrophic genes, thereby modulating gene expression.

It is noteworthy that the deficiency of *Acad8* not only promotes cardiac hypertrophy but also upregulates genes associated with cardiac dysfunction and impairs contractile performance, thereby exacerbating the pathological cardiac hypertrophy. Moreover, cardiomyocyte-specific *Acad8* knockout exacerbated TAC-induced mitochondrial structural and functional impairments. Clinically, patients with ACAD8 mutations exhibit reduced levels of flavins, which may impair electron transport chain activity, thereby affecting cellular energy production and contributing to the generation of ROS^51^. *Acad8* mutant mice display increased hepatic steatosis and mitochondrial damage^52^. These facts suggest that ACAD8 deficiency may contribute to cardiac pathology through mechanisms beyond epigenetic reprogramming. While our study provides strong evidence linking isobutyryl-CoA accumulation to histone isobutyrylation and transcriptional activation, the current data do not exclude other contributing mechanisms. For instance, similar to other acyl-CoA species, isobutyryl-CoA could directly modify non-histone proteins, such as metabolic enzymes or contractile components, thereby influencing cardiac function through alternative pathways^33,53^.

Metabolic therapies have great potential for treating heart failure and pathological cardiac hypertrophy^54^. As personalised treatment approaches gain prominence in the management of heart failure, the demand for novel drugs continues to increase. The development of novel targets for safe and effective metabolic therapies remains a critical challenge. Notably, we propose that ACAD8 is a novel target for the treatment of pathological cardiac hypertrophy. The effect of isobutyryl-CoA on inducing the development of cardiomyocyte hypertrophy can be reversed by ACAD8-OE. Further, the administration of AAV9-*ACAD8* partially alleviated the symptoms of TAC-induced cardiac hypertrophy and improved cardiac function in mice. Our results demonstrate that ACAD8-OE significantly reduces isobutyryl-CoA levels and histone isobutyrylation modifications in TAC-induced hypertrophic hearts, concurrently suppressing the expression of hypertrophy-related genes.

In conclusion, our study highlights the role of ACAD8 in regulating isobutyryl-CoA levels to maintain cardiac homoeostasis (Fig. 6l). *Acad8* deficiency-induced isobutyryl-CoA accumulation exacerbates pressure overload-induced pathological cardiac hypertrophy by epigenetic reprogramming. This ACAD8-isobutyryl-CoA-isobutyrylation axis represents a novel therapeutic target for cardiac hypertrophy.

## Methods

### Human heart samples

Hypertrophic cardiac tissue samples were obtained from patients who underwent surgical resection due to a prior diagnosis of hypertrophic cardiomyopathy. Control cardiac tissue samples were obtained from normal heart donors. The samples were obtained with written informed consent and with the approval of the institutional review boards (Human Research Ethics Committees of Tongji Hospital, Tongji Medical College, Huazhong University of Science and Technology, Wuhan, China) (approval number: TJ-IRB20210829). Residual tissue samples were archived in the Division of Cardiovascular Surgery, Tongji Hospital. Written informed consent to publish potentially identifiable patient information was obtained from all participants. Information of human heart samples is shown in Supplementary Table 1.

### Animals

All the animal protocols were approved by the Animal Care and Use Committee at the Institute of Basic Medical Sciences, Chinese Academy of Medical Sciences, and Peking Union Medical College (approval number: ACUC-A01-2023-059). Sex was not considered as a biological variable in this study. *Acad8*^*flox/flox*^ mice were obtained from the Experimental Animal Tech Co. of Saiye (Beijing, China). The *Myh6-Cre* (αMHC-MerCreMer) transgenic mice were obtained from Jackson Laboratory. Male C57BL/6 mice (8 weeks old) were obtained from Vital River Laboratory Animal Technology Co., Ltd. *Acad8*^*cKO*^ mice were generated by crossing *Acad8*^*flox/flox*^ mice with *Myh6-Cre* (αMHC-MerCreMer) transgenic mice. Conditional cardiomyocyte-specific *Acad8* knockout was induced by tamoxifen treatment. Mice (6–8 weeks old, body weight > 22 g) were intraperitoneally administered either vehicle (oil) or tamoxifen (40 mg/kg) every other day for a total of two doses to induce Cre activity.

### Cardiac hypertrophy model

TAC surgery was used to establish a mouse model of pathological cardiac hypertrophy. Male mice were anaesthetised with isoflurane (1.5–3%). The mouse chest was opened with a longitudinal incision to expose the aortic arch. The aortic arch was ligated at the aortic arch with a 27-gauge needle, and the needle was removed after narrowing. Sham-operated mice underwent similar surgical treatments, but ligation was not performed. Four weeks after TAC surgery, the cardiac function of the mice was evaluated by echocardiography. The mice were executed via decapitation, and the hearts were harvested after perfusion; half of the hearts were fixed, and the remaining hearts were stored at −80 °C for further experiments.

### Construction and infection of AAV9 vectors

The AAV9 recombinant vectors were purchased from Weizhen Biosciences, Inc. The AAV9 vector expressing *ACAD8* was designed such that *ACAD8* was under the control of the *cTnT* promoter. One week after TAC surgery, the mice were injected via the tail vein with either AAV9-cTnT-Ctrl or AAV9-cTnT-*ACAD8* (0.8×10^12^ v.g., 100 μl of viral dilution). The cardiac function of the mice was assessed by echocardiography 4 weeks after TAC.

### Echocardiography analysis

Echocardiography was performed along the parasternal long and short axes with a Vevo 2100 ultrasound imaging system (Visual Sonic) with an MS-400 30-MHz high-resolution ultrasound probe. M-mode images were generated on short-axis and long-axis images at the level of the papillary muscles, and the left ventricular end-systolic and end-diastolic dimensions and thicknesses were measured; these values were used to calculate the ejection fraction and shortening fraction.

### Histological analysis

Freshly harvested hearts were fixed in 4% paraformaldehyde for one day at room temperature, embedded in paraffin, and cut transversely into 5-µm-thick sections. The sections were stained with H&E (Servicebio, G1076) and WGA (Sigma‒Aldrich, L4895) to determine the degree of cardiac hypertrophy and with Masson stain (Servicebio, G1006) to assess cardiac collagen deposition. The stained sections were scanned with a tissue section scanner (3DHISTECH), and the images were analysed using a quantitative digital image analysis system (ImageJ).

### Isolation and culture of NRCMs

NRCMs were isolated from 1–3-day-old SD rats. The rat hearts were removed and immediately placed in precooled HBSS solution, washed to remove the blood, and then cut into 1–3 mm^3^ fragments with ophthalmic scissors. After being washed again, the rat hearts were incubated with digestive enzymes (Thermo, 88281) at 37 °C for approximately 15 min. After digestion was terminated with complete medium, the cells were filtered through a 100-μm cell strainer, and the collected cells were pre-incubated in a cell culture incubator for 1 h to remove noncardiomyocyte cells. The cell suspensions were filtered through a 40-μm cell strainer and centrifuged at 250 × g for 5 min. Then, the cell pellets were resuspended in 5 ml of medium, the cells were counted, and then, the cells were seeded in culture dishes at a density of 2.5 × 10^5^/cm^2^.

NRCMs were cultured in DMEM supplemented with 10% bovine serum, penicillin (100 U/mL)/streptomycin (100 µg/mL)/amphotericin B (25 µg/mL) and 100 µM 5-bromodeoxyuridine (Sigma, B5002) for 24 h, after which the serum concentration was adjusted to 2%.

### Cell transfection and drug treatment

The siRNA that was used to knock down *Acad8* expression was purchased from Synbio Technologies. The targeting sequence for rat *Acad8* was 5’-UCGCCUUGAUACGUCUGUCAUTT-3’ (sense). The transfection procedures were performed according to the manufacturer’s instructions. Briefly, NRCMs were transfected with Lipofectamine RNAiMAX Transfection Reagent (Invitrogen, 13778150), and the medium was replaced with fresh medium 12 h after transfection.

A recombinant adenovirus encoding *Acad8* or *Tead2* was generated using the pAdMax Adenovirus Vector System (Vector Gene Technology Company Ltd.) according to the manufacturer’s protocol. In some experiments, NRCMs were treated with various doses of isobutyrate (pH-adjusted to 7.2; Aladdin, 79-31-2) and valine (Beyotime, ST1502). The details for the reagents used in this study were listed in Supplementary Table 2.

### Isolation of cardiomyocytes and non-cardiomyocytes

The isolation of adult mouse cardiomyocytes and non-cardiomyocytes was performed according to the published method^55^. Briefly, cardiomyocytes were obtained through four rounds of gravity sedimentation, while non-cardiomyocytes were collected by centrifugation at 300 g for 5 min.

### CCK-8 assay

Cell viability was measured by CCK-8 (Beyotime, C0038). NRCMs were plated on 96-well plates at a density of 2 × 104 cells for 24 h and then treated with isobutyrate for 36 h. Subsequently, 10 μl of CCK-8 solution was added to each well and incubated for 4 h, followed by measuring the absorbance at 450 nm.

### qRT-PCR

Total RNA was isolated with TRIzol reagent (Invitrogen, 15596018CN), reverse transcribed, and subjected to real-time qPCR, and target gene expression was quantified with a bidirectional fluorescence quantitative PCR instrument. The β-actin (*Actb*) gene was used as a housekeeping gene to standardise the expression of the target genes. The primer sequences are listed in Supplementary Table 3.

### RNA-seq analysis

Total RNA was extracted with TRIzol reagent. Sequencing was performed on a DNBSEQ platform (DNBSEQ-T7) by Igenecode (Beijing, China). Clean reads were compared to the reference genome with HISAT, and differential expression analysis was performed with DESeq2. Genes with adjusted P values less than 0.05 were considered to be differentially expressed genes. Functional annotation and pathway enrichment analyses were conducted using the ToppGene Suite (http://toppgene.cchmc.org). The upstream transcription factors that regulate the expression of the upregulated genes were predicted with the web tools TRANSFAC and JASPAR PWMs (http://maayanlab.cloud/Enrichr). Genes with fold change > 1.2 and adjusted P value < 0.05 were considered for analysis. The TEAD2 position weight matrix (PWM) logo plot was obtained from the JASPAR database.

### LC‒MS/MS analysis

The H650 high-throughput targeted metabolomics analysis was performed with the help from Applied Protein Technology Co., Ltd. Frozen heart tissues were slowly thawed at 4 °C, and 50 mg of sample was added to a precooled methanol/acetonitrile/water solution (2:2:1, v/v). Then, the sample was homogenised by vortexing, sonicated at 4 °C for 30 min, and then centrifuged at 14,000 × g at 4 °C for 20 min. The supernatant was vacuum dried, redissolved in 100 μL of an acetonitrile/water (acetonitrile:water=1:1, v/v) solvent, and centrifuged at 14,000 × g for 15 min at 4 °C. Finally, the supernatant of the sample was collected.

LC-MS/MS analysis was conducted using a QTRAP MS (AB 6500 + , AB Sciex) coupled to a UHPLC system (1290 Infinity LC, Agilent Technologies) at Shanghai Applied Protein Technology Co., Ltd. Separation was performed on a HILIC column (Waters UPLC BEH Amide, 2.1 × 100 mm, 1.7 μm) and a C18 column (Waters UPLC BEH C18, 2.1 × 100 mm, 1.7 μm), with flow rates of 300 μL/min for HILIC and 400 μL/min for RPLC. QC samples are inserted into the sample queue to monitor and evaluate the stability of the system and the reliability of the experimental data. Mass spectrometry analysis was performed on an AB 6500 + QTRAP mass spectrometer (AB SCIEX). Using MultiQuant or Analyst software to extract the peaks from the raw MRM data, the ratio of the peak area of each substance to the peak area of the internal standard was determined, and the content was calculated according to a standard curve.

Significance was determined using an unpaired Student’s t test. *p* < 0.05 was considered to indicate a statistically significant difference. KEGG pathway enrichment analysis was performed based on the KEGG pathway database (http://www.genome.jp/kegg/pathway.html), and the results were analysed by Fisher’s exact test to calculate the significance level of metabolite enrichment for each pathway.

### Measurement of short-chain acyl-CoA and acylcarnitine levels

Short-chain acyl-CoA levels were detected and analysed as described previously^18,56^, with the help from Centre Testing International Group Co., Ltd. Heart tissues or cultured cardiomyocytes were washed 3 times with cold deionized water, quenched with 1 mL of acetonitrile/isopropanol/water (9:3:4, v/v/v), and scratched after the addition of 200 μL of acetic acid and 50 μL of internal standards. Cell lysates were collected and centrifuged at 4 °C and 16000 × g for 15 min. The supernatants were subsequently purified. LC-MS/MS analysis was performed on an AB Sciex 5500+ triple quadrupole mass spectrometer equipped with an electrospray ionisation (ESI) source. Raw data acquisition and quantitative analysis were conducted using Analyst software version 1.7.2 (AB Sciex). Raw instrument data files were imported for automated peak integration using the IntelliQuant algorithm. Integration parameters: retention time window ±0.3 min, baseline smoothing points 7, peak width range 0.1–0.5 min. The sample content is calculated and output from the quantitative standard curve, with normalisation to internal standards.

### Western blotting analysis

Proteins were extracted with RIPA lysis buffer (Beyotime, P0013B) supplemented with a protease inhibitor cocktail (Beyotime, P1011). Twenty micrograms of proteins were separated by SDS‒PAGE and then transferred to PVDF membranes (Millipore, IEVH00005). After being blocked with TBST supplemented with 5% skim milk, the membranes were incubated with primary antibodies at 4 °C overnight. The membranes were subsequently incubated with horseradish peroxidase-conjugated secondary antibodies (ZSGB-BIO; #ZB2301 and #ZB2305) and exposed to SuperSignal™ West Femto Maximum Sensitivity Substrate (Thermo, 34096) to visualise protein levels. Kibu levels were detected using antibodies from PTM Biolab, either the commercial H3K9(iso)bu (PTM biolab, PTM-305), H3K23(iso)bu (PTM biolab, PTM-307) and pan-K(iso)bu (PTM biolab, PTM-301) antibodies because isobutyryl-CoA constitutes the majority of cellular (iso)butyryl-CoA^15^, or the custom-made H3K9ibu and H3K23ibu antibodies. All antibodies’ details are listed in Supplementary Table 4. The uncropped and unprocessed blots are provided in Source Data file.

### CUT&Tag and qRT-PCR

CUT&Tag was performed according to the manufacturer’s instructions (Novoprotein, N259-YH01). NRCMs were digested using 0.25% trypsin solution, and 200,000 cells were treated with a high concentration of formaldehyde for cross-linking and then subjected to cell permeabilization. Cells were immobilised on the surface of CoA magnetic beads and then sequentially subjected to primary antibody (Anti-FLAG, Cell Signalling Technology, 14793; H3K9(iso)bu, Biolab, PTM-305 and H3K23(iso)bu, Biolab, PTM-307), secondary antibody (Goat Anti-Rabbit IgG H&L, Novoprotein, N269; Goat Anti-Mouse IgG H&L, Novoprotein, N270), transposome incubation and fragmentation. The reaction products were amplified by DNA extraction and verified by qRT-PCR, and the primers used are listed in Supplementary Table 5.

### ATAC-seq analysis

ATAC-seq was performed according to the manufacturer’s instructions (Vazyme, TD711). Sequencing was conducted with the Illumina NovaSeq X Plus platform. The raw data were processed with FASTP to remove adaptors and filter out low-quality and short reads (average mass fraction lower than Q20). The reference genome (mRatBN7.2) index was created by Bowtie2, and then, the filtered reads were mapped to the reference genome. Finally, the peaks were visualised using the Integrative Genomics Viewer (IGV) software.

### Transmission electron microscopy

Heart tissues were fixed using a commercial electron microscope fixative (Servicebio, G1102). Fresh tissue samples were cut into 1 mm³ blocks, post-fixed with 1% osmium tetroxide, dehydrated through graded alcohols and acetone, and embedded in epoxy resin. Ultrathin sections (60–80 nm) were stained with uranyl acetate and lead citrate. Mitochondrial ultrastructure was examined by transmission electron microscopy (TEM) (Hitachi, Servicebio), and mitochondrial length and mitochondrial density were quantitatively analysed using ImageJ software.

### Immunofluorescence staining

Cultured NRCMs were fixed with 4% paraformaldehyde for 15 min, washed with PBS, permeabilized with 0.5% Triton X-100 for 10 min, and blocked with 3% BSA solution for 30 min. Then, the cells were incubated with an anti-α-actinin (Sigma, #A7811; 1:200) antibody at 4 °C overnight. The next day, the cells were washed and stained with the Cross-Adsorbed Secondary Antibody, Alexa Fluor™ 594 (Invitrogen, A-11005) and Hoechst 33342 (Aladdin, H288601). Images were captured with an Inverted Microscope (Leica DMi8), and the cell surface area was measured using ImageJ software. To assess ROS levels in cardiac tissues 4 weeks after TAC or Sham surgery, cryosections of myocardial tissue were prepared and stained with the superoxide-specific fluorescent probe MitoSOX™ Red (Invitrogen, M36008) for 20 min at 37 °C. For TUNEL apoptosis assay, paraffin-embedded cardiac tissue sections were stained using a TUNEL detection kit (Servicebio, G1502) according to the manufacturer’s protocol.

### Statistical analysis

The count data in the Gene Expression Omnibus (GEO) database were normalised and converted using the *vst* function of the DESeq2 R package. Gene set variation analysis (GSVA) was used to calculate the enrichment score of the valine catabolic gene set in the GEO database, and the Pearson correlation of this score with *NPPA* and *NPPB* expression levels was analysed with GraphPad Prism 9. Single-cell RNA sequencing data from the GSE120064 dataset were normalised with the LogNormalize and ScaleData functions of the Seurat package. The FindNeighbors and FindClusters functions were used to cluster the cells. To visualise the clustering results, t-SNE was run for further dimensionality reduction. The addModuleScore function in Seurat was used to calculate the enrichment score of the valine catabolic gene set. The valine catabolic gene set was customised to contain *BCAT2*, *BCKDHA*, *BCKDHB*, *ACAD8*, *HADHA*, *HIBCH*, *HIBADH*, *DBT*, *ECHS1*, *HADH*, *ALDH2*, and *ALDH9A1*.

All the measurement results are expressed as the means ± SDs. Two groups of normally distributed data were statistically analysed using the unpaired Student’s *t* test for equal variance or the Welch *t* test for unequal variance. Two groups of data that did not conform to a normal distribution were analysed using the nonparametric Mann‒Whitney test. Comparisons between multiple groups were performed using one-way analysis of variance (ANOVA) or two-way ANOVA combined with the Bonferroni *post hoc* correction when the data were normally distributed. Otherwise, we used the nonparametric Kruskal–Wallis test followed by Dunn’s *post hoc* test. *P* < 0.05 was considered to indicate a significant difference.

### Reporting summary

Further information on research design is available in the Nature Portfolio Reporting Summary linked to this article.

## Supplementary information

Supplementary Information

Reporting summary

Transparent Peer Review file

## Source data

Source data

## Data Availability

All data are available in the manuscript or the supplementary materials. The bulk RNA seq data is deposited in the NCBI Sequence Read Archive (SRA) under the BioProject accession number PRJNA1448180. The ATAC-seq data are deposited in the SRA under the BioProject accession number PRJNA1448194. The targeted metabolomics data presented in this article have been deposited in MetaboLights under the dataset identifier MTBLS14207. Source data are provided with this paper. Any additional raw data can be obtained by addressing the corresponding author. Source data are provided with this paper.
