# Supplementary material for: ACAD8 deficiency promotes pathological cardiac hypertrophy in response to pressure overload by regulating histone isobutyrylation
Source: Nat Commun. 2026 May 11;17:6298. doi: 10.1038/s41467-026-72949-w (PMC13376936; doi:10.1038/s41467-026-72949-w)
Supplement: Supplementary file 1 — Supplementary Information [file 41467_2026_72949_MOESM1_ESM.pdf]

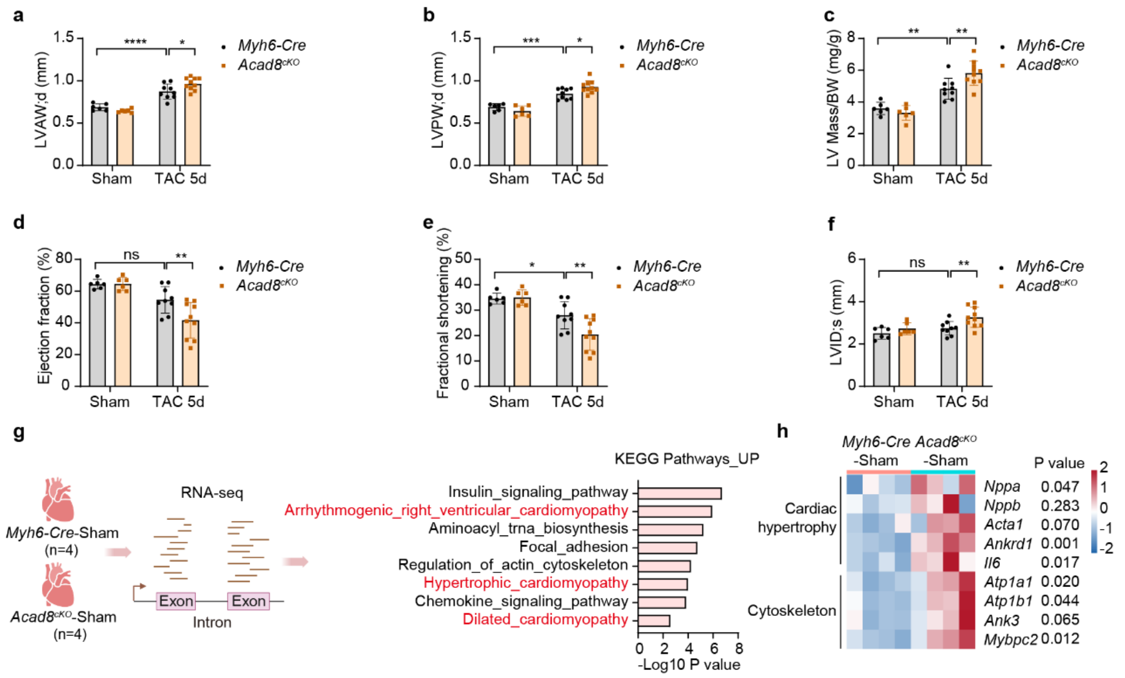

Supplementary Figure 1. *Acad8* deficiency promotes cardiac hypertrophy in mice.

a-c, Diastolic left ventricular anterior wall thickness (LVAW;d) (**a**), Diastolic left ventricular posterior wall thickness (LVPW;d) (**b**) and Left ventricular mass/body weight (LV Mass/BW) (**c**) of *Myh6-Cre* and $Acad8^{cKO}$ mice 5 days after Sham or TAC surgery (n=6~10; *p<0.05, **p<0.01). From left to right: ****P < 0.0001, *P =0.0121, ***P =0.0001, *P =0.0196, **P =0.0017, **P =0.0039, respectively, by two-way ANOVA with Bonferroni *post hoc* correction. n = 6 (*Myh6-Cre*-Sham), 6 ($Acad8^{cKO}$-Sham), 9 (*Myh6-Cre*-TAC), 10 ($Acad8^{cKO}$-TAC) mice/group. **d-f,** Ejection fraction (**d**)**,** fractional shortening (**e**) and systolic left ventricular internal dimension (LVID;s) (**f**) of *Myh6-Cre* and $Acad8^{cKO}$ mice 5 days after Sham or TAC surgery. From left to right: P =0.0643, **P =0.0041, *P =0.0335, **P =0.0046, P =0.4625, **P =0.0097, respectively, ns, not significant, by two-way ANOVA with Bonferroni *post hoc* correction. n = 6 (*Myh6-Cre*-Sham), 6 ($Acad8^{cKO}$-Sham), 9 (*Myh6-Cre*-TAC), 10 ($Acad8^{cKO}$-TAC) mice/group. **g,** Schematic of RNA-seq experimental design for left ventricles of *Myh6-Cre*-Sham and $Acad8^{cKO}$-Sham hearts, with KEGG enrichment analysis of upregulated genes. Created in BioRender. Lab, 5. (2026) https://biorender.com/qnscozz. (Bulk RNA-seq was performed four weeks post sham surgery). **h,** Heatmaps of the relative expression of genes associated with cardiac hypertrophy and cytoskeleton in *Myh6-Cre*-Sham and $Acad8^{cKO}$-Sham hearts based on transcriptomic sequencing data, by two-tailed unpaired Student's t-test. Data are presented as mean values ± SD. Source data are provided as a Source Data file.

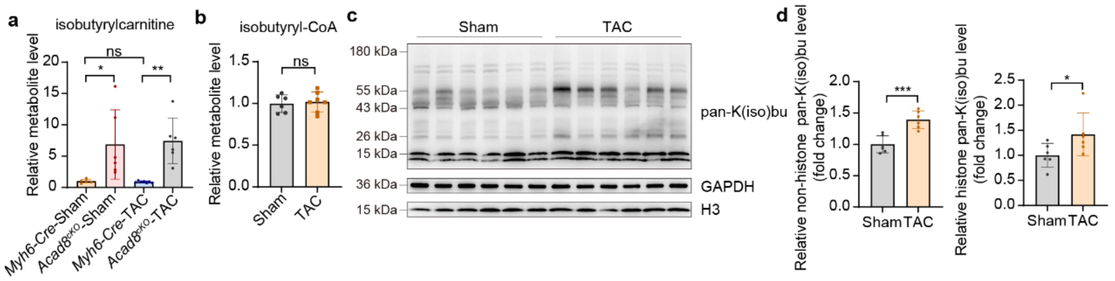

Supplementary Figure 2. Global protein isobutyrylation modification increases in TAC-operated mouse hearts.

a, Quantification of isobutyrylcarnitine levels in *Myh6-Cre* and *Acad8*^{cKO} mice 4 weeks after Sham or TAC surgery. *P=0.0248, P>0.9999, **P=0.0059, ns, not significant, by one-way ANOVA with Bonferroni *post hoc* correction. n = 5 (*Myh6-Cre*-Sham), 6 (*Acad8*^{cKO}-Sham), 7 (*Myh6-Cre*-TAC), 6 (*Acad8*^{cKO}-TAC) mice/group. **b,** Quantification of isobutyryl-CoA levels in Sham-operated and TAC-induced hypertrophic hearts for 4 weeks. P=0.7583, ns, not significant, by two-tailed unpaired Student's *t*-test. n = 6 (Sham), 7 (TAC) mice/group. **c,** Western blotting with GAPDH serves as a loading control and **d,** Quantification of non-histone and histone K(iso)bu levels in Sham-operated and TAC-induced hypertrophic hearts for 4 weeks. ***P=0.0003, *P=0.0317, by one-tailed unpaired Student's *t*-test. n = 6 mice/group. Data are presented as mean values ± SD. Source data are provided as a Source Data file.

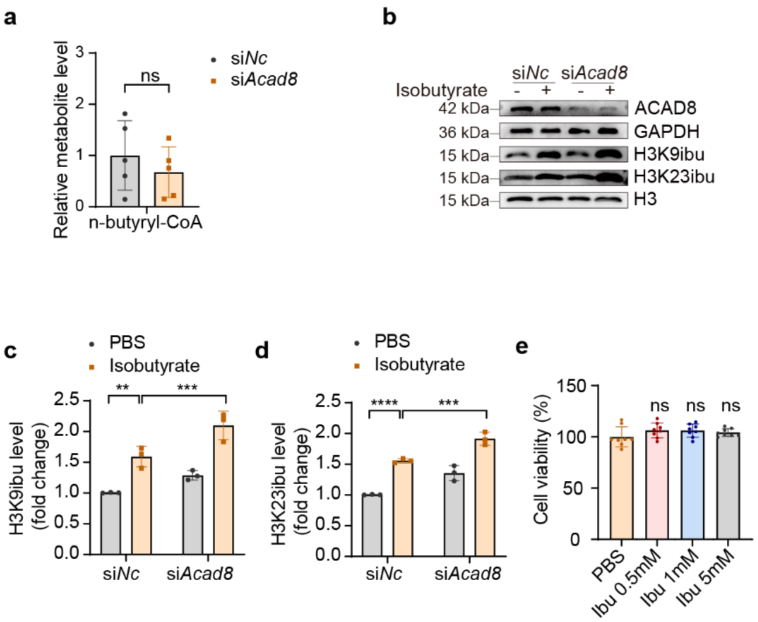

Supplementary Figure 3. Knockdown of *Acad8* in NRCMs enhances isobutyrate-induced histone H3 isobutyrylation modification.

a, Quantification of n-butyryl-CoA levels in NRCMs transfected with si*Nc* or si*Acad8* for 48 hours. *P*=0.4113, ns, not significant, by two-tailed unpaired Student's *t*-test. n = 5 mice/group. **b-d,** Western blotting with GAPDH serves as a loading control and quantification of H3K9ibu and H3K23ibu levels in NRCMs treated with PBS or isobutyrate (5 mM) for 36 hours after transfection with si*Nc* or si*Acad8* for 36 hours. From left to right: ***P* = 0.0013, ****P* = 0.0003, *****P* < 0.0001, ****P* = 0.0002, respectively, by two-way ANOVA with Bonferroni *post hoc* correction. n = 3 biologically independent samples. **e,** CCK-8 assay was performed on NRCMs treated with PBS or isobutyrate at different concentrations for 36 hours. From left to right: *P*=0.2691, *P*=0.2948, *P*=0.7348, respectively, ns, not significant, compared with PBS, by one-way ANOVA with Bonferroni *post hoc* correction. n = 8 biologically independent samples. Data are presented as mean values ± SD. Source data are provided as a Source Data file.

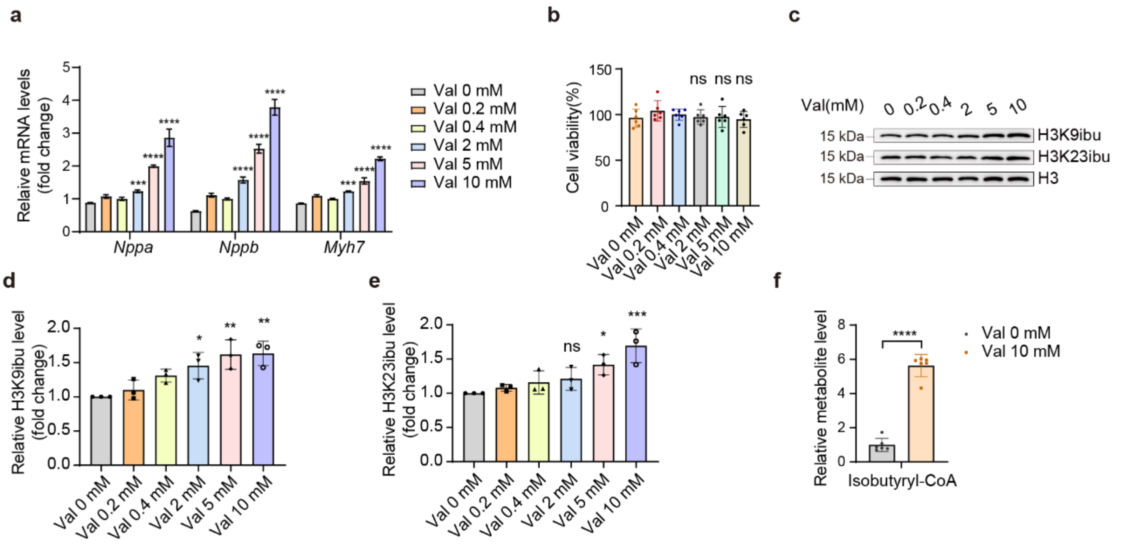

Supplementary Figure 4. Valine treatment elevates intracellular isobutyryl-CoA levels and promotes cardiomyocyte hypertrophy.

a, NRCMs were treated with media containing different concentrations of valine for 36 hours. qRT-PCR was performed to analyse the mRNA levels of hypertrophic genes. From left to right: ***$P = 0.0006$, ****$P < 0.0001$, ****$P < 0.0001$, ****$P < 0.0001$, ****$P < 0.0001$, ****$P < 0.0001$, ***$P = 0.0004$, ****$P < 0.0001$, ****$P < 0.0001$, respectively, compared with 0mM, by two-way ANOVA with Bonferroni *post hoc* correction. n = 3 biologically independent samples. **b,** CCK-8 assay was performed on NRCMs treated with media containing different concentrations of valine for 36 hours. $P > 0.9999$, ns, not significant, compared with 0mM, by one-way ANOVA with Bonferroni *post hoc* correction. n = 6 biologically independent samples. **c,** Western blotting with GAPDH serves as a loading control and **d-e,** Qualification of H3K9ibu and H3K23ibu levels in NRCMs treated with media containing different concentrations of valine for 36 hours. From left to right: *$P = 0.0187$, **$P = 0.0019$, **$P = 0.0015$, $P = 0.5945$, *$P = 0.0299$, ***$P = 0.0007$, respectively, ns, not significant, compared with 0mM, by two-way ANOVA with Bonferroni *post hoc* correction. n = 3 biologically independent samples. **f,** Quantification of isobutyryl-CoA levels in NRCMs treated with media containing different concentrations of valine for 36 hours. ****$P < 0.0001$, by two-tailed unpaired Student's *t*-test. n = 5 biologically independent samples. Data are presented as mean values ± SD. Source data are provided as a Source Data file.

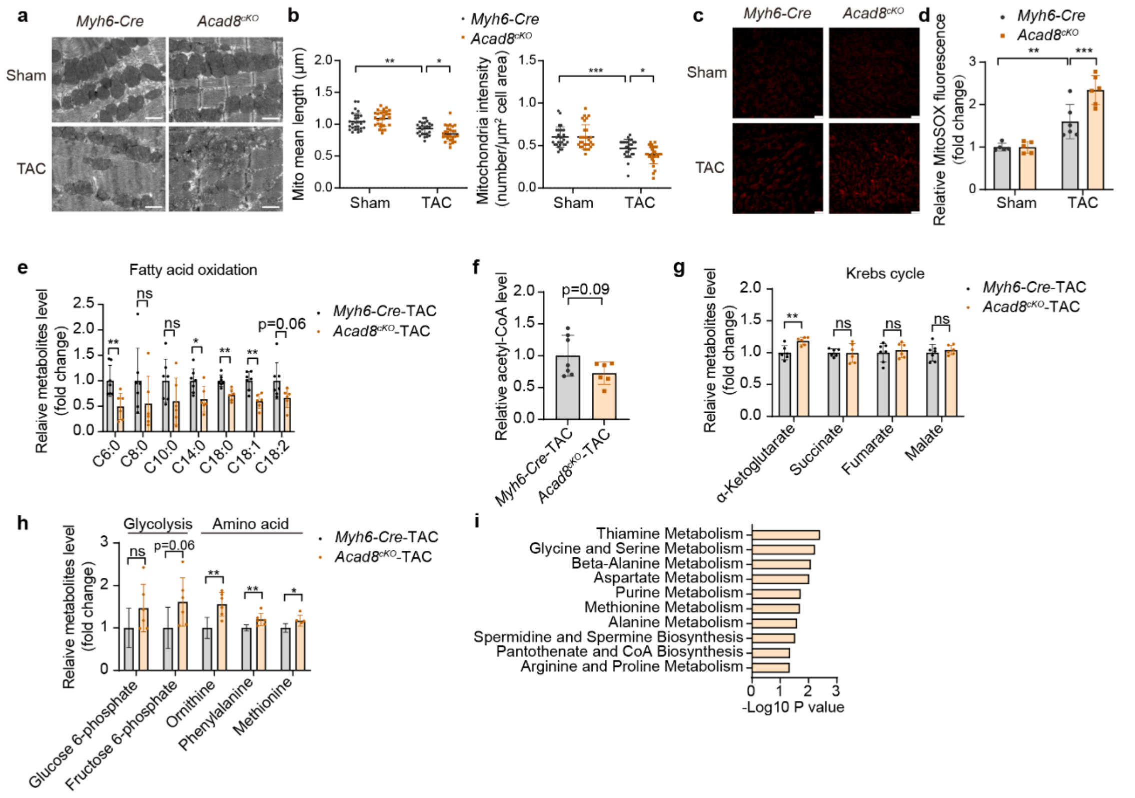

Supplementary Figure 5. *Acad8* deficiency exacerbates mitochondrial structural and functional damage in TAC-operated hearts.

a, Representative electron microscope images of cardiomyocyte mitochondria in *Myh6-Cre* and *Acad8*^{cKO} mice 4 weeks after Sham or TAC surgery. Scale Bar =1μm. **b,** Quantification of mitochondrial mean length and mitochondrial density in heart tissues. From left to right: $^{**}P = 0.0016$, $^{*}P = 0.0254$, $^{***}P = 0.0002$, $^{*}P = 0.0466$, respectively, by two-way ANOVA with Bonferroni *post hoc* correction. n = 3 mice/group, 8–10 random fields were analyzed per sample. **c-d,** Representative image and quantification of relative mitochondrial ROS (MitoSOX Red fluorescence). Scale Bar = 30 μm. $^{**}P = 0.006$, $^{***}P = 0.0006$, by two-way ANOVA with Bonferroni *post hoc* correction. n = 5 (*Myh6-Cre*-Sham), 5 (*Acad8*^{cKO}-Sham), 6 (*Myh6-Cre*-TAC), 6 (*Acad8*^{cKO}-TAC) mice/group. **e,** Quantification of acylcarnitines in *Myh6-Cre*-TAC and *Acad8*^{cKO}-TAC hearts. From left to right: $^{**}P = 0.0089$, $P = 0.2026$, $P = 0.1287$, $^{*}P = 0.0233$, $^{**}P = 0.001$, $^{**}P = 0.0011$, $P = 0.0629$, respectively, ns, not significant, by two-tailed unpaired Student's *t*-test. n = 7 (*Myh6-Cre*-TAC), 6 (*Acad8*^{cKO}-TAC) mice/group. **f,** Quantification of acetyl-CoA levels in *Myh6-Cre*-TAC and *Acad8*^{cKO}-TAC hearts. $P = 0.0087$, by two-tailed unpaired Student's *t*-test. n = 7 (*Myh6-Cre*-TAC), 6 (*Acad8*^{cKO}-TAC) mice/group. **g,** Quantification of metabolites associated with the Krebs cycle in *Myh6-Cre*-TAC and *Acad8*^{cKO}-TAC hearts. From left to right: $^{**}P = 0.0051$, $P = 0.9248$,

$P = 0.6188$, $P = 0.5111$, respectively, by two-tailed unpaired Student's *t*-test. n = 7 (*Myh6-Cre*-TAC), 6 (*Acad8*^{cKO}-TAC) mice/group. **h,** Quantification of metabolites associated with glycolysis and amino acid in *Myh6-Cre*-TAC and *Acad8*^{cKO}-TAC hearts. From left to right: $P = 0.1272$, $P = 0.0609$, $^{**}P = 0.003$, $^{**}P = 0.0091$, $^{*}P = 0.0209$, respectively, ns, not significant, by two-tailed unpaired Student's *t*-test. n = 7 (*Myh6-Cre*-TAC), 6 (*Acad8*^{cKO}-TAC) mice/group. **i,** Pathways significantly enriched for differential metabolites (targeted metabolomic analysis) in *Acad8*^{cKO}-TAC hearts compared with *Myh6-Cre*-TAC hearts. Data are presented as mean values ± SD. Source data are provided as a Source Data file.

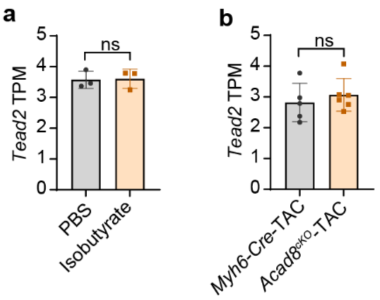

Supplementary Figure 6. Isobutyrate treatment and *Acad8* deficiency do not alter *Tead2* expression levels.

a, *Tead2* expression in NRCMs treated with PBS or isobutyrate fromo RNA-seq data. $P = 0.9007$, ns, not significant, by two-tailed unpaired Student's t-test. n = 3 biologically independent samples. **b,** *Tead2* expression in *Myh6*-Cre-TAC and $Acad8^{cKO}$-TAC hearts. RNA-seq data was performed 4 weeks post TAC surgery. $P = 0.4940$, ns, not significant, by two-tailed unpaired Student's t-test. n = 5 (*Myh6-Cre*-TAC), 6 ($Acad8^{cKO}$-TAC) mice/group. Data are presented as mean values ± SD. Source data are provided as a Source Data file.

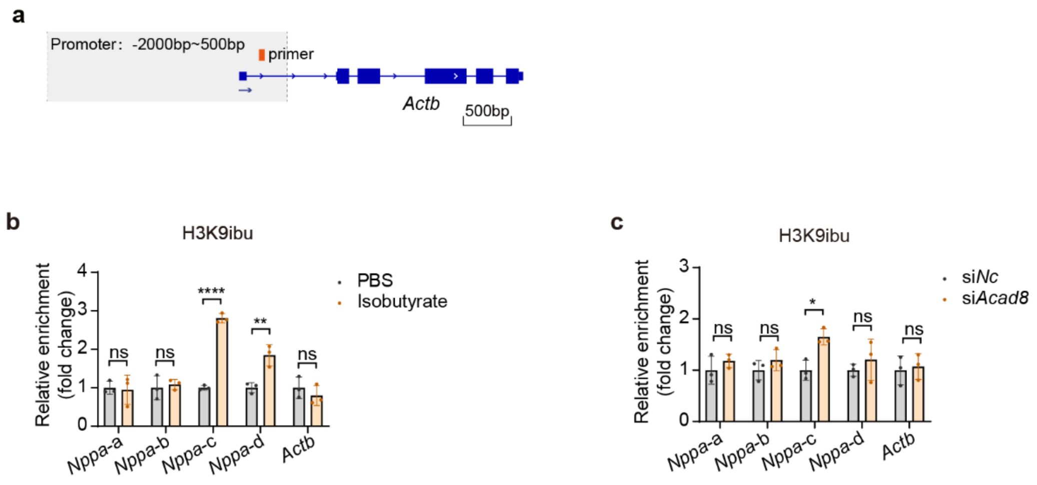

Supplementary Figure 7. Isobutyrate treatment and *Acad8* deficiency affect the enrichment of H3K9ibu at the promoter region of *Nppa*.

a, Schematic showing the *Actb* gene locus, showing the qPCR primer-binding region (238 bp–318 bp) used as an internal reference. **b,** CUT&Tag and qRT-PCR analysis for H3K9ibu enrichment at *Nppa* promoter regions after isobutyrate treatment. From left to right: $P > 0.9999$, $P > 0.9999$, ****$P < 0.0001$, **$P = 0.0013$, $P > 0.9999$, ns, not significant, by two-way ANOVA with Bonferroni *post hoc* correction. n = 3 biologically independent samples. **c,** CUT&Tag and qRT-PCR analysis for H3K9ibu enrichment at *Nppa* promoter regions after *Acad8* knockdown. From left to right: $P > 0.9999$, $P > 0.9999$, *$P = 0.014$, $P > 0.9999$, $P > 0.9999$, ns, not significant, by two-way ANOVA with Bonferroni *post hoc* correction. n = 3 biologically independent samples. The information for qRT-PCR primer design is shown in Figure 4m. Data are presented as mean values ± SD. Source data are provided as a Source Data file.

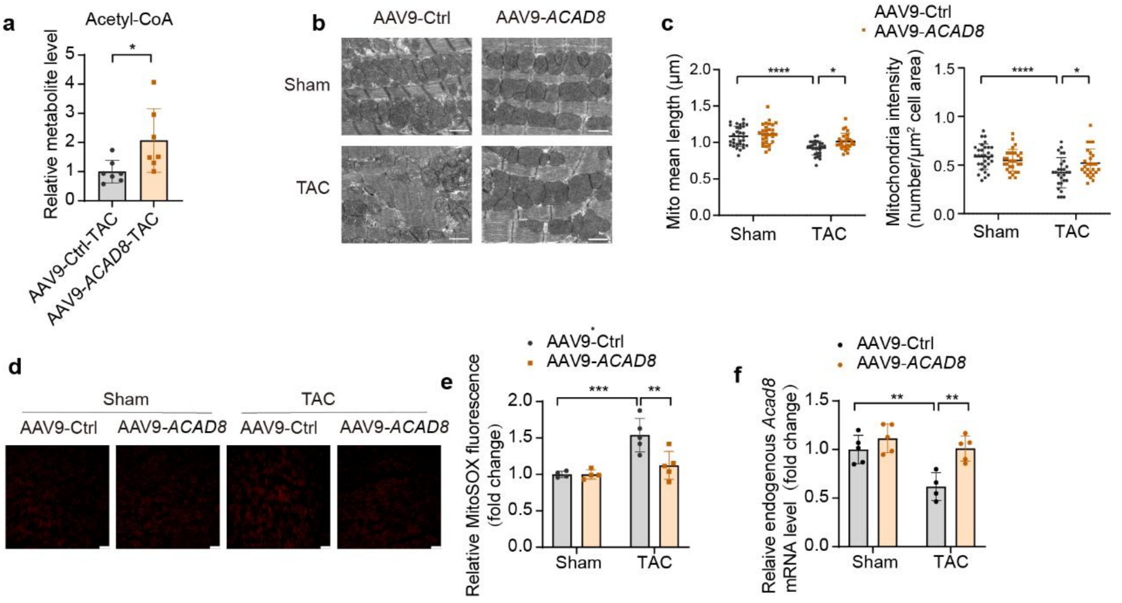

Supplementary Figure 8. AAV9-mediated ACAD8-OE in cardiomyocytes ameliorates TAC-induced cell death and mitochondrial damage.

a, Quantification of acetyl-CoA levels in *Myh6-Cre*-TAC and *Acad8*^{cKO}-TAC hearts. **P* =0.0307, by two-tailed unpaired Student's *t*-test. n = 7 mice/group. **b,** Representative electron microscope images of cardiomyocyte mitochondria in the hearts of mice infected with AAV9-cTnT-Ctrl or AAV9-cTnT-*ACAD8* 4 weeks after Sham or TAC surgery. Scale Bar =1μm. **c,** Quantification of mitochondrial mean length and mitochondrial density in heart tissues. From left to right: *****P* < 0.0001, **P* = 0.0188, *****P* = < 0.0001, **P* = 0.0178, respectively, by two-way ANOVA with Bonferroni *post hoc* correction. n = 3 mice/group, 8–10 random fields were analyzed per sample. **d,** Representative image and **e,** quantification of relative mitochondrial ROS (MitoSOX Red fluorescence) in the hearts of mice infected with AAV9-cTnT-Ctrl or AAV9-cTnT-*ACAD8* 4 weeks after Sham or TAC surgery. Scale Bar = 30 μm. ****P* = 0.0005, ***P* = 0.003, by two-way ANOVA with Bonferroni *post hoc* correction. n = 4 (AAV9-cTnT-Ctrl-Sham), 4 (AAV9-cTnT-*ACAD8*-Sham), 5 (AAV9-cTnT-Ctrl-TAC), 5 (AAV9-cTnT-*ACAD8*-TAC) mice/group. **f,** qRT–PCR was performed to analyse the mRNA expression level of endogenous *Acad8* in the hearts of mice infected with AAV9-cTnT-Ctrl or AAV9-cTnT-*ACAD8* 4 weeks after Sham or TAC surgery.***P* =0.0022, ***P* =0.0017, by two-way ANOVA with Bonferroni *post hoc* correction. n = 5 (AAV9-cTnT-Ctrl-Sham), 5 (AAV9-cTnT-*ACAD8*-Sham), 4 (AAV9-cTnT-Ctrl-TAC), 5 (AAV9-cTnT-*ACAD8*-TAC) mice/group. Data are presented as mean values ± SD. Source data are provided as a Source Data file.

Supplementary Table 1. Clinical characterization of patient samples used in this study.

Name	Gender	SAM	Mitral regurgitation	Atrial fibrillation	NYHA class (I, II, III, IV)	β-blocker therapy
NC-1#	F	N/A	N/A	N/A	N/A	N/A
NC-2#	F	N/A	N/A	N/A	N/A	N/A
NC-3#	M	N/A	N/A	N/A	N/A	N/A
NC-4#	M	N/A	N/A	N/A	N/A	N/A
HCM-1#	M	Y	Y	N	III	Y
HCM-2#	M	Y	Y	N	II	N
HCM-3#	M	Y	Y	N	III	N
HCM-4#	M	Y	Y	N	II	Y

Supplementary Table 2. The reagents used in this study.

Reagents	Source	Identifier
Isoflurane	RWD	R510-22
Tamoxifen	Servicebio	T9010
DMEM	Servicebio	G4511
Bovine serum	hyclone	SH30070
Penicillin/Streptomycin/Amphotericin B	Solarbio	P7630
5-Bromodeoxyuridine (BrdU)	Sigma	B5002
Lipofectamine RNAiMAX Transfection Reagent	Invitrogen	13778150
Isobutyrate	Aladdin	79-31-2
Valine	Beyotime	ST1502
Trypsin (0.25%)	Leagene	cc0128
Digestive enzymes	Thermo	88281
Paraformaldehyde (4%)	Servicebio	G1101
H&E Stain	Servicebio	G1076
Triton X-100	Sigma	T8787
WGA (Wheat Germ Agglutinin)	Sigma-Aldrich	L4895
Masson Stain	Servicebio	G1006
DAPI or Hoechst 33342	Aladdin	H288601
α-Actinin antibody	Sigma	A7811
Alexa Fluor 594 secondary antibody	Invitrogen	A-11005
BSA (Bovine Serum Albumin)	Sigma	A9418
RIPA lysis buffer	Beyotime	P0013B
Protease inhibitor cocktail	Beyotime	P1011
SuperSignal West Femto Substrate	Thermo	34096
TRIzol Reagent	Invitrogen	15596018CN
Reverse transcription kit	Takara	6215
2X SYBR qPCR Master Mix	Vazyme	Q712-02
ATAC-seq Kit	Vazyme	TD711
CUT&Tag Kit	Novoprotein	N259-YH01
CCK-8	Beyotime	C0038
MitoSOX Red	Invitrogen	M36008
TUNEL Detection Kit	Servicebio	G1502
Protein marker	Thermo	26616

Supplementary Table 3. The primers for qRT-PCR used in this study.

Gene	Primers
mouse *Nppa*-F	5’-TCTTCCTCGTCTTGGCCTTT-3’
mouse *Nppa*-R	5’-CCAGGTGGTCTAGCAGGTTC-3’
mouse *Nppb*-F	5’-TGGGAGGTCACTCCTATCCT-3’
mouse *Nppb*-R	5’-GGCCATTTCCTCCGACTTT-3’
mouse *Acta1*-F	5’-CCCAAAGCTAACCGGGAGAAG-3’
mouse *Acta1*-R	5’-GACAGCACCGCCTGGATAG-3’
mouse *Actb*-F	5’-CATTGCTGACAGGATGCAGAAGG-3’
mouse *Actb*-R	5’-TGCTGGAAGGTGGACAGTGAGG-3’
mouse *Acad8*-F	5’-TGGCGGAGTGGGATCAGAA-3’
mouse *Acad8*-R	5’-CCACATCTGTTCGCACATAGAC-3’
rat *Acad8*-F	5’-CGATCCTTCCTTGGGGCTAA-3’
rat *Acad8*-R	5’-ACAGGGAACAGCTCCTTCTGAT-3’
rat *Nppa*-F	5’-GAAGATGCCGGTAGAAGATGAG-3’
rat *Nppa*-R	5’-AGAGCCCTCAGTTTGCTTTTC-3’
rat *Nppb*-F	5’-GGTGCTGCCCCAGATGATT-3’
rat *Nppb*-R	5’-CTGGAGACTGGCTAGGACTTC-3’
rat *Myh7*-F	5’-GCCCCAAATGCAGCCAT-3’
rat *Myh7*-R	5’-CGCTCAGTCATGGCGGAT-3’
rat *Actb*-F	5’-CAGGGTGTGATGGTGGGTATGG-3’
rat *Actb*-R	5’-AGTTGGTGACAATGCCGTGTTC-3’

Supplementary Table 4. The antibodies used in this study.

Antibodies	Source	Identifier	Dilution
ACAD8	Abcam	ab102810	1:500
BCAT2	Abcam	ab307833	1:1000
BCKDHA	Abcam	ab138460	1:1000
HADHA	Proteintech	10758-1-AP	1:1000
HIBCH	Proteintech	14603-1-AP	1:1000
HIBADH	Proteintech	13466-1-AP	1:1000
H3	Proteintech	17168-1-AP	1:1000
TEAD2	Proteintech	21159-1-AP	1:1000
GAPDH	Proteintech	60004-1-AP	1:1000
H3K9(iso)bu	PTM biolab	PTM-305	1:1000
H3K23(iso)bu	PTM biolab	PTM-307	1:1000
Pan-K(iso)bu	PTM biolab	PTM-301	1:1000
H3K9ibu	PTM biolab	custom-made	1:2000
H3K23ibu	PTM biolab	custom-made	1:2000
HRP-conjugated secondary antibody (anti-rabbit)	ZSGB-BIO	ZB2301	1:5000
HRP-conjugated secondary antibody (anti-mouse)	ZSGB-BIO	ZB2305	1:5000
Goat Anti-Rabbit IgG H&L (secondary)	Novoprotein	N269	1:200
Goat Anti-Mouse IgG H&L (secondary)	Novoprotein	N270	1:200
Anti-α-actinin	Sigma	A7811	1:200
Alexa Fluor 594-conjugated secondary antibody	Invitrogen	A-11005	1:500
Hoechst 33342	Aladdin	H288601	1:1000

Supplementary Table 5. The primers for CUT&Tag and qRT-PCR used in this study.

Gene	Primers
CUT-rat *Nppa*-F	5’-AAGGAATCCTGAGGCGAGC-3’
CUT-rat *Nppa*-R	5’-CCTCAGCTGCAAGAGTCACAT-3’
CUT-rat *Nppb*-F	5’-ACCCACAACGAACACTCCAC-3’
CUT-rat *Nppb*-R	5’-TTCTCATGGAGTAGGGGTGGG-3’
CUT-rat *Gapdh*-F	5’-TGACAACTCCCTCAAGATTGTCA-3’
CUT-rat *Gapdh*-R	5’-GGCATGGACTGTGGTCATGA-3’
Kibu-rat *Nppa* a-region-F	5’-TTCAATCCCCACTCCCTCCT-3’
Kibu-rat *Nppa* a-region-R	5’-CTTTTCCTTCTCGGCCCTGT-3’
Kibu-rat *Nppa* b-region-F	5’-GGGACCACCTCTCGTTTCAC-3’
Kibu-rat *Nppa* b-region-R	5’-GTCTCCCACGACTTGTGTCC-3’
Kibu-rat *Nppa* c-region-F	5’-GGACACAAGTCGTGGGAGAC-3’
Kibu-rat *Nppa* c-region-R	5’-ATGAAGTCAGCCAGGAAGCC-3’
Kibu-rat *Nppa* d-region-F	5’-AAGGAATCCTGAGGCGAGC-3’
Kibu-rat *Nppa* d-region-R	5’-CCTCAGCTGCAAGAGTCACAT-3’
Kibu-rat *Actb*-F	5’-GTTTGCAGTCGTATTCCCGC-3’
Kibu-rat *Actb*-R	5’-CTCTAGTGTGTCCCCAAGCC-3’
