# Supplementary material for: ACAD8 deficiency promotes pathological cardiac hypertrophy in response to pressure overload by regulating histone isobutyrylation
Source: Nat Commun. 2026 May 11;17:6298. doi: 10.1038/s41467-026-72949-w (PMC13376936; doi:10.1038/s41467-026-72949-w)
Supplement: Supplementary file 2 — Reporting summary [file 41467_2026_72949_MOESM2_ESM.pdf]

nature portfolio

Corresponding author(s): Xiaoqiang Tang, Hou-Zao Chen or De-Pei Liu

Last updated by author(s): Apr 4, 2026

Reporting Summary

Nature Portfolio wishes to improve the reproducibility of the work that we publish. This form provides structure for consistency and transparency in reporting. For further information on Nature Portfolio policies, see our Editorial Policies and the Editorial Policy Checklist.

Statistics

For all statistical analyses, confirm that the following items are present in the figure legend, table legend, main text, or Methods section.

n/a	Confirmed	
☐	☒	The exact sample size (*n*) for each experimental group/condition, given as a discrete number and unit of measurement
☐	☒	A statement on whether measurements were taken from distinct samples or whether the same sample was measured repeatedly
☐	☒	The statistical test(s) used AND whether they are one- or two-sided<br>*Only common tests should be described solely by name; describe more complex techniques in the Methods section.*
☒	☐	A description of all covariates tested
☐	☒	A description of any assumptions or corrections, such as tests of normality and adjustment for multiple comparisons
☐	☒	A full description of the statistical parameters including central tendency (e.g. means) or other basic estimates (e.g. regression coefficient) AND variation (e.g. standard deviation) or associated estimates of uncertainty (e.g. confidence intervals)
☐	☒	For null hypothesis testing, the test statistic (e.g. *F*, *t*, *r*) with confidence intervals, effect sizes, degrees of freedom and *P* value noted<br>*Give P values as exact values whenever suitable.*
☒	☐	For Bayesian analysis, information on the choice of priors and Markov chain Monte Carlo settings
☒	☐	For hierarchical and complex designs, identification of the appropriate level for tests and full reporting of outcomes
☐	☒	Estimates of effect sizes (e.g. Cohen's *d*, Pearson's *r*), indicating how they were calculated

Our web collection on statistics for biologists contains articles on many of the points above.

Software and code

Policy information about availability of computer code

Data collection	No custom code was used to collect data. Data was collected using proprietary software for specific experiments.Western Blot analysis was performed using Bio-Rad. Immunofluorescence images were captured using fluorescence microscope (Leica).
Data analysis	GraphPad Prism version 9.0.0, R version 4.4 (DESeq2, GSVA, Seurat packages), ImageJ version 1.52i

For manuscripts utilizing custom algorithms or software that are central to the research but not yet described in published literature, software must be made available to editors and reviewers. We strongly encourage code deposition in a community repository (e.g. GitHub). See the Nature Portfolio guidelines for submitting code & software for further information.

Data

Policy information about availability of data

All manuscripts must include a data availability statement. This statement should provide the following information, where applicable:

- Accession codes, unique identifiers, or web links for publicly available datasets
- A description of any restrictions on data availability
- For clinical datasets or third party data, please ensure that the statement adheres to our policy

Provide your data availability statement here.

Research involving human participants, their data, or biological material

Policy information about studies with human participants or human data. See also policy information about sex, gender (identity/presentation), and sexual orientation and race, ethnicity and racism.

Reporting on sex and gender	sex information has been obtained, but sex was not considered as a biological variable in this study.
Reporting on race, ethnicity, or other socially relevant groupings	Not collected.
Population characteristics	Human cardiac tissue samples were obtained from patients of Tongji Hospital, Tongji Medical College. Samples were obtained from 4 patients with HCM and 4 patients without HCM.
Recruitment	Hypertrophic cardiac tissue samples were obtained from patients who underwent surgical resection due to a prior diagnosis of HCM. Control cardiac tissue samples were obtained from normal heart donors. The sex and age were not considered in this analysis.
Ethics oversight	The samples were obtained with informed consent and with the approval of the institutional review boards (Human Research Ethics Committees of Tongji Hospital, Tongji Medical College, Huazhong University of Science and Technology, Wuhan, China).

Note that full information on the approval of the study protocol must also be provided in the manuscript.

Field-specific reporting

Please select the one below that is the best fit for your research. If you are not sure, read the appropriate sections before making your selection.

☒ Life sciences ☐ Behavioural & social sciences ☐ Ecological, evolutionary & environmental sciences

For a reference copy of the document with all sections, see nature.com/documents/nr-reporting-summary-flat.pdf

Life sciences study design

All studies must disclose on these points even when the disclosure is negative.

Sample size	Sample sizes were based on similar published studies. For in vivo studies, at least 3 mice were used for each condition. The number of the independent replicates is indicated in each figure. At least 3 experimental replicates were used unless otherwise noted.
Data exclusions	None.
Replication	All in vivo and in vitro experiments were independently performed at least 3 times. All attempts at replication were successful.
Randomization	For experiments employing wild-type mice, animals were randomized. For experiments using genetically modified mice, animals were grouped through their genotype. Cells were grown under the same conditions and randomly allocated indifferent groups without bias.
Blinding	The investigators were blinded to group allocation during data collection and analysis.

Reporting for specific materials, systems and methods

We require information from authors about some types of materials, experimental systems and methods used in many studies. Here, indicate whether each material, system or method listed is relevant to your study. If you are not sure if a list item applies to your research, read the appropriate section before selecting a response.

Materials & experimental systems

n/a	Involved in the study	
☐	☒	Antibodies
☒	☐	Eukaryotic cell lines
☒	☐	Palaeontology and archaeology
☐	☒	Animals and other organisms
☒	☐	Clinical data
☒	☐	Dual use research of concern
☒	☐	Plants

Methods

n/a	Involved in the study	
☒	☐	ChIP-seq
☒	☐	Flow cytometry
☒	☐	MRI-based neuroimaging

Antibodies

Antibodies used	Anti-ACAD8, abcam, ab102810, 1:500 dilution;<br>Anti-BCAT2, abcam, ab307833, 1:1000 dilution;<br>Anti-BCKDHA, abcam, ab138460, 1:1000 dilution;<br>Anti-HADHA, Proteintech, 10758-1-AP, 1:1000 dilution;<br>Anti-HIBCH, Proteintech, 14603-1-AP, 1:1000 dilution;<br>Anti-HIBADH, Proteintech, 13466-1-AP, 1:1000 dilution;<br>Anti-FLAG, Cell Signaling Technology, 14793, 1:50 dilution;<br>Anti-GAPDH, Proteintech, 60004-1-Ig, 1:20000 dilution;<br>Anti-H3K9(iso)Bu, PTMBio, PTM-305, 1:1000 dilution;<br>Anti-H3K27(iso)Bu, PTMBio, PTM-307, 1:1000 dilution;<br>Anti-Histone-H3, Proteintech, 17168-1-AP, 1:5000 dilution;<br>Anti-TEAD2, Proteintech, 21159-1-AP, 1:1000 dilution;<br>Anti-pan-K(iso)Bu, PTMBio, PTM-301, 1:1000 dilution;<br>Anti-H3K9iBu, PTMBio, custom-made, 1:2000 dilution;<br>Anti-H3K27iBu, PTMBio, custom-made, 1:2000 dilution;<br>Anti-α-actinin, Sigma, A7811,1:200 dilution;<br>Alexa Fluor 594-conjugated secondary antibody, Invitrogen, A-11005,1:500 dilution;<br>HRP-conjugated secondary antibody (anti-rabbit), ZSGB-BIO, ZB2301,1:5000 dilution;<br>AHRP-conjugated secondary antibody (anti-mouse), ZSGB-BIO, ZB2305,1:5000 dilution;<br>Goat Anti-Rabbit IgG H&L (secondary), N269, 0.01mg/ml,1:200 dilution;<br>Goat Anti-Rabbit IgG H&L (secondary), N270, 0.01mg/ml,1:200 dilution.
Validation	The antibodies were validated by the commercial suppliers from whom they were purchased and have been used in multiple previous studies.

Animals and other research organisms

Policy information about studies involving animals; ARRIVE guidelines recommended for reporting animal research, and Sex and Gender in Research

Laboratory animals	We used male C57BL/6 mice (8 w old) and SD rats(1–3-day-old). The strains included Myh6-Cre(αMHC-MerCreMer), Acad8flox/flox, Acad8flox/flox;αMHC-MerCreMer and C57BL/6 wild type mice.
Wild animals	The study did not involve wild animals.
Reporting on sex	Only male mice were used in this study.
Field-collected samples	The study did not involve samples collected from the field.
Ethics oversight	All the animal protocols were approved by the Animal Care and Use Committee at the Institute of Basic Medical Sciences, Chinese Academy of Medical Sciences, and Peking Union Medical College.

Note that full information on the approval of the study protocol must also be provided in the manuscript.

Plants

Seed stocks	N/A
Novel plant genotypes	N/A
Authentication	N/A
