# Supplementary material for: ACAD8 deficiency promotes pathological cardiac hypertrophy in response to pressure overload by regulating histone isobutyrylation
Source: Nat Commun. 2026 May 11;17:6298. doi: 10.1038/s41467-026-72949-w (PMC13376936; doi:10.1038/s41467-026-72949-w)
Supplement: Supplementary file 3 — Transparent Peer Review file [file 41467_2026_72949_MOESM3_ESM.pdf]

nature portfolio

Peer Review File

ACAD8 deficiency promotes pathological cardiac hypertrophy in response to pressure overload by regulating histone isobutyrylation

Corresponding Author: Professor De-Pei Liu

This manuscript has been previously reviewed at another journal. This document only contains information relating to versions considered at Nature Communications.

This file contains all reviewer reports in order by version, followed by all author rebuttals in order by version.

Version 1:

Reviewer comments:

Reviewer #1

(Remarks to the Author)
In this revised report, authors provided numerous new data to address the main issues raised by the reviewers.
1. In one set of experiment, authors measured isobutyryl-CoA in diseased heart. In both human and mouse- post TAC hearts, the metabolite concentrations were not changed. Although authors argued for a lack of dynamic measurements may have masked the differences, the results nevertheless directly challenged the key provision of this report related to ACAD8 deficiency and accumulation of isobutyryl-CoA to drive epigenetic reprogramming and other defects. The elevated blood level of isobutyrylcarnitine in clinical studies can not be properly extrapolated into cardiac defects in isobutyryl-CoA metabolic defects in hearts. This is particularly relevant since isobutrylcarnitine content was not changed in post-TAC mouse heart in either WT or the KO mice. In short, the physiological relevance of isobutyrul-CoA accumulation is a key element of this report and a central data to support the overall working hypothesis. The results would make this hypothesis highly questionable.
2. It is clear that cardiac tissue level of isobutyrate-CoA is relatively low. It is not clear if 0.5 mM isobutyrate treatment would lead to what levels of isobutyryl-CoA in NRVM relative to intra-cardiac tissue levels observed in vivo. Metabolic impact is heavily dependent on the level of metabolites. Therefore, a clear demonstration of physiologically relevant concentrations of these metabolites would be critical to establish their biological impact.
3. The lack of spontaneous hypertrophy from KO in vivo contradicted the observation made in vitro at phenotypic level. From additional in vivo studies at earlier time points post-TAC, it is also clear that contractile dysfunction manifested in parallel with hypertrophy in the KO mice. Therefore, it remains unclear whether cardiac hypertrophy is the primary outcome of Acad8 inactivation.
4. Adding H3 Kibu modification data is exciting and important. However, changes in the total level was not sufficient to support its biological function associated with targeted gene expression. If authors can demonstrate modified H3K9 and K3K23 directly decorate hypertrophic gene promoters in association with TAC induced hypertrophy, it would be a major finding and a significant step forward in the field.

Reviewer #2

(Remarks to the Author)

The authors show in Supplementary Figure 2 that isobutyrylcarnitine is elevated in Acad8cKO mice at baseline (sham). However TAC fails to increase the level of isobutyrylcarnitine or isobutyryl-CoA in these mice. Obviously the isobutyryl-CoA levels do not correlate with cardiac phenotype. This is puzzling and would require modifications in the conclusion. This reviewer is not convinced by the authors' conclusions. Does the baseline phenotype condition the heart in a way more susceptible to stress?

Since the enhancement of pathological hypertrophy is seen only after pressure overload, the authors should modify the title

of this work
as "ACAD8 deficiency promotes pathological cardiac hypertrophy in response to pressure overload by regulating......."

Version 2:

Reviewer comments:

Reviewer #1

(Remarks to the Author)
In this revision, authors provided several additional analysis to support the validity of their central hypothesis.

1). The missing key data supporting the change of isobutyryl-CoA metabolite in the setting of heart failure remains a source of concern as this is the most novel aspect of this study. Although over-expression offered interesting and potentially important support for its translational relevance.
2). The data supporting the contribution of downstream histone modification as the underlying mechanism is still largely correlative. Authors indeed noted numerous changes at morphological, sub-cellular levels in addition to transcriptomic changes. While they are correlated with lower isobutyrul-CoA levels in both tissue and cells, the Cut-Tag assays were not performed in heart tissue and were not properly controlled for house-keeping genes. It is also not clear if the c-region of the Nppa gene promoter enriched for H3 Kibu was also colocalized with TEAD2 binding activities based on the Cut-Tag assay.
3). In addition to adding Isobutyrate (which can be quite non-specific), authors can also test the hypothesis at least in vitro to add or reduce Valine level in the media, and to determine the direct impact on hypertrophic status, isobutyral-CoA level, histone modification or hypertrophic gene expression.

Reviewer #2

(Remarks to the Author)
No further concerns.

Version 3:

Reviewer comments:

Reviewer #1

(Remarks to the Author)
This revision has incorporated additional data to further clarify and support the central conclusion.

Open Access This Peer Review File is licensed under a Creative Commons Attribution 4.0 International License, which permits use, sharing, adaptation, distribution and reproduction in any medium or format, as long as you give appropriate credit to the original author(s) and the source, provide a link to the Creative Commons license, and indicate if changes were made.

In cases where reviewers are anonymous, credit should be given to 'Anonymous Referee' and the source.
The images or other third party material in this Peer Review File are included in the article's Creative Commons license, unless indicated otherwise in a credit line to the material. If material is not included in the article's Creative Commons license and your intended use is not permitted by statutory regulation or exceeds the permitted use, you will need to obtain permission directly from the copyright holder.
To view a copy of this license, visit https://creativecommons.org/licenses/by/4.0/

Response to Reviewer #1

In this report, valine catabolic activity was investigated in the context of cardiac hypertrophy. Based on several sets of gene expression (individual or gene set), a defect in Valine catabolism was observed, with particular focus on ACAD8. Its function was investigated in vitro and in vivo by loss-of-function approach. Metabolomic analysis and gene expression analysis provided further demonstrations on the metabolic impact of ACAD8 KO on valine catabolism and hypertrophic gene regulation. Furthermore, cardioprotective effect of ACAD8 expression against hypertrophy was also demonstrated in vitro and in vivo.

Demonstrating the functional impact of valine catabolism in pathological hypertrophy is new and interesting. The particular role of ACAD8 in cardiac hypertrophy has not been reported. However, the current study is incomplete and the data supporting this major conclusion is problematic.

Dear reviewer,

Thank you very much for accepting to review our manuscript and for your constructive suggestions. We have performed some additional experiments to address them. We hope that we have understood your concerns and addressed them accurately. The manuscript has been revised accordingly and the major revised parts have been highlighted in yellow color. A point-by-point response to the comments is listed below. The figures shown below are labelled in the order as in the manuscript.

Comment #1-1: Is the effect on cardiac hypertrophy physiological and valine specific? Authors presented in Fig. 1 some targeted gene expression analysis focusing on valine gene set. However, several of the validated genes are also involved in other BCAA catabolic activities, including BCKDHA/B and BCAT, upstream of ACAD8. The metabolomic data shown in Fig. 3 missed important controls of SHAM animals with and without Acad8 KO in order to directly compare surrogate isobutyryl-carnitine levels in wildtype mice after TAC as well as Acad8KO hearts. In addition, the gene expression altered by isobutyrate treatment in cardiomyocytes also showed significant impact on BCAA catabolism, further raising the question on the specificity of the phenotypic impact from Valine catabolism. Finally, authors used 5mM of isobutyrate in cultured cardiomyocytes without clear justification. There are reported cytotoxicities of this compound at high enough concentration, and a dosage response should be performed using physiological levels.

Response: Thank you for your comment. According to your suggestions about

physiological function and valine specificity, we have conducted follow-up experiments and analyses: (1) Re-analysis of valine metabolic gene sets; (2) Additional quantification of isobutyryl-CoA and isobutyryl-carnitine in TAC hearts; (3) Re-analysis of BCAA-related gene expression alterations induced by isobutyrate; (4) Cellular toxicity and dose-response characterization of isobutyrate. To ensure a clear and organized response, we have divided your original comment into several parts based on the different aspects you raised. Below are our detailed responses to each part.

Comment #1-1.1: Is the effect on cardiac hypertrophy physiological and valine specific? Authors presented in Fig. 1 some targeted gene expression analysis focusing on valine gene set. However, several of the validated genes are also involved in other BCAA catabolic activities, including BCKDHA/B and BCAT, upstream of ACAD8.

Response: Thank you for your comment. Existing evidence suggests that alterations in valine metabolism are associated with cardiovascular diseases[1-3]. Notably, in the cardiac tissue and plasma of heart failure patients, valine accumulation is more pronounced than that of isoleucine and leucine[4-5], which led us to focus on valine metabolism.

According to your suggestion, we reanalysed the valine gene set from Fig. 1 after excluding BCKDHA/B and BCAT, and the results confirmed that our original conclusions remained valid: Expression of genes that are involved in the valine metabolism pathway is impaired in pathological cardiac hypertrophy (**Data shown to reviewer#1 Fig. 1a-b**).

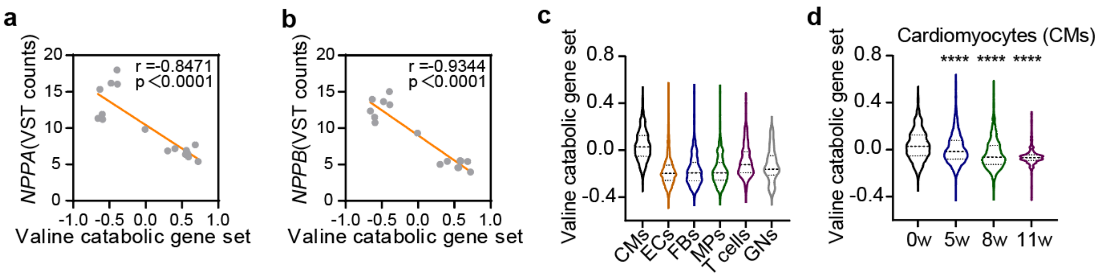

Data shown to reviewer#1 Fig 1 a-b, Scatter plots demonstrating the correlation between valine catabolism gene set scores and *NPPA* and *NPPB* expression levels in the hearts of control (NC) and hypertrophic cardiomyopathy patients (HCM) in the GSE133054 dataset. **c,** Violin plot showing valine catabolism gene set scores of cardiomyocytes (CMs), endothelial cells (ECs), fibroblasts (FBs), macrophages (MPs), T cells and granulocytes (GNs) in mouse hearts according to the GSE120064 dataset. **d,** Violin plot showing valine catabolism gene set scores in CMs from mice at different stages of cardiac hypertrophy according to the GSE120064 dataset (****p<0.0001, compared with 0 w).

However, since BCAA metabolic genes are generally downregulated in cardiac hypertrophy[3], other BCAAs may also play a role in this pathological process. Since the three BCAA metabolisms share many metabolic enzymes, they may also be interacting with each other.

Considering this, we chose ACAD8, an enzyme specifically involved in valine metabolism, to investigate the unique role of valine metabolism in cardiac hypertrophy. Based on this comment, we added the discussion section (page 10 line 337).

"However, the other BCAA metabolic pathways may also play some roles in cardiac hypertrophy. Given that the three BCAAs share some metabolic enzymes, potential interactions between these pathways likely exist. Future studies should systematically investigate the distinct roles of valine, leucine, and isoleucine metabolism in hypertrophic remodeling to elucidate their specific contributions."

Comment #1-1.2: There was no data to support isobutyryl-CoA accumulation in HCM hearts or post-TAC mouse hearts.

Response: Thank you for your insightful comment. This is indeed a constructive suggestion that would ideally be addressed through direct mass spectrometry measurement of isobutyryl-CoA levels in human hypertrophic hearts. However, due to the limited availability of fresh human hypertrophic cardiac tissue samples - with only cryopreserved human hypertrophic cardiac tissue samples in our study - we quantitatively assessed isobutyryl-CoA levels in TAC-induced hypertrophic mouse hearts. Our analyses revealed no statistically significant accumulation of isobutyryl-CoA in 4-week TAC hearts compared to sham-operated controls (**Revised Supplementary Fig. 2b**). This lack of difference may be attributed to the global suppression of valine metabolism genes in the TAC model, which could potentially mask the specific effects of ACAD8 deficiency (**Revised Fig. 1j-k**). Future studies should explore potential isobutyryl-CoA dynamics at different stages of hypertrophy progression or in distinct cell populations.

Nevertheless, clinical evidence supports the significance of ACAD8 and isobutyryl-CoA. Clinically, the dysregulation of isobutyryl-CoA levels due to isobutyryl-CoA dehydrogenase deficiency (IBDD) caused by *ACAD8* mutations is associated with several phenotypes, such as cardiomyopathy, weak muscle tone, and developmental delay[6, 7]. Several studies have shown the diagnostic value of elevated blood levels of isobutyrylcarnitine in patients with cardiac hypertrophy and heart failure, suggesting the potential involvement of isobutyryl-CoA metabolic disturbances in human heart

failure pathogenesis[8, 9]. Collectively, our experimental findings in this research and clinical observations support the pathological significance of ACAD8 deficiency and isobutyryl-CoA metabolic dysregulation in cardiac disease. Future studies will specifically investigate the complete metabolic fate of isobutyryl-CoA in both human and murine hypertrophic cardiac tissues to elucidate its clinical significance further.

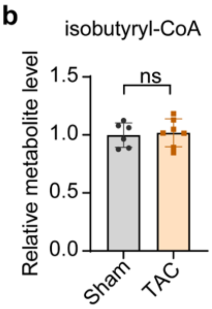

Revised Supplementary Fig. 2 b, Quantification of isobutyryl-CoA levels in Sham-operated and TAC-induced hypertrophic hearts for 4 weeks (n=6-7; ns, not significant).

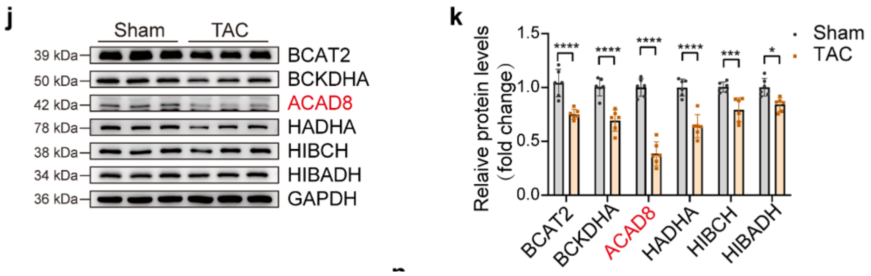

Revised Fig. 1 j, Western blotting and **k,** quantification of the protein expression of valine catabolic genes in mice 4 weeks after Sham or TAC surgery (n=6; *p<0.05, ***p<0.001, ****p<0.0001).

Based on this comment, we added the discussion section (page 11 line 362).

"However, we were unable to confirm the accumulation of isobutyryl-CoA and isobutyrylcarnitine in TAC-induced hypertrophic hearts. This may be attributed to the widespread downregulation of BCAA metabolic genes caused by the TAC model. Therefore, we can only conclude that ACAD8 deficiency can exacerbate TAC-induced pathological cardiac hypertrophy, partially elucidating the mechanisms underlying ACAD8 mutation-mediated cardiac defects in humans. Future studies will specifically investigate the complete metabolic fate of isobutyryl-CoA in both human and murine hypertrophic cardiac tissues to elucidate its clinical significance further."

Comment #1-1.3: The metabolomic data shown in Fig. 3 missed important controls of SHAM animals with and without Acad8 KO in order to directly compare surrogate isobutyryl-carnitine levels in wildtype mice after TAC as well as Acad8KO hearts.

Response: Thank you for your comment. According to your suggestion, we additionally measured isobutyrylcarnitine levels and observed a significant increase in *Acad8*^{cKO} mice compared to controls. However, consistent with the trend observed in isobutyryl-CoA levels, no significant difference in cardiac isobutyrylcarnitine content was detected between Sham and TAC groups in WT mice (**Revised Supplementary Fig. 2a**). As noted previously, this lack of difference may be attributed to the global suppression of valine metabolism genes in the TAC model. Future studies should explore potential isobutyryl-CoA dynamics at different stages of hypertrophy progression or in distinct cell populations. Nevertheless, it maintains significant clinical relevance. Clinically, the dysregulation of isobutyryl-CoA and isobutyrylcarnitine levels due to isobutyryl-CoA dehydrogenase deficiency (IBDD) caused by *ACAD8* mutations is associated with several phenotypes, such as cardiomyopathy, weak muscle tone, and developmental delay[6, 7]. Several studies have shown the diagnostic value of elevated blood levels of isobutyrylcarnitine in patients with cardiac hypertrophy and heart failure, suggesting the potential involvement of isobutyryl-CoA and isobutyrylcarnitine metabolic disturbances in human heart failure pathogenesis[8, 9]. Collectively, our experimental findings in this research and clinical observations support the pathological significance of ACAD8 deficiency and isobutyryl-CoA metabolic dysregulation in cardiac disease. Future studies will specifically investigate the complete metabolic fate of isobutyryl-CoA and isobutyrylcarnitine in both human and murine hypertrophic cardiac tissues to further elucidate their clinical significance.

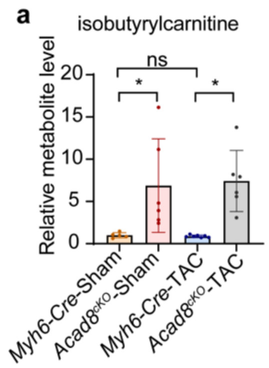

Revised Supplementary Fig. 2 a, Quantification of isobutyrylcarnitine levels in *Myh6-Cre* and *Acad8*^{cKO} mice 4 weeks after Sham or TAC surgery (n=6-7; ns, not significant, *p<0.05).

Comment #1-1.4: In addition, the gene expression altered by isobutyrate treatment in cardiomyocytes also showed significant impact on BCAA catabolism, further raising the question on the specificity of the phenotypic impact from Valine catabolism.

Response: Thank you for your comment. According to your suggestion, we conducted further analysis of the **Revised Fig. 4a**. Isobutyrate treatment caused extensive up-regulation of the mRNA levels of genes downstream of isobutyryl-CoA in valine metabolism (including *Aldh2, Aldh1b1, Aldh9a1, Aox1, Mcee, Hibadh* and *Hadha*), while there was no significant effect on upstream shared genes (including *Bckdha/b* and *Bcat*) (**Data shown to reviewer#1 Fig. 1e**). This may reflect a feedback mechanism to metabolize the excess isobutyryl-CoA. However, alterations in valine metabolism may affect other BCAA metabolism due to the presence of several shared genes (such as *Bckdha/b* and *Bcat*).

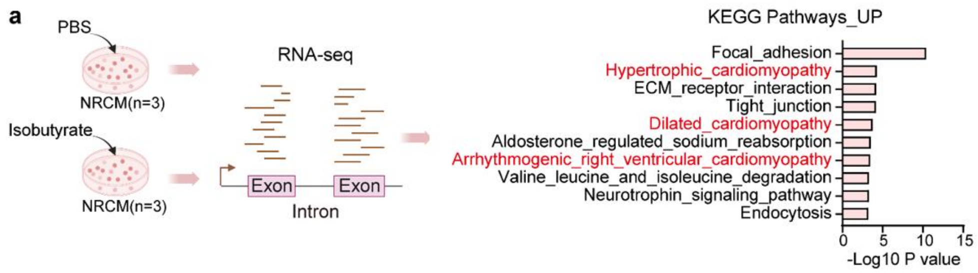

Revised Fig. 4 a, Schematic showing the RNA-seq-based experimental design in which NRCMs were treated with isobutyrate for 36 hours. KEGG functional enrichment analysis of upregulated genes in NRCMs that were treated with isobutyrate.

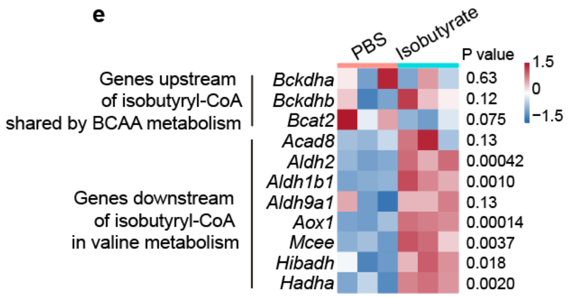

Data shown to reviewer#1 Fig 1 e, Heatmaps of the relative expression of genes associated with BCAA catabolism in NRCMs treated with PBS or isobutyrate based on transcriptomic sequencing data.

Based on this comment, we added the discussion section (page 10 line 337).

"However, the other BCAA metabolic pathways may also play some roles in cardiac hypertrophy. Given that the three BCAAs share some metabolic enzymes, potential interactions between these pathways likely exist. Future studies should systematically investigate the distinct roles of valine, leucine, and isoleucine metabolism in hypertrophic remodeling to elucidate their individual contributions."

<u>**Comment #1-1.5**</u>: Finally, authors used 5mM of isobutyrate in cultured cardiomyocytes

without clear justification. There are reported cytotoxicities of this compound at high enough concentration, and a dosage response should be performed using physiological levels.

Response: Thank you for your comment. Currently, there are no dedicated studies reporting the physiological concentration range of isobutyrate. According to your suggestion, we first performed experiments with a concentration gradient of isobutyrate and found that the expression of hypertrophy-related genes in NRCMs was increased in a dose-dependent manner with isobutyrate, and that it was still able to promote the expression of hypertrophy-related genes at as low as 0.5 mM (**Revised Fig. 3i**). Based on these findings, we selected 5 mM isobutyrate for subsequent experiments to achieve optimal effects.

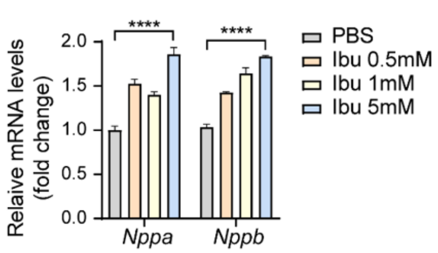

Revised Fig. 3 i, NRCMs were treated with PBS or isobutyrate for 36 hours. qRT-PCR was performed to analyse the mRNA levels of hypertrophic genes (n=3; ****p < 0.0001).

According to your suggestion about potential cytotoxicity induced by 5 mM isobutyrate treatment, we performed CCK-8 assays, which revealed no statistically significant alterations in cellular viability (**Revised Supplementary Fig. 3e**). This finding is consistent with previous research indicating that cytotoxic effects of isobutyrate only become apparent at substantially higher concentrations (22-69 mM)[10].

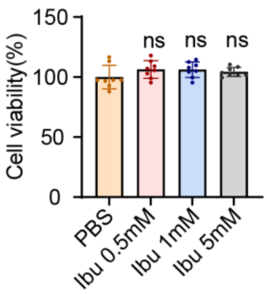

Supplementary Fig. 3 e, CCK-8 assay was performed on NRCMs treated with PBS or isobutyrate at different concentrations for 36 hours (n=8; ns, not significant).

Comment #1-2: Impact of ACAD8 in cardiac hypertrophy was demonstrated by KO in vitro and in vivo. However, the in vitro KD was shown to be sufficient to trigger cardiomyocyte hypertrophy while in vivo KO showed no effect under basal conditions. In the cKO heart, there were no early post-TAC time point measurements for cardiac hypertrophy vs. cardiac function and remodeling. Therefore, it is not clear if the direct impact of KO is on hypertrophy or loss of contractility or other defects such as fibrosis. Considering the mitochondrial localization of valine catabolism, the accumulated metabolites on mitochondrial function, ROS status and bioenergetic properties should be evaluated.

Response: Thank you for your comment. According to your suggestion, we conducted the following experiments. Our experimental evidence suggests that ACAD8 deficiency directly contributes to cardiac hypertrophy.

(1) Our findings in this research demonstrated that *Acad8* knockdown in NRCMs promoted cellular hypertrophy and upregulated the expression of hypertrophy-associated genes (**Revised Fig. 1 l-n**).

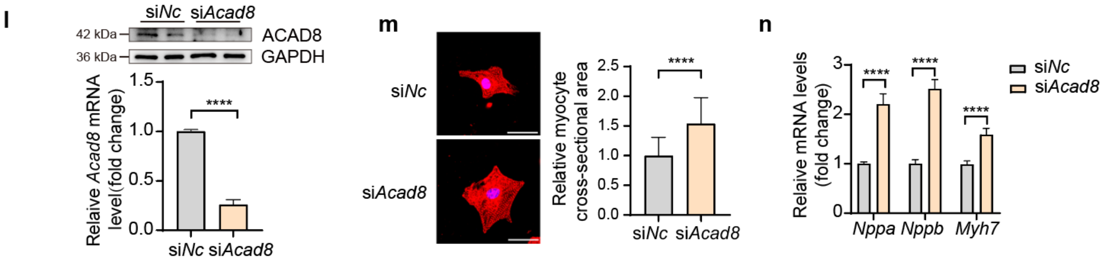

Revised Fig. 1 l, NRCMs were transfected with si*Nc* or si*Acad8* for 48 hours. Western blotting was performed to analyse ACAD8 protein knockdown efficiency (top). qRT-PCR was performed to analyse *Acad8* mRNA knockdown efficiency (n=3; ****p<0.0001) (bottom). **m,** NRCMs were transfected with si*Nc* or si*Acad8* for 48 hours. α-Actinin staining was performed to determine cell size. Representative images (left) and quantification (right) of the sizes of approximately 40 NRCMs in each group are shown (****p<0.0001). Scale bar: 30 μm. **n,** NRCMs were transfected with si*Nc* or si*Acad8* for 48 hours. qRT-PCR was performed to analyse the mRNA expression levels of hypertrophic genes (n=3; ****p<0.0001).

(2) The cardiomyocyte-specific ACAD8 knockout mice did not exhibit spontaneous cardiac hypertrophy; however, they demonstrated an increased susceptibility to developing cardiac hypertrophy when exposed to external stimuli. Compensatory mechanisms may exist in vivo under basal conditions, leading to an inconspicuous phenotype during our observation period (4 weeks). According to your suggestion about *Acad8*^{cKO} under basal conditions, we performed RNA sequencing analysis of cardiac tissue from *Acad8*^{cKO} mice under baseline conditions. RNA sequencing

revealed that *Acad8*^{cKO} hearts under baseline conditions exhibited upregulation of cardiomyopathy-related genes, along with elevated expression of specific cardiac hypertrophy markers (e.g., *Nppa*, *Ankrd1*) (**Revised Supplementary Fig. 1i-j**). Additionally, genes associated with Ca^{2+} handling (e.g., *Atp2a2*, *Ryr2*) and fibrosis-related genes (e.g., *Nfkb1*) also displayed an upward trend, suggesting preserved contractility but a predisposition to fibrosis (**Revised Supplementary Fig. 1j**).

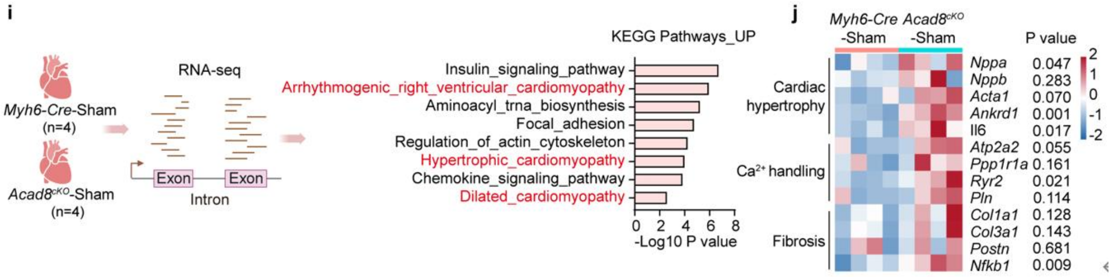

Revised Supplementary Fig. 1 i, KEGG functional enrichment analysis of up-regulated genes in *Acad8*^{cKO}-Sham hearts compared with *Myh6-Cre*-Sham hearts. **j,** Heatmaps of the relative expression of genes associated with cardiac hypertrophy, fibrosis and calcium handling in *Myh6-Cre*-Sham and *Acad8*^{cKO}-Sham hearts based on transcriptomic sequencing data.

(3) Some studies have conducted early cardiac assessment 3-7 days post-TAC surgery[11-13]. According to your suggestion about early post-TAC time point measurements, we evaluated cardiac hypertrophy and contractile function in mice via echocardiography 5 days post-TAC. The results revealed that myocardial hypertrophy (**Revised Supplementary Fig. 1a-c**) and decreased cardiac function (**Revised Supplementary Fig. 1d-f**) both occurred in *Acad8*^{cKO} mice during the early stages of TAC surgery.

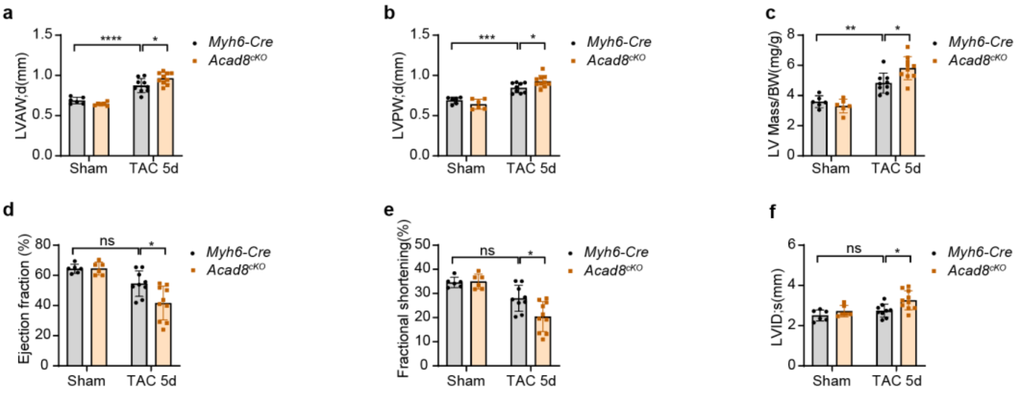

Revised Supplementary Fig. 1 a, Diastolic left ventricular anterior wall thickness (LVAW;d) of *Myh6-Cre* and *Acad8*^{cKO} mice 5 days after Sham or TAC surgery (n=6~10; *p<0.05, ****p<0.0001). **b,** Diastolic left ventricular posterior wall thickness (LVPW;d) of *Myh6-Cre* and *Acad8*^{cKO} mice 5 days after Sham or TAC surgery (n=6~10; *p<0.05, ***p<0.001). **c,** Left

ventricular mass/Body weight (LV Mass/BW) of *Myh6-Cre* and *Acad8*^{cKO} mice 5 days after Sham or TAC surgery (n=6~10; *p<0.05, **p<0.01). **d-f,** Ejection fraction (d)**,** fractional shortening (e) and systolic left ventricular internal dimension (LVID;s) (f) of *Myh6-Cre* and *Acad8*^{cKO} mice 5 days after Sham or TAC surgery (n=6~10; ns, not significant, *p<0.05, ***p<0.001, ****p<0.0001).

(4) According to your suggestion regarding mitochondrial assessments, we performed the following experiments: Transmission electron microscopy (TEM) analysis of cardiac tissue revealed significant reductions in mitochondrial length and density in TAC-operated mice, and Acad8cKO exacerbated these TAC-induced mitochondrial abnormalities. However, under basal conditions, *Acad8*^{cKO} did not alter mitochondrial length or density (**Revised Supplementary Fig. 4a-b**). We further assessed cardiac ROS levels using MitoSOX staining of heart tissue sections. The results revealed that *Acad8*^{cKO} significantly exacerbated TAC-induced ROS accumulation in cardiac tissue. However, *Acad8*^{cKO} did not alter basal ROS production in sham-operated hearts (**Revised Supplementary Fig. 4c-d**). Clinically, patients with ACAD8 mutations exhibit reduced levels of flavins, which may impair electron transport chain activity, thereby affecting cellular energy production and contributing to the generation of ROS[14]. ACAD8 mutant mice display increased hepatic steatosis and mitochondrial damage[15]. Thus, ACAD8 may also contribute to cardiac physiology and pathology through additional mechanisms, such as influencing mitochondrial function.

We further assessed whether ACAD8 deficiency affects mitochondria-associated metabolites. The levels of acylcarnitines derived from medium- and long-chain fatty acids were significantly reduced in TAC-operated *Acad8*^{cKO} hearts, indicating impaired fatty acid oxidation (**Revised Supplementary Fig. 4e**). While acetyl-CoA levels exhibited a downward trend, this change did not reach statistical significance (**Revised Supplementary Fig. 4f**). However, circulating Krebs cycle metabolites (e.g., succinate, fumarate, and malate) remained unchanged in TAC-operated *Acad8*^{cKO} hearts, and levels of α-ketoglutarate increased, which suggests the presence of metabolic compensation mechanisms (**Revised Supplementary Fig. 4g**).

We found a trend towards increased glycolytic metabolites in TAC-operated *Acad8*^{cKO} hearts. Furthermore, significant accumulation of multiple amino acids (e.g., ornithine, phenylalanine, and methionine) and widespread alterations in amino acid turnover-related metabolites were observed (**Revised Supplementary Fig. 4h-i**). Thus, the increased glycolysis and amino acids may have compensated to some extent for the flux of the Krebs cycle.

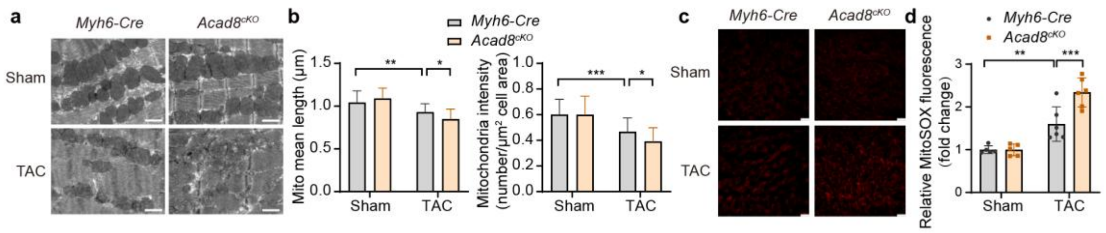

Revised Supplementary Fig. 4 a, Representative electron microscope images of cardiomyocyte mitochondria in *Myh6-Cre* and *Acad8^{cKO}* mice 4 weeks after Sham or TAC surgery. Scale Bar =1μm. **b,** Quantification of mitochondrial mean length and mitochondrial density in heart tissues (n=4; *p<0.05, **p<0.01, ***p<0.001). **c,** Representative image and **d,** quantification of relative MitoSOX Red fluorescence in *Myh6-Cre* and *Acad8^{cKO}* mice 4 weeks after Sham or TAC surgery (n=5~6; **p<0.01, ***p<0.001). Scale Bar = 30 μm.

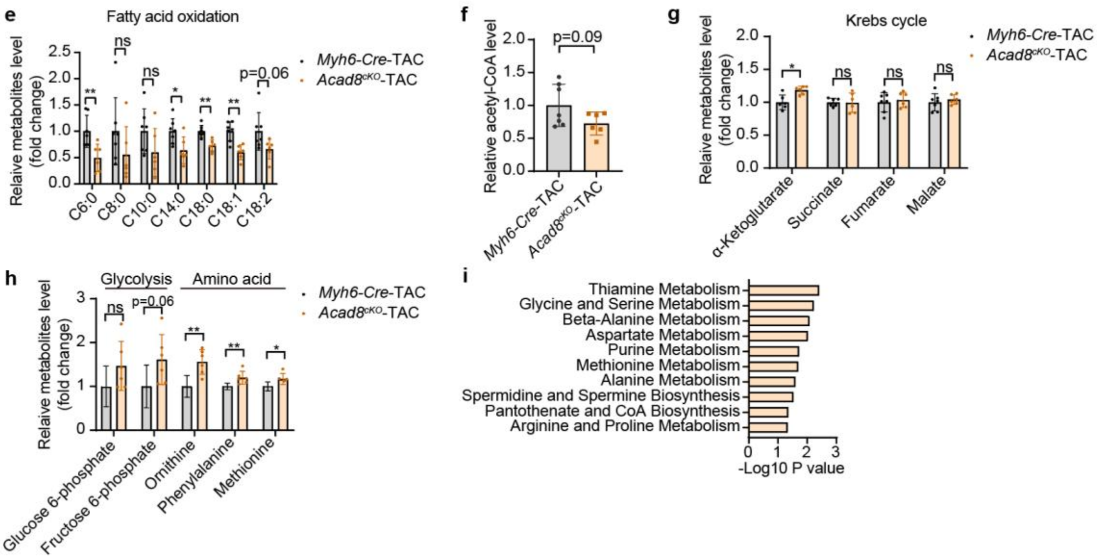

Revised Supplementary Fig. 4 e, Quantification of acylcarnitines in *Myh6-Cre*-TAC and *Acad8^{cKO}*-TAC hearts. (n=6-7; ns: not significant, *p<0.05, **p<0.001). **f,** Quantification of acetyl-CoA levels in *Myh6-Cre*-TAC and *Acad8^{cKO}*-TAC hearts. (n=6-7). **g,** Quantification of metabolites associated with the Krebs cycle in *Myh6-Cre*-TAC and *Acad8^{Cko}*-TAC hearts. (n=5-7; ns: not significant, *p<0.05). **h,** Quantification of metabolites associated with glycolysis and amino acid in *Myh6-Cre*-TAC and *Acad8^{cKO}*-TAC hearts. (n=5-7; ns: not significant, *p<0.05). **i,** Pathways significantly enriched for differential metabolites in *Myh6-Cre*-TAC and *Acad8^{cKO}*-TAC hearts.

Collectively, these findings demonstrated that ACAD8 deficiency promoted cardiac hypertrophy. Although no overt phenotypic changes were observed under basal conditions, elevated expression of hypertrophy-associated genes was already detectable. Following TAC stimulation, ACAD8^{cKO} hearts exhibited exacerbated upregulation of pro-hypertrophic genes, ultimately developing pathological cardiac hypertrophy accompanied by contractile dysfunction and mitochondrial impairment.

Based on this comment, we added the discussion section (page 11 line 350 and page 12 line 408).

"The cardiomyocyte-specific ACAD8 knockout mice did not exhibit spontaneous cardiac hypertrophy; however, molecular analyses revealed elevated expression of hypertrophy-associated genes under baseline conditions. When subjected to external stress, these mice exhibited significantly increased susceptibility to pathological cardiac remodeling."

"Moreover, we observed that cardiomyocyte-specific ACAD8 knockout exacerbated TAC-induced mitochondrial structural and functional impairments. Clinically, patients with ACAD8 mutations exhibit reduced levels of flavins, which may impair electron transport chain activity, thereby affecting cellular energy production and contributing to the generation of reactive oxygen species (ROS) [45,46]. ACAD8 mutant mice display increased hepatic steatosis and mitochondrial damage[1]. These studies also suggest that ACAD8 may also contribute to cardiac physiology and pathology through additional mechanisms, which remain to be fully elucidated."

Comment #1-3: The mechanism of action for ACAD8 deficiency and valine catabolic defect was investigated in the KO hearts/cells by transcriptomic profiling and targeted epigenetic analysis. However, the implication of TEAD2 was speculated but not proven. Direct impact on TEAD2 expression or activity was not demonstrated. The chromatin opening was a reflection of and consistent with the targeted gene induction, but the underlying mechanism remains to be elucidated.

Response: Thank you for your comment. (1) According to your suggestion about TEAD2, we first examined the expression levels of TEAD2. However, neither isobutyrate treatment nor ACAD8 deficiency significantly altered *Tead2* expression levels (**Revised Supplementary Fig. 5a-b**). We therefore hypothesized that TEAD2 occupancy at the promoters of hypertrophic genes might be altered. We initially attempted to perform CUT&Tag experiments using a commercially available TEAD2 antibody, but these efforts proved unsuccessful. Consequently, we overexpressed TEAD2-FLAG in NRCMs and subsequently conducted both CUT&Tag and qRT-PCR assays using an anti-FLAG antibody to verify the binding sites of TEAD2 in the promoter regions of *Anp* and *Bnp* and found that both isobutyrate treatment and knockdown of *Acad8* were able to promote the binding of TEAD2 in the promoter regions of *Nppa* and *Nppb* (**Revised Fig. 4 f-g**). We next overexpressed TEAD2 in NRCMs to validate its function in cardiac hypertrophy. The results demonstrated that

overexpression of TEAD2 promoted the expression of *Anp* induced by *Acad8* knockdown and isobutyrate treatment (**Revised Fig. 4 h-j**). This result suggests that TEAD2 is capable of regulating the expression of genes associated with cardiac hypertrophy.

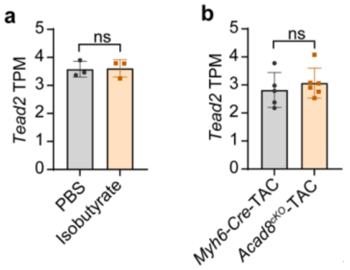

Revised Supplementary Fig. 5 a, *Tead2* expression in NRCMs treated with PBS or isobutyrate from RNA-seq data (n=3; ns, not significant). **b,** *Tead2* expression in Myh6-Cre-TAC and *Acad8*^{cKO}-TAC hearts from RNA-seq data (n=5-6; ns, not significant).

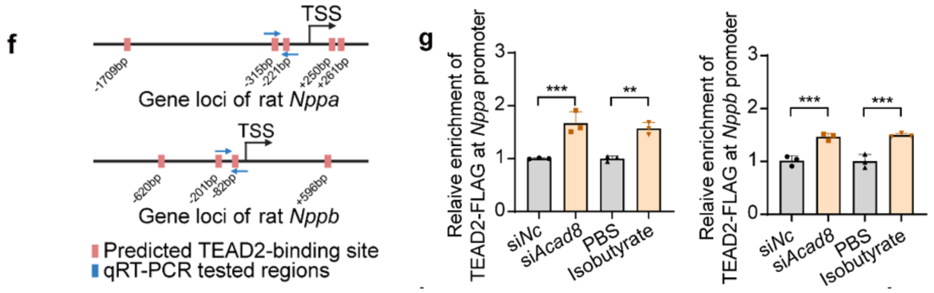

Revised Fig. 4 f, Schematic showing the predicted binding sites and validation regions for TEAD2 in the *Nppa* and *Nppb* promoter regions. The exact positions relative to the TSS are labelled below. **g,** NRCMs were transfected with Ad*TEAD2*, then treated with isobutyrate or transfected with si*Nc* or si*Acad8*, followed by CUT-Tag and qRT-PCR experiments (n=3; **p<0.01, ***p<0.001).

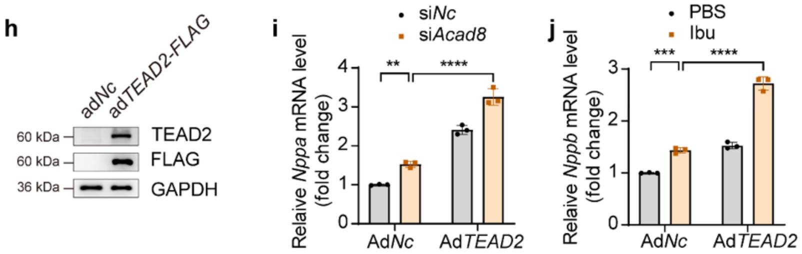

Revised Fig 4 h, Western blotting of the protein expression of TEAD2 in NRCMs transfected with Ad*Nc* or Ad*TEAD2* for 48 hours. **i,** NRCMs were transfected with Ad*Nc* or Ad*TEAD2* for 36 hours after transfection with si*Nc* or si*Acad8* for 36 hours (n=3; **p < 0.01, ****p < 0.0001). **j,** NRCMs were transfected with Ad*Nc* or Ad*TEAD2* for 36 hours after treatment with PBS or isobutyrate for 36 hours (n=3; ***p < 0.001, ****p < 0.0001). qRT–PCR was performed to analyse the mRNA expression levels of hypertrophic genes.

(2) According to your suggestion about chromatin opening, we conducted additional

exploratory analyses to validate our initial findings. Histone post-translational modifications may confer distinctive effects on chromatin architecture and its interactions with non-histone proteins, thereby modulating both chromatin structure and transcriptional regulation. Isobutyrate treatment specifically enhanced intracellular isobutyryl-CoA concentrations, which can function as acyl donors to mediate lysine isobutyrylation (Kibu) modifications on histone H3[13], ultimately influencing chromatin accessibility and gene expression profiles.

We used anti-Kibu antibodies for verification and observed elevated isobutyrylation modifications of H3K9 and H3K23 in TAC-operated ACAD8-deficient mouse hearts (**Revised Fig. 3d-e**). Further, *Acad8* knockdown in NRCMs enhanced the isobutyrate-induced isobutyrylation modifications of H3K9 and H3K23 (**Revised Supplementary Fig. 3b-d**).

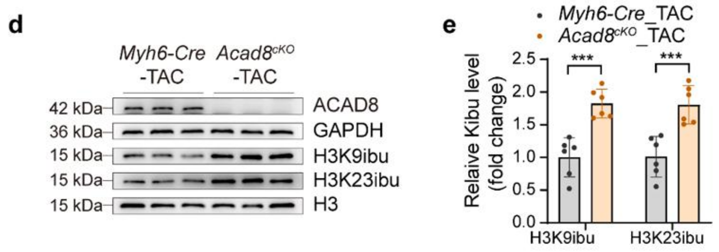

Revised Fig. 3 d, Western blotting and **e,** Quantification of H3k9ibu and H3k23ibu levels in *Myh6-Cre*-TAC and *Acad8*^{cKO}–TAC hearts (n=6; ***p < 0.001).

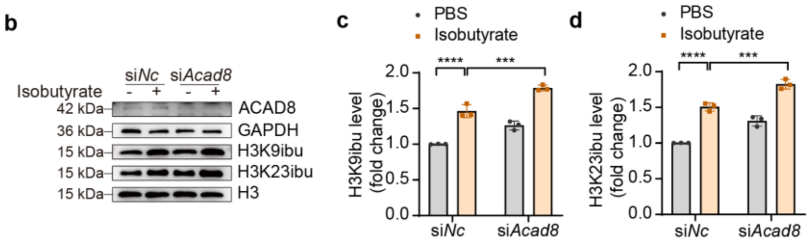

Revised Supplementary Fig. 3 b, Western blotting and **c-d,** Quantification of H3K9ibu and H3K23ibu levels in NRCMs treated with PBS or isobutyrate (5mM) for 36 hours after transfection with si*Nc* or si*Acad8* for 36 hours (n=3; ***p＜0.001, ****p＜0.0001).

The results in this research demonstrated that both isobutyrate treatment and *Acad8* knockdown significantly enhanced chromatin accessibility at the promoter regions of hypertrophy markers *Nppa* and *Nppb* (**Revised Fig. 4k-l**). Therefore, increased isobutyryl-CoA and isobutyrylation modifications may promote chromatin opening, potentially enhancing TEAD2 binding at hypertrophy gene promoters.

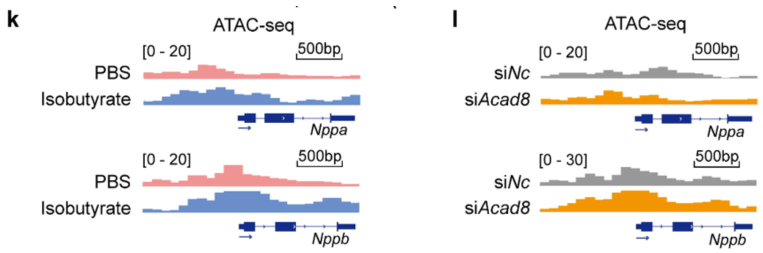

Revised Fig. 4 k, IGV images showing ATAC-seq peaks in NRCMs that were treated with PBS or isobutyrate for 36 hours, aligned across the *Nppa* (top) and *Nppb* (bottom) gene coordinates. **l**, IGV images showing ATAC-seq peaks in NRCMs that were transfected with si*Nc* or si*Acad8* for 48 hours, aligned across the *Nppa* (top) and *Nppb* (bottom) gene coordinates.

Based on this comment, we added the discussion section (page 11 line 379 and page 12 line 402).

"Our study demonstrated that the isobutyrylation of histone H3K9 and H3K23 were significantly elevated in the hearts of *Acad8*-deficient mice subjected to TAC, Similarly, knockdown of *Acad8* in NRCMs promoted isobutyrate-induced isobutyrylation at H3K9 and H3K23. Future studies should further elucidate the regulatory mechanisms of histone isobutyrylation on chromatin remodelling and gene expression."

"These findings suggest that TEAD2 may play a regulatory role in the transcriptional control of hypertrophy-related genes. ACAD8 deficiency leads to the accumulation of isobutyryl-CoA. The ensuing increase in histone isobutyrylation modifications may alter chromatin accessibility within the promoter regions of hypertrophic genes, potentially influencing the binding of transcription factors such as TEAD2 and thereby modulating gene expression."

Comment #1-4: The ACAD8 OE showed a significant cardio-protection against pathological hypertrophy. This was a very exciting result considering the OE was implemented after one week of TAC. Here, the analysis stayed at phenotypic level and the mechanistic investigation was not performed. Valine related metabolites, gene expression (epigenetic markers as shown in Fig. 3), mito-function et al should be investigated to support the central hypothesis and the working model.

Response: Thank you for your comment. According to your suggestions, we have conducted follow-up experiments to elucidate the protective mechanisms of ACAD8-OE, focusing on three aspects: valine-related metabolites, metabolic gene expression analysis, and mitochondrial function. Our findings demonstrated that ACAD8 overexpression (ACAD8-OE) reduced isobutyryl-CoA levels, leading to the

downregulation of hypertrophy-associated genes and partial restoration of mitochondrial function.

(1) ACAD8-OE reduced isobutyryl-CoA levels. According to your suggestion about valine-related metabolites, we measured the levels of isobutyryl-CoA in AAV9-Ctrl-TAC and AAV9-*ACAD8*-TAC hearts, and the results showed that ACAD8-OE reduced the levels of isobutyryl-CoA in the heart (**Revised Fig. 6a**). We also validated the effect of ACAD8-OE on the isobutyrylation modifications of histone H3. Our results revealed a decrease in isobutyrylation of H3K9, and H3K23 in TAC-operated AAV9-cTnT-*ACAD8* mice (**Revised Fig. 6b-c**).

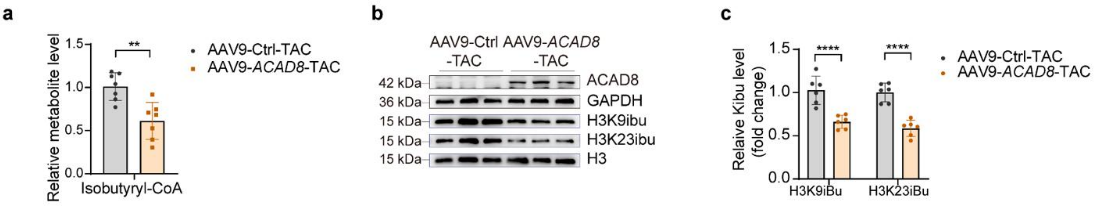

Revised Fig. 6 a, Quantification of isobutyryl-CoA levels in AAV9-Ctrl-TAC and AAV9-*ACAD8*-TAC hearts (n=7; **p<0.01)**. b,** Western blotting and **c,** Quantification of H3K9ibu, and H3K23ibu levels in AAV9-Ctrl-TAC and AAV9-*ACAD8*-TAC hearts (n=6; ****p<0.0001).

(2) ACAD8-OE downregulated the expression of hypertrophy-associated genes. According to your suggestion about gene expression, we further performed RNA sequencing on hearts from AAV9-Ctrl-TAC and AAV9-*ACAD8*-TAC mice. KEGG pathway analysis showed downregulation of genes related to various cardiomyopathies and significant reduction of cardiac hypertrophy markers in AAV9-*ACAD8*-TAC hearts (**Revised Fig. 6d-e**).

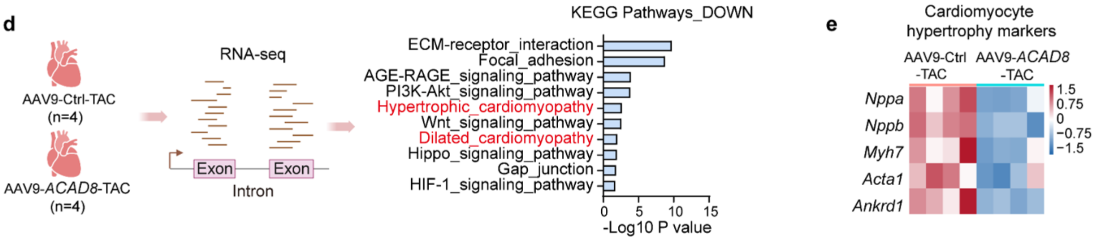

Revised Fig. 6 d, Schematic showing the RNA-seq-based experimental design for analysing the left ventricles of AAV9-Ctrl-TAC and AAV9-*ACAD8*-TAC hearts. KEGG functional enrichment analysis of downregulated genes in TAC-operated AAV9-*ACAD8*-TAC hearts. **e,** Heatmaps of the relative expression of the differentially expressed genes associated with cardiomyocyte hypertrophy that were identified in AAV9-Ctrl-TAC and AAV9-*ACAD8*-TAC hearts.

(3) ACAD8-OE ameliorated mitochondrial damage. According to your suggestion regarding mitochondrial function, we performed additional experiments. In TAC-operated hearts, ACAD8-OE increased acetyl-CoA levels (**Revised Supplementary Fig. 6d**). TEM of cardiac tissue showed that ACAD8-OE ameliorated the TAC-induced reductions in mitochondrial length and mitochondrial density (**Revised Supplementary Fig. 6e-f**). Additionally, it alleviated TAC-induced ROS accumulation in cardiac tissue (**Revised Supplementary Fig. 6g-h**).

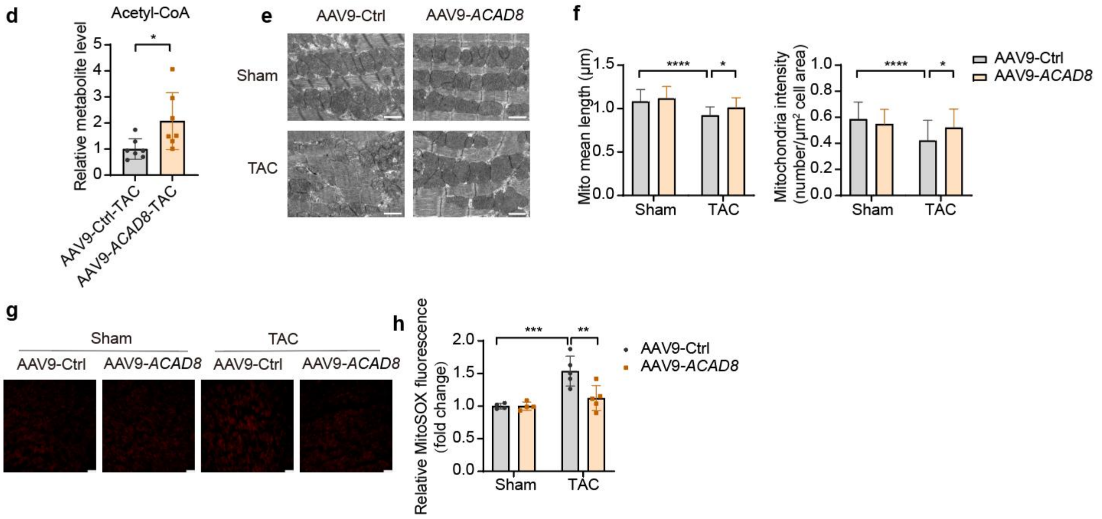

Revised Supplementary Fig. 6 d, Quantification of acetyl-CoA levels in *Myh6-Cre*-TAC and *Acad8*^{cKO}–TAC hearts. (n=7; *p<0.05). **e,** Representative electron microscope images of cardiomyocyte mitochondria in the hearts of mice infected with AAV9-cTnT-Ctrl or AAV9-cTnT-*ACAD8* 4 weeks after Sham or TAC surgery. Scale Bar =1μm. **f,** Quantification of mitochondrial mean length and mitochondrial density in heart tissues (n=4; *p<0.05, ****p<0.0001). **g,** Representative image and **h,** quantification of relative MitoSOX Red fluorescence in the hearts of mice infected with AAV9-cTnT-Ctrl or AAV9-cTnT-*ACAD8* 4 weeks after Sham or TAC surgery (n=4~5; **p<0.01, ***p<0.001). Scale Bar = 30 μm.

(4) The results in this research demonstrated that ACAD8-OE in primary cardiomyocytes effectively ameliorated isobutyrate-induced accumulation of isobutyryl-CoA and elevation of histone H3K9 and H3K23 isobutyrylation modifications, while attenuating isobutyrate-triggered cardiomyocyte hypertrophy (**Revised Fig. 6f-k**).

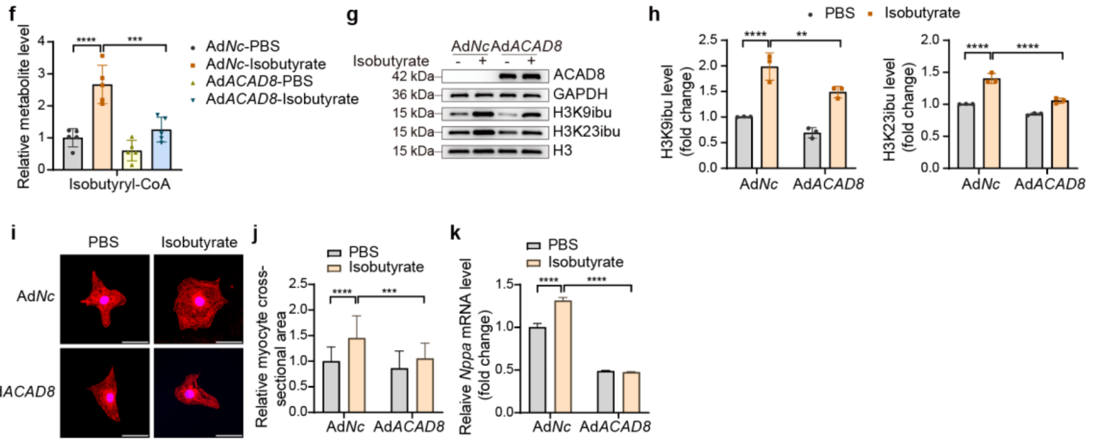

Revised Revised Fig. 6 f, Quantification of isobutyryl-CoA levels in NRCMs that were infected with the indicated adenovirus for 24 hours and then treated with PBS or Isobutyrate for 48 h (n=5; ns: not significant, ***p<0.001, ****p<0.0001). **g,** Western blotting and **h,** Quantification of H3K9ibu and H3K23ibu levels in NRCMs treated with PBS or isobutyrate (5mM) for 36 hours after infection with ad*Nc* or ad*ACAD8* for 36 hours (n=3; **p＜0.01, ****p＜0.0001). **i-j,** NRCMs were infected with the indicated adenovirus for 24 hours and then treated with PBS or isobutyrate for 48 hours. α-Actinin staining was performed to determine cell size. Representative images and quantification of the sizes of approximately 20 NRCMs in each group are shown (***p<0.001, ****p<0.0001). Scale bar: 30 μm. **k,** NRCMs were infected with the indicated adenovirus for 24 hours and then treated with PBS or isobutyrate for 48 hours. qRT–PCR was performed to analyse the mRNA expression level of *Nppa* (n=3; ****p<0.0001).

In summary, our findings demonstrate that ACAD8-OE confers comprehensive cardioprotection by reducing isobutyryl-CoA accumulation, suppressing hypertrophy-related gene expression, and improving mitochondrial function, thereby effectively rescuing TAC-induced pathological cardiac hypertrophy.

Reference

1. Jang, C., et al., *A branched-chain amino acid metabolite drives vascular fatty acid transport and causes insulin resistance*. Nat Med, 2016. 22(4): p. 421-6.
2. Xu, Y., et al., *Branched-Chain Amino Acid Catabolism Promotes Thrombosis Risk by Enhancing Tropomodulin-3 Propionylation in Platelets*. Circulation, 2020. 142(1): p. 49-64.
3. Sun, H., et al., *Catabolic Defect of Branched-Chain Amino Acids Promotes Heart Failure*. Circulation, 2016. 133(21): p. 2038-49.
4. Previs, M.J., et al., *Defects in the Proteome and Metabolome in Human Hypertrophic Cardiomyopathy*. Circ Heart Fail, 2022. 15(6): p. e009521.
5. Hahn, V.S., et al., *Myocardial Metabolomics of Human Heart Failure With Preserved Ejection Fraction*. Circulation, 2023. 147(15): p. 1147-1161.
6. Oglesbee, D., et al., *Development of a newborn screening follow-up algorithm for the diagnosis of isobutyryl-CoA dehydrogenase deficiency.* Genet Med, 2007. 9(2): p. 108-16.
7. Zhuang, D.Y., et al., *Identification of Six Novel Variants of ACAD8 in Isobutyryl-CoA Dehydrogenase Deficiency With Increased C4 Carnitine Using Tandem Mass Spectrometry and NGS Sequencing.* Front Genet, 2021. 12: p. 791869.
8. Cheng, M.L., et al., *Metabolic disturbances identified in plasma are associated with outcomes in patients with heart failure: diagnostic and prognostic value of metabolomics.* J Am Coll Cardiol, 2015. **65**(15): p. 1509-20.
9. Zordoky, B.N., et al., *Metabolomic fingerprint of heart failure with preserved ejection fraction.* PLoS One, 2015. **10**(5): p. e0124844.
10. Sakurazawa, T. and T. Ohkusa, *Cytotoxicity of organic acids produced by anaerobic intestinal bacteria on cultured epithelial cells.* J Gastroenterol, 2005. **40**(6): p. 600-9.
11. Li, L., et al., *ATP6AP2 knockdown in cardiomyocyte deteriorates heart function via compromising autophagic flux and NLRP3 inflammasome activation.* Cell Death Discov, 2022. 8(1): p. 161.
12. Li, Q., et al., *PKM1 Exerts Critical Roles in Cardiac Remodeling Under Pressure Overload in the Heart.* Circulation, 2021. 144(9): p. 712-727.
13. Anand, P., et al., *BET bromodomains mediate transcriptional pause release in heart failure.* Cell, 2013. 154(3): p. 569-82.
14. Tummolo, A., et al., *Combined isobutyryl-CoA and multiple acyl-CoA dehydrogenase deficiency in a boy with altered riboflavin homeostasis.* JIMD Rep, 2022. 63(4): p. 276-291.

15. Sabbagha, N.G., et al., *Alternative splicing in Acad8 resulting a mitochondrial defect and progressive hepatic steatosis in mice.* Pediatr Res, 2011. 70(1): p. 31-6.

Response to Reviewer #2

The authors show that valine metabolism is downregulated particularly at the level of ACAD8 in response to pressure overload, which may induce accumulation isobutyrycarnitine, thereby inducing cardiac hypertrophy.

Dear reviewer,

Thank you for reviewing our manuscript and for the constructive suggestions. We have carefully considered your suggestions and conducted additional experiments to address them. We hope that we have understood your concerns and addressed them accurately. The manuscript has been revised accordingly, and the major revised parts have been highlighted in yellow. A point-by-point response to the comments is listed below. The figures shown below are labelled in the order as in the manuscript.

Comment #2-1: The authors show that valine metabolism is downregulated particularly at the level of ACAD8 in response to pressure overload, which may induce accumulation isobutyrycarnitine, thereby inducing cardiac hypertrophy. TAC tends to downregulate many enzymes on the valine metabolic pathway. The authors failed to show a specific accumulation of isobutyrylcarnitine in the control heart in response to TAC. Thus, it is hard to conclude that the block at the level of ACAD8 really is the key mechanism mediating pathological hypertrophy in the heart. Although the authors conducted rescue by ACAD8, the extent of recure of endogenous ACAD8 and its metabolite is never shown. In addition, the effect can also be mediated through valine metabolites downstream of ACAD8.

Response: Thank you for your comment. According to your suggestions about the significance of ACAD8, we have conducted follow-up experiments and analyses: (1) Quantitative measurements of isobutyryl-CoA and isobutyrylcarnitine in TAC-induced hypertrophic hearts, along with re-evaluation of both clinical and experimental evidence supporting ACAD8's important role; (2) Supplemental examinations of ACAD8-OE effects on endogenous *Acad8* expression and its associated metabolites. To ensure a clear and organized response, we have divided your original comment into two parts based on the different aspects you raised. Below are our detailed responses to each part.

(1) Defects in multiple genes of the valine catabolic pathway are known to cause human genetic disorders, underscoring the research significance of each component in this pathway[2,3]. We selected ACAD8 as our focus because it exhibits the most pronounced downregulation in both human and murine hypertrophic hearts (**Fig. 1d-e**

and Revised Fig. 1 j-k). Clinically, the dysregulation of isobutyryl-CoA levels due to isobutyryl-CoA dehydrogenase deficiency (IBDD) caused by *ACAD8* mutations is associated with several phenotypes, such as cardiomyopathy, weak muscle tone, and developmental delay[4,5]. These clinical observations support the significance of investigating ACAD8 deficiency and isobutyryl-CoA metabolic dysregulation in cardiac disease. Several studies have shown the diagnostic value of elevated blood levels of isobutyrylcarnitine in patients with cardiac hypertrophy and heart failure, suggesting the potential involvement of isobutyryl-CoA metabolic disturbances in human heart failure pathogenesis[6,7].

However, the global suppression of valine metabolic genes in the TAC model may have masked the specific effects of ACAD8 deficiency (**Revised Fig. 1j-k**), potentially explaining the absence of significant isobutyryl-CoA or isobutyrylcarnitine accumulation in TAC-induced hypertrophic hearts (**Revised Supplementary Fig. 2a-b**). Future studies will specifically investigate the complete metabolic fate of isobutyryl-CoA and isobutyrylcarnitine in both human and murine hypertrophic cardiac tissues to further elucidate their clinical significance.

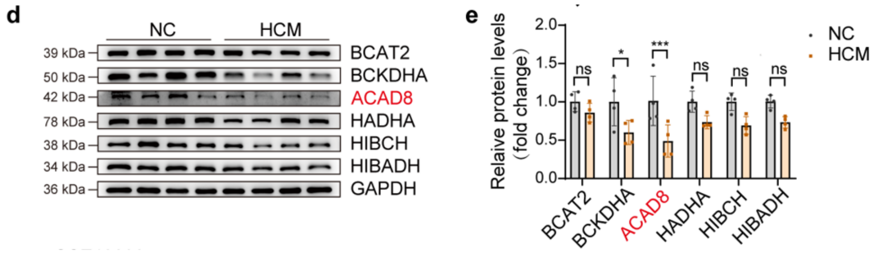

Fig. 1 d, Western blotting and **e,** quantification of the protein expression of valine catabolic genes in the hearts of control and hypertrophic cardiomyopathy patients (n=4; ns: not significant, *p<0.05, ***p<0.001).

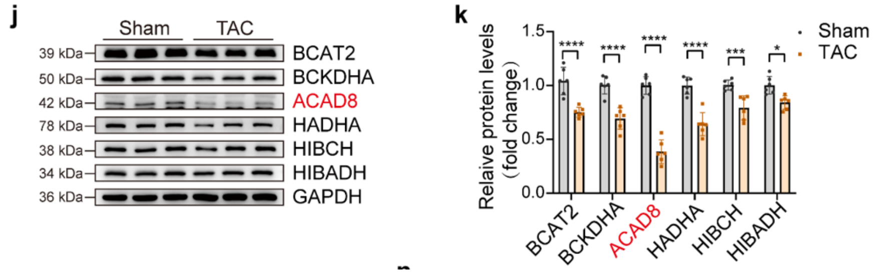

Revised Fig. 1 j, Western blotting and **k,** quantification of the protein expression of valine catabolic genes in mice 4 weeks after Sham or TAC surgery (n=6; *p<0.05, ***p<0.001, ****p<0.0001).

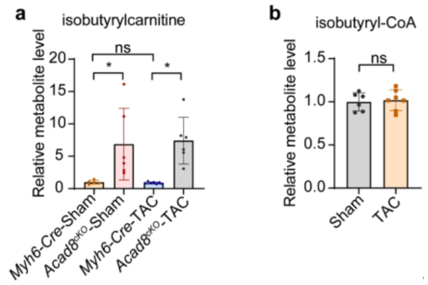

Revised Supplementary Fig. 2 a, Quantification of isobutyrylcarnitine levels in *Myh6-Cre* and *Acad8^{cKO}* mice 4 weeks after Sham or TAC surgery (n=6-7; ns, not significant, *p<0.05). **b,** Quantification of isobutyryl-CoA levels in Sham-operated and TAC-induced hypertrophic hearts for 4 weeks (n=6-7; ns, not significant).

Based on this comment, we added the discussion section (page 11 line 362).

"However, we were unable to confirm the accumulation of isobutyryl-CoA and isobutyrylcarnitine in TAC-induced hypertrophic hearts. This may be attributed to the widespread downregulation of BCAA metabolic genes caused by the TAC model. Therefore, we can only conclude that ACAD8 deficiency can exacerbate TAC-induced pathological cardiac hypertrophy, partially elucidating the mechanisms underlying cardiac defects in humans caused by ACAD8 mutation. Future studies will specifically investigate the complete metabolic fate of isobutyryl-CoA in both human and murine hypertrophic cardiac tissues to elucidate its clinical significance further."

(2) Our work represents a novel contribution to cardiovascular research, as ACAD8's role in cardiac pathophysiology had not been previously investigated. Genetic knockout and overexpression experiments in this research demonstrate that ACAD8 functionally regulates TAC-induced cardiac hypertrophy by modulation of isobutyryl-CoA levels. According to your suggestion about endogenous ACAD8, the mRNA expression level of endogenous *Acad8* was verified in **Revised Supplementary Fig. 6c**. The results indicate that ACAD8-OE by AAV9 (human *ACAD8*) in TAC hearts causes a rebound of endogenous *Acad8* (murine *Acad8*).

According to your suggestion about valine metabolites, we measured the levels of isobutyryl-CoA in AAV9-Ctrl-TAC and AAV9-*ACAD8*-TAC hearts, and the results showed that ACAD8-OE reduced the levels of isobutyryl-CoA in the heart (**Revised Fig. 6a**). Our results revealed a decrease in isobutyrylation of H3K9, and H3K23 in TAC-operated AAV9-cTnT-*ACAD8* mice (**Revised Fig. 6b-c**).

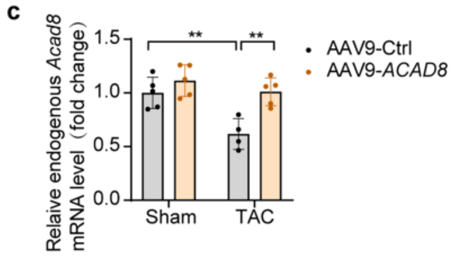

Supplementary Fig. 6 c, qRT-PCR was performed to analyse the mRNA expression level of endogenous ACAD8 in mice infected with AAV9-Ctrl and AAV9-*ACAD8* after Sham or TAC surgery (n=4~5; **p<0.01).

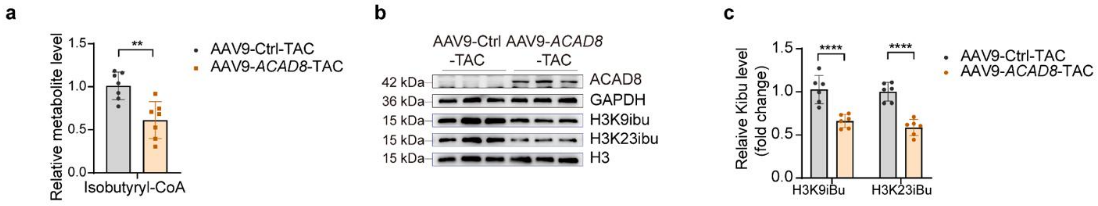

Revised Fig. 6 a, Quantification of isobutyryl-CoA levels in AAV9-Ctrl-TAC and AAV9-*ACAD8*-TAC hearts (n=7; **p<0.01)**. b,** Western blotting and **c,** Quantification of H3K9ibu, and H3K23ibu levels in AAV9-Ctrl-TAC and AAV9-*ACAD8*-TAC hearts (n=6; ****p<0.0001).

We acknowledge your perspective that the downstream metabolites of ACAD8 may also undergo changes and exert biological effects. However, due to technical and temporal limitations, these were not examined in our current study. Our study focused on isobutyryl-CoA accumulation as the primary mediator linking ACAD8 deficiency to TAC-induced cardiac hypertrophy.

Based on this comment, we added the discussion section (page 13 line 424 and page 11 line 371).

"Our results demonstrate that ACAD8-OE significantly reduces isobutyryl-CoA levels and histone isobutyrylation modifications in TAC-induced hypertrophic hearts, concurrently suppressing the expression of hypertrophy-related genes."

"While our study focused on isobutyryl-CoA accumulation as the primary mediator linking ACAD8 deficiency to TAC-induced cardiac hypertrophy, we acknowledge that other downstream metabolites in this pathway may also contribute to cardiac hypertrophy. Future studies should investigate whether these metabolites accumulate in hypertrophic hearts and define their specific roles in disease progression."

Comment #2-2: Isobutyrate treatment has many metabolic effects besides the accumulation of isobutyryl-CoA and isobutyrylcarnitine. It is unclear whether isobutyrate treatment really mimics the effect of ACAD8.

Response: Thank you for your comment. We acknowledge that isobutyrate may have multiple metabolic roles beyond its involvement in valine catabolism. However, our data demonstrated that isobutyrate treatment at least partially recapitulated the isobutyryl-CoA accumulation and phenotypic manifestations associated with ACAD8 deficiency. We sincerely apologize for any lack of clarity in our original manuscript.

(1) Both isobutyrate treatment and ACAD8 deficiency promote the accumulation of isobutyryl-CoA and isobutyrylation modifications. In contrast, overexpression of ACAD8 ameliorated the accumulation of isobutyryl-CoA and isobutyrylation modifications induced by isobutyrate treatment (**Revised Fig. 6f-h**).

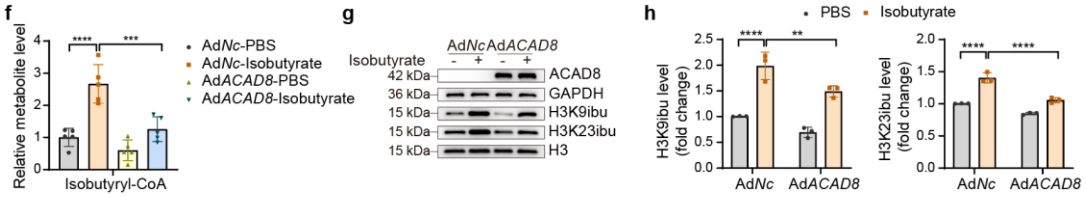

Revised Fig. 6 f, Quantification of isobutyryl-CoA levels in NRCMs that were infected with the indicated adenovirus for 24 hours and then treated with PBS or Isobutyrate for 48 h (n=5; ns: not significant, ***p<0.001, ****p<0.0001). **g,** Western blotting and **h,** Quantification of H3K9ibu and H3K23ibu levels in NRCMs treated with PBS or isobutyrate (5mM) for 36 hours after infection with Ad*Nc* or Ad*ACAD8* for 36 hours (n=3; **p＜0.01, ****p＜0.0001).

(2) Both isobutyrate treatment and ACAD8 deficiency promote cardiomyocyte hypertrophy (**Revised Fig. 1l-n and Revised Fig. 3g-h**).

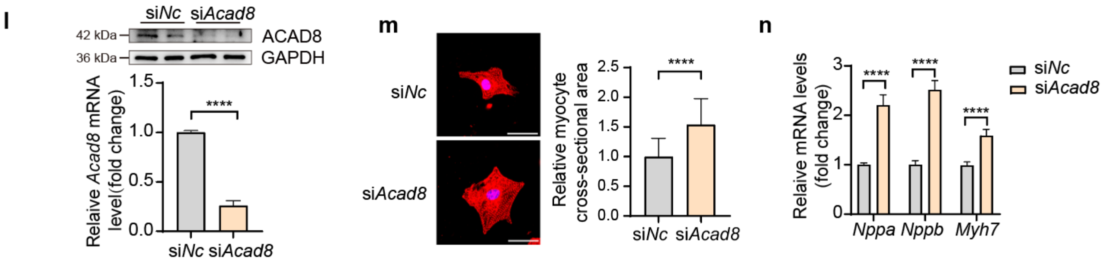

Revised Fig. 1 l, NRCMs were transfected with si*Nc* or si*Acad8* for 48 hours. Western blotting was performed to analyse ACAD8 protein knockdown efficiency (top). qRT-PCR was performed to analyse *Acad8* mRNA knockdown efficiency (n=3; ****p<0.0001) (bottom). **m,** NRCMs were transfected with si*Nc* or si*Acad8* for 48 hours. α-Actinin staining was performed to determine cell size. Representative images (left) and quantification (right) of the sizes of approximately 40 NRCMs in each group are shown (****p<0.0001). Scale bar: 30 μm. **n,** NRCMs were transfected with si*Nc* or si*Acad8* for 48 hours. qRT-PCR was performed to analyse the mRNA expression levels of hypertrophic genes (n=3; ****p<0.0001).

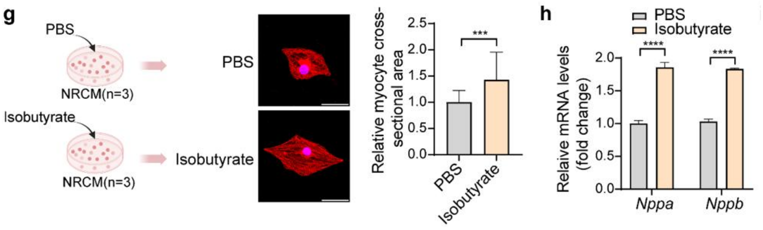

Revised Fig. 3 g, NRCMs were treated with PBS or isobutyrate (5 mM) for 48 h. α-Actinin staining was performed to determine cell size. Representative images and quantification of the sizes of approximately 20 NRCMs in each group are shown (***p<0.001). Scale bar: 30 μm. **h,** NRCMs were treated with PBS or isobutyrate (5 mM) for 48 hours. qRT-PCR was performed to analyse the mRNA expression levels of hypertrophic genes (n=3; ****p<0.0001).

(3) <u>Both isobutyrate treatment and ACAD8 deficiency induced similar transcriptome effects (**Revised Fig. 4a-d**)</u>.

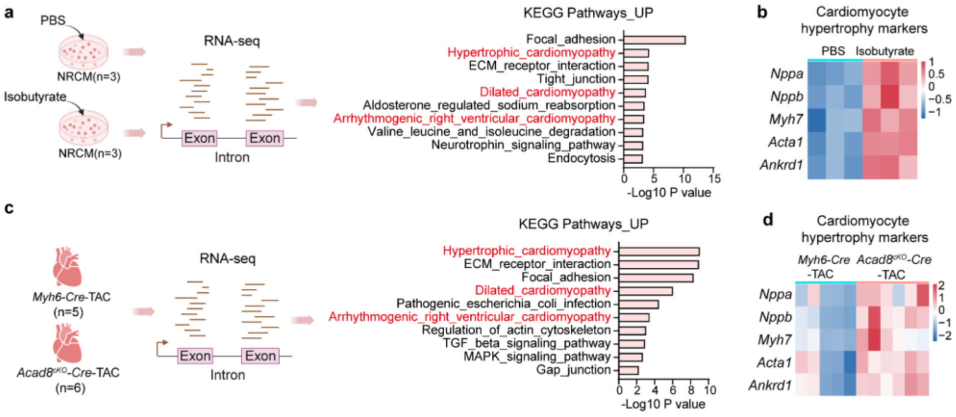

Revised Fig. 4 a, Schematic showing the RNA-seq-based experimental design in which NRCMs were treated with isobutyrate for 36 hours. KEGG functional enrichment analysis of upregulated genes in NRCMs that were treated with isobutyrate. **b,** Heatmaps of the relative expression of the differentially expressed genes associated with cardiomyocyte hypertrophy that were identified in NRCMs that were treated with PBS or isobutyrate. **c,** Schematic showing the RNA-seq-based experimental design for analyzing the left ventricles of *Myh6-Cre*-TAC and *Acad8*^{cKO}-TAC hearts. KEGG functional enrichment analysis of upregulated genes in TAC-operated *Acad8*^{cKO}-TAC hearts. **d,** Heatmaps of the relative expression of the differentially expressed genes associated with cardiomyocyte hypertrophy that were identified in *Myh6-Cre*-TAC and *Acad8*^{cKO}-TAC hearts.

Based on this comment, we added the discussion section (page 12 line 389).

"Our data show that isobutyrate partially recapitulates the effects of ACAD8 deficiency. However, given the complexity of in vivo metabolic networks, we cannot completely exclude additional effects of isobutyrate."

<u>**Comment #2-3**</u>**:** The authors show that ACAD8 downregulation and isobutyrate affect

the transcription of ANP and BNP; there could be a compensatory mechanism of cardiac hypertrophy, but it is unclear how isobutyrate mediates pathological hypertrophy. The molecular mechanism through which isobutyryl-CoA dysregulation leads to pathological hypertrophy remains to be tested and mechanistic experiments to prove the importance of such mechanisms could have been conducted.

Response: Thank you for your comment and suggestion. According to your suggestion, we conducted additional exploratory analyses to validate our initial findings. Histone post-translational modifications may confer distinctive effects on chromatin architecture and its interactions with non-histone proteins, thereby modulating both chromatin structure and transcriptional regulation. Isobutyrate treatment specifically enhanced intracellular isobutyryl-CoA concentrations, which can function as acyl donors to mediate lysine isobutyrylation (Kibu) modifications on histone H3[8], ultimately influencing chromatin accessibility and gene expression profiles.

We used anti-Kibu antibodies for verification and observed elevated isobutyrylation modifications of H3K9 and H3K23 in TAC-operated ACAD8-deficient mouse hearts (**Revised Fig. 3d-e**). Further, *Acad8* knockdown in NRCMs enhanced the isobutyrate-induced isobutyrylation of H3K9 and H3K23 (**Revised Supplementary Fig. 3b-d**).

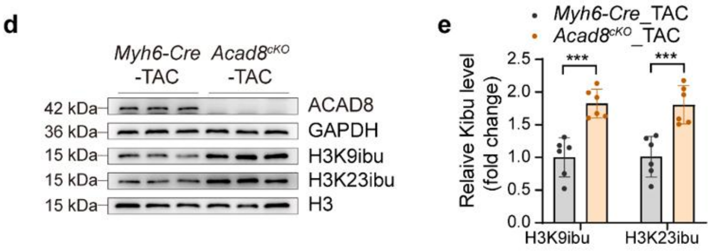

Revised Fig. 3 d, Western blotting and **e,** Quantification of H3K9ibu and H3K23ibu levels in *Myh6-Cre*-TAC and *Acad8*^{cKO}-TAC hearts (n=6; ***$p < 0.001$).

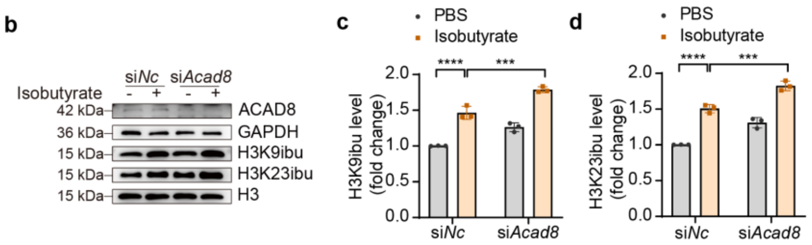

Revised Supplementary Fig. 3 b, Western blotting and **c-d,** Quantification of H3K9ibu and H3K23ibu levels in NRCMs treated with PBS or isobutyrate (5mM) for 36 hours after transfection with si*Nc* or si*Acad8* for 36 hours (n=3; ***$p < 0.001$, ****$p < 0.0001$).

The results in this research demonstrated that both isobutyrate treatment and *Acad8* knockdown significantly enhanced chromatin accessibility at the promoter regions of

hypertrophy markers *Nppa* and *Nppb* (**Revised Fig. 4k-l**). Increased isobutyryl-CoA and isobutyrylation modifications may promote chromatin opening, potentially enhancing TEAD2 binding at the promoters of hypertrophy genes. We initially attempted to perform CUT&Tag experiments using a commercially available TEAD2 antibody, but these efforts proved unsuccessful. Consequently, we overexpressed TEAD2-FLAG in NRCMs and subsequently conducted both CUT&Tag and qRT-PCR assays using an anti-FLAG antibody to verify the binding sites of TEAD2 in the promoter regions of *Nppa* and *Nppb* and found that both isobutyrate treatment and knockdown of *Acad8* were able to promote the binding of TEAD2 in the promoter regions of *Nppa* and *Nppb* (**Revised Fig. 4 f-g**). We next overexpressed TEAD2 in NRCMs to validate its function in cardiac hypertrophy. The results demonstrated that overexpression of TEAD2 promoted the expression of *Nppa* induced by *Acad8* knockdown and isobutyrate treatment (**Revised Fig. 4 h-j**). This result suggests that TEAD2 is able to regulate the expression of cardiac hypertrophy genes.

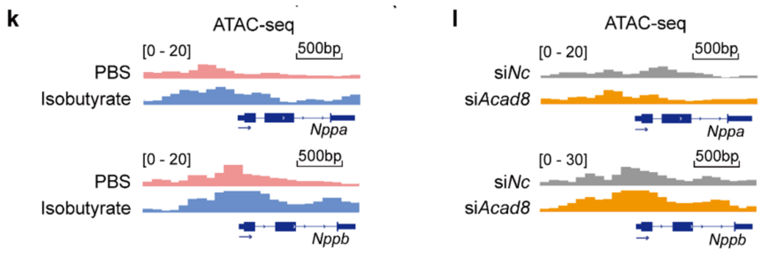

Revised Fig. 4 k, IGV images showing ATAC-seq peaks in NRCMs that were treated with PBS or isobutyrate for 36 hours, aligned across the *Nppa* (top) and *Nppb* (bottom) gene coordinates. **l**, IGV images showing ATAC-seq peaks in NRCMs that were transfected with si*Nc* or si*Acad8* for 48 hours, aligned across the *Nppa* (top) and *Nppb* (bottom) gene coordinates.

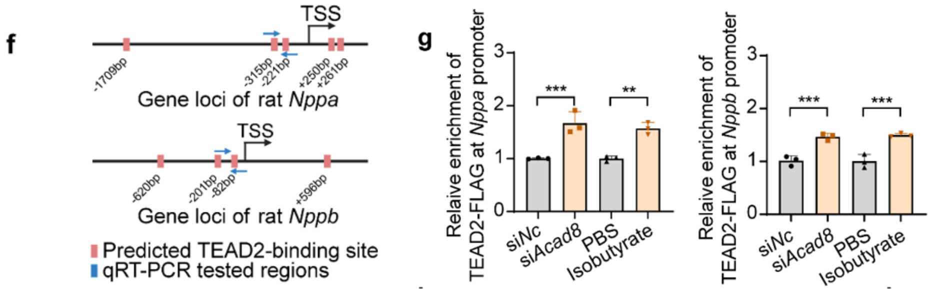

Revised Fig. 4 f, Schematic showing the predicted binding sites and validation regions for TEAD2 in the *Nppa* and *Nppb* promoter regions. The exact positions relative to the TSS are labelled below. **g,** NRCMs were transfected with Ad*TEAD2*, then treated with isobutyrate or transfected with si*Nc* or si*Acad8*, followed by CUT-Tag and qRT-PCR experiments (n=3; $^{**}p<0.01$, $^{***}p<0.001$).

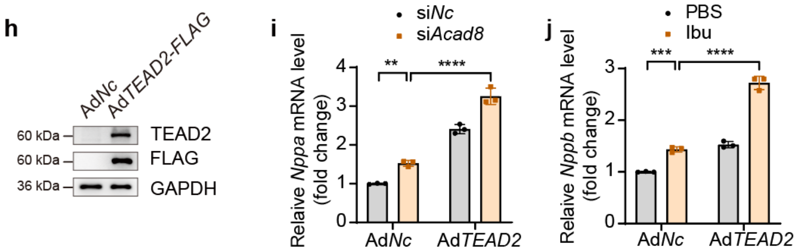

Revised Fig 4 h, Western blotting of the protein expression of TEAD2 in NRCMs transfected with Ad*Nc* or Ad*TEAD2* for 48 hours. **i,** NRCMs were transfected with Ad*Nc* or Ad*TEAD2* for 36 hours after transfection with si*Nc* or si*Acad8* for 36 hours (n=3; $^{**}p < 0.01$, $^{****}p < 0.0001$). **j,** NRCMs were transfected with Ad*Nc* or Ad*TEAD2* for 36 hours after treatment with PBS or isobutyrate for 36 hours (n=3; $^{***}p < 0.001$, $^{****}p < 0.0001$). qRT-PCR was performed to analyse the mRNA expression levels of hypertrophic genes.

Based on this comment, we added the discussion section (page 12 line 403).

"ACAD8 deficiency leads to accumulation of isobutyryl-CoA. The ensuing increase in histone isobutyrylation modifications may alter chromatin accessibility within the promoter regions of hypertrophic genes, potentially influencing the binding of transcription factors such as TEAD2 and thereby modulating gene expression."

Comment #2-4: In Fig. 1fg, The protein level of valine metabolic pathway enzymes in cardiomyocytes should be shown in comparison to another cell type.

Response: Thank you for your comment. According to your suggestion regarding **Revised Fig. 1f-g**, we isolated cardiomyocytes and non-cardiomyocytes from adult mouse hearts and examined the expression of genes involved in valine catabolic pathways in both cell populations. The results demonstrated that the expression of valine catabolism-related genes was significantly higher in cardiomyocytes compared to non-cardiomyocytes (**Revised Fig. 1h**).

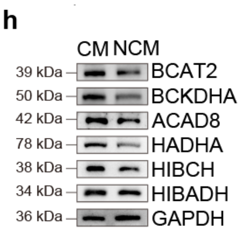

Revised Fig. 1h, Western blotting of the protein expression of valine catabolic genes in cardiomyocytes (CM) and non-cardiomyocytes (NCM) isolated from mouse heart tissue.

A quantitative proteomic analysis study assessed the differences in the protein profiles of cardiomyocytes and fibroblasts isolated from rat hearts and found that cardiomyocytes were significantly enriched in proteins related to energy metabolism[9]. From this, we found higher expression levels of valine catabolic proteins in cardiomyocytes than in fibroblasts (**Data shown to reviewer#2 Fig 1**).

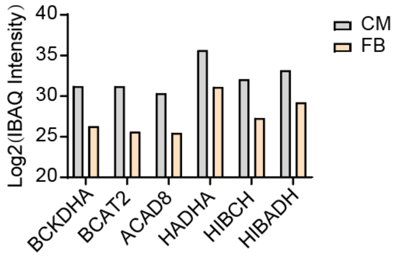

Data shown to reviewer#2 Fig 1, Log2-normalised values for the sum of IBAQ intensities for valine metabolism-related genes in all cardiac cardiomyocyte and fibroblast replicates.

Comment #2-5: In Fig. 1k, the level of ACAD8 in cardiomyocytes should be shown.

Response: Thank you for your comment. According to your suggestion regarding **Revised Fig.1m**, we examined the protein levels of *Acad8* and determined that interfering with *Acad8* can cause a significant reduction in *Acad8* expression levels (**Revised Fig.1l**).

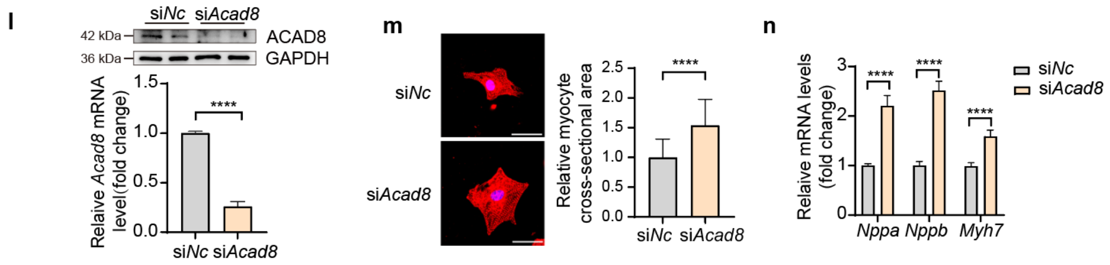

Revised Fig. 1 l, NRCMs were transfected with si*Nc* or si*Acad8* for 48 hours. Western blotting was performed to analyse ACAD8 protein knockdown efficiency (top). qRT-PCR was performed to analyse *Acad8* mRNA knockdown efficiency (n=3; ****p<0.0001) (bottom). **m,** NRCMs were transfected with si*Nc* or si*Acad8* for 48 hours. α-Actinin staining was performed to determine cell size. Representative images (left) and quantification (right) of the sizes of approximately 40 NRCMs in each group are shown (****p<0.0001). Scale bar: 30 μm. **n,** NRCMs were transfected with si*Nc* or si*Acad8* for 48 hours. qRT-PCR was performed to analyse the mRNA expression levels of hypertrophic genes (n=3; ****p<0.0001).

Comment #2-6: In Fig. 2 and 4, the level of cell death should be evaluated.

Response: Thank you for your comment regarding **Revised Fig. 2 and 5**. Cardiac hypertrophy and heart failure progression are frequently accompanied by cell death[10].

To investigate whether *Acad8*^{cKO} and ACAD8-OE in mice affect the level of cell death in the heart, we designed follow-up experiments: (1) TUNEL assay; (2) Cleaved Caspase-3 expression analysis; (3) gene set enrichment analysis (GSEA) on the transcriptomics data. Our results demonstrated that *Acad8*^{cKO} exacerbated TAC-induced cell death, whereas ACAD8-OE attenuated this pathological process.

We first assessed cell death in cardiac tissue using TUNEL assays. The results demonstrated that *Acad8*^{cKO} mice exhibited exacerbated TAC-induced cell death compared to *Myh6-Cre* controls (**Revised Fig. 2k-l**). Following TAC surgery, *Acad8*^{cKO} mice showed significantly elevated levels of cleaved caspase-3 (**Revised Supplementary Fig. 1g-h**) - the canonical marker of programmed cell death[11].

Conversely, ACAD8-OE by AAV9 delivery attenuated TAC-induced cell death relative to AAV9-control mice (**Revised Fig. 5k-l**). Moreover, ACAD8-OE mice displayed significantly reduced cleaved caspase-3 levels following TAC induction (**Revised Supplementary Fig. 6a-b**).

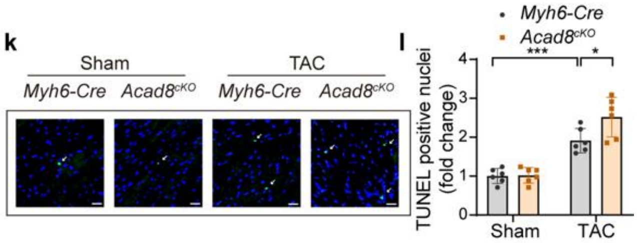

Revised Fig. 2 k, Representative images and **l,** quantitative analysis of TUNEL staining of the hearts of *Myh6-Cre* and *Acad8*^{cKO} mice 4 weeks after Sham or TAC surgery (n=6; *p<0.05, ***p<0.001). Arrows indicate TUNEL-positive cells (green) colocalized with DAPI-stained nuclei (blue). Scale bar: 20μm.

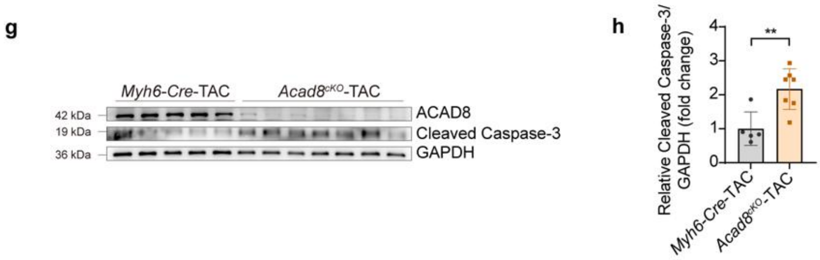

Revised Supplementary Fig. 1 g, Western blotting and **h,** quantification of the protein expression of cleaved Caspase-3 of the hearts of *Myh6-Cre* and *Acad8*^{cKO} mice 4 weeks after Sham or TAC surgery (n=5-7; **p<0.01).

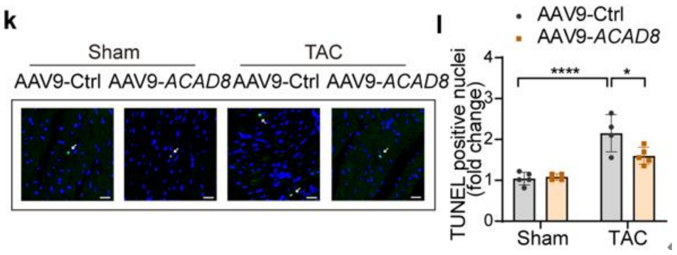

Revised Fig. 5 k, Representative images and **l,** quantitative analysis of TUNEL staining of the mice infected with AAV9-cTnT-Ctrl or AAV9-cTnT-*ACAD8* 4 weeks after Sham or TAC surgery (n=4-5; *p<0.05, ***p<0.001). Arrows indicate TUNEL-positive cells (green) colocalized with DAPI-stained nuclei (blue). Scale bar: 20μm.

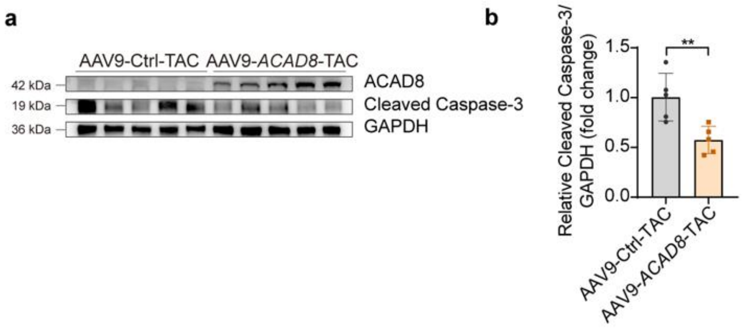

Revised Supplementary Fig. 6 a, Western blotting and **b,** quantification of the protein expression of cleaved Caspase-3 of the mice infected with AAV9-cTnT-Ctrl or AAV9-cTnT-*ACAD8* 4 weeks after Sham or TAC surgery (n=5; **p<0.01).

We further performed GSEA on the transcriptomics data to evaluate cell death pathways. The results showed that apoptosis was significantly increased in *Acad8cKO*-TAC hearts compared to *Myh6-Cre*-TAC hearts (**Data shown to reviewer#2 Fig. 2a**). Conversely, ACAD8-OE hearts showed reduced apoptosis after TAC surgery (**Data shown to reviewer#2 Fig. 2b**).

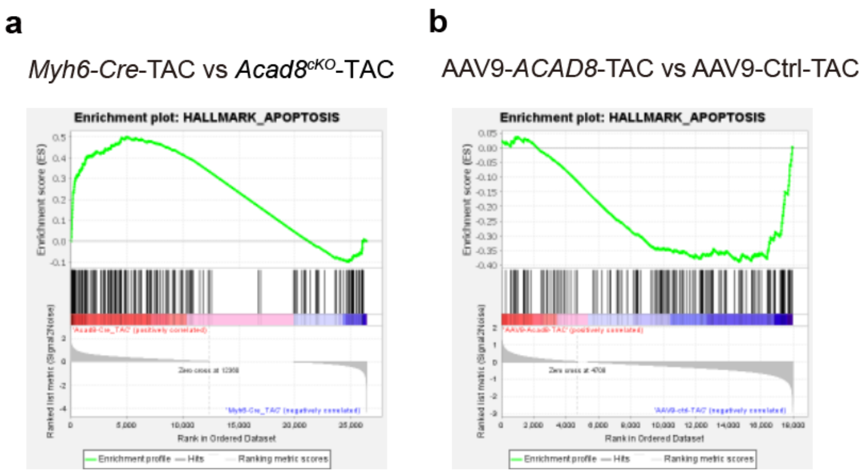

Data shown to reviewer#2 Fig 2 a, Dysregulated apoptotic pathway from Gene set enrichment analysis (GSEA) in *Acad8*^{cKO}-TAC hearts compared to *Myh6-Cre*-TAC hearts (NES: 1.376, p-value: 0.0062). **b,** Dysregulated apoptotic pathway from GSEA in AAV9-*ACAD8*-TAC hearts compared to AAV9-Ctrl-TAC hearts (NES: -1.259, p-value: 0.032).

<u>**Comment #2-7**</u>**:** In Fig. 3mn, the mechanism by which accumulation of isobutyryl-CoA affects the chromatin accessibility should be tested or discussed.

Response: Thank you for your comment regarding **Revised Fig. 4k-l**. According to your suggestion, we conducted additional exploratory analyses to validate our initial findings. <u>Histone post-translational modifications may confer distinctive effects on chromatin architecture and its interactions with non-histone proteins, thereby modulating both chromatin structure and transcriptional regulation.</u> Isobutyrate treatment specifically enhanced intracellular isobutyryl-CoA concentrations, which can function as acyl donors to mediate lysine isobutyrylation modifications on histone H3[8], ultimately influencing chromatin accessibility and gene expression profiles.

We used anti-Kibu antibodies for verification and observed elevated isobutyrylation modifications of H3K9 and H3K23 in TAC-operated ACAD8-deficient mouse hearts (**Revised Fig. 3d-e**). Further, <u>*Acad8* knockdown in NRCMs enhanced the isobutyrate-induced isobutyrylation modifications of H3K9 and H3K23</u> (**Revised Supplementary Fig. 3b-d**).

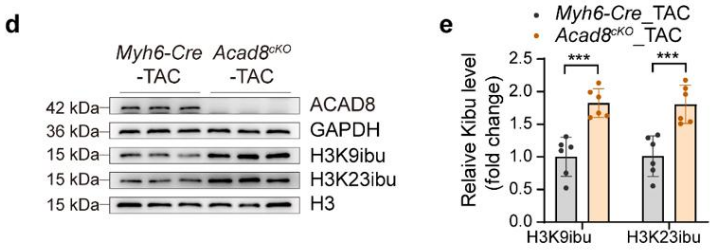

Revised Fig. 3 d, Western blotting and **e,** Quantification of H3k9ibu and H3k23ibu levels in *Myh6-Cre*-TAC and *Acad8*^{cKO}-TAC hearts (n=6; ***$p < 0.001$).

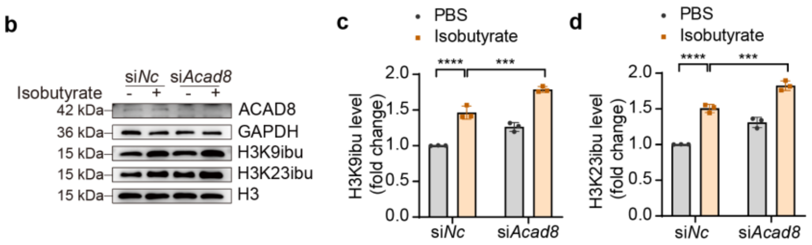

Revised Supplementary Fig. 3 b, Western blotting and **c-d,** Quantification of H3K9ibu and H3K23ibu levels in NRCMs treated with PBS or isobutyrate (5mM) for 36 hours after

transfection with si*Nc* or si*Acad8* for 36 hours (n=3; ***p＜0.001, ****p＜0.0001).

Based on this comment, we added the discussion section (page 11 line 379).

"Our study demonstrated that the isobutyrylation modifications of histone H3K9 and H3K23 were significantly elevated in the hearts of ACAD8-deficient mice subjected to TAC, Similarly, knockdown of *Acad8* in NRCMs promoted isobutyrate-induced isobutyrylation modifications at H3K9 and H3K23. Future studies should further elucidate the regulatory mechanisms of histone isobutyrylation on chromatin remodelling and gene expression.

<u>Comment #2-8</u>: In Fig. 4, the level of endogenous ADAD8 in the heart after TAC with or without the rescued with AAV9-Acad8 should be shown.

Response: Thank you for your comment regarding **Revised Fig.5.** According to your suggestion, the mRNA expression level of endogenous *Acad8* was verified in **Revised Supplementary Fig. 6c**. The results indicate that <u>ACAD8-OE by AAV9 (human *ACAD8*) in TAC hearts causes a rebound of endogenous *Acad8* (murine *Acad8*).</u>

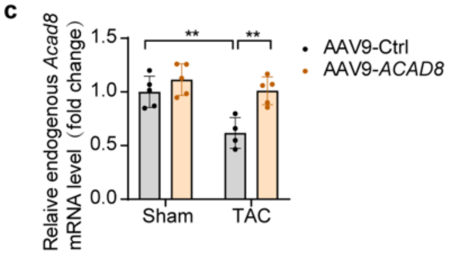

Revised Supplementary Fig. 6 c, qRT-PCR was performed to analyse the mRNA expression level of endogenous *Acad8* in mice infected with AAV9-Ctrl and AAV9-*ACAD8* after Sham or TAC surgery (n=4~5; **p<0.01).

Reference

1 Sabbagha, N. G. *et al.* Alternative splicing in Acad8 resulting a mitochondrial defect and progressive hepatic steatosis in mice. *Pediatric research* **70**, 31-36, doi:10.1203/PDR.0b013e31821b89ee (2011).

2 Haack, T. B. *et al.* Deficiency of ECHS1 causes mitochondrial encephalopathy with cardiac involvement. *Annals of clinical and translational neurology* **2**, 492-509, doi:10.1002/acn3.189 (2015).

3 Wanders, R. J., Duran, M. & Loupatty, F. J. Enzymology of the branched-chain amino acid oxidation disorders: the valine pathway. *Journal of inherited metabolic disease* **35**, 5-12, doi:10.1007/s10545-010-9236-x (2012).

4 Zhuang, D. Y. *et al.* Identification of Six Novel Variants of ACAD8 in Isobutyryl-CoA Dehydrogenase Deficiency With Increased C4 Carnitine Using Tandem Mass Spectrometry and NGS Sequencing. *Frontiers in genetics* **12**, 791869, doi:10.3389/fgene.2021.791869 (2021).

5 Oglesbee, D. *et al.* Development of a newborn screening follow-up algorithm for the diagnosis of isobutyryl-CoA dehydrogenase deficiency. *Genetics in medicine : official journal of the American College of Medical Genetics* **9**, 108-116, doi:10.1097/gim.0b013e31802f78d6 (2007).

6 Cheng, M. L. *et al.* Metabolic disturbances identified in plasma are associated with outcomes in patients with heart failure: diagnostic and prognostic value of metabolomics. *Journal of the American College of Cardiology* **65**, 1509-1520, doi:10.1016/j.jacc.2015.02.018 (2015).

7 Zordoky, B. N. *et al.* Metabolomic fingerprint of heart failure with preserved ejection fraction. *PloS one* **10**, e0124844, doi:10.1371/journal.pone.0124844 (2015).

8 Zhu, Z. *et al.* Identification of lysine isobutyrylation as a new histone modification mark. *Nucleic acids research* **49**, 177-189, doi:10.1093/nar/gkaa1176 (2021).

9 Poulsen, P. C. *et al.* Quantitative proteomics characterization of acutely isolated primary adult rat cardiomyocytes and fibroblasts. *Journal of molecular and cellular cardiology* **143**, 63-70, doi:10.1016/j.yjmcc.2020.04.021 (2020).

10 Sabbah, H. N. Apoptotic cell death in heart failure. *Cardiovascular research* **45**, 704-712, doi:10.1016/s0008-6363(99)00348-x (2000).

11 Eskandari, E. & Eaves, C. J. Paradoxical roles of caspase-3 in regulating cell survival, proliferation, and tumorigenesis. *The Journal of cell biology* **221**, doi:10.1083/jcb.202201159 (2022).

Response to Reviewer #1

In this revised report, authors provided numerous new data to address the main issues raised by the reviewers.

Dear Reviewer #1,

We sincerely thank you for your thorough review and insightful comments on our revised manuscript. Your feedback has been invaluable in strengthening our study. In response to your comments, we have conducted additional experiments and analyses and revised the manuscript accordingly. The major revised parts in the manucsript have been highlighted in yellow. A point-by-point response to your comments is listed below. The figures shown below are labelled in the order as in the manuscript.

Comment #1-1： In one set of experiment, authors measured isobutyryl-CoA in diseased heart. In both human and mouse- post TAC hearts, the metabolite concentrations were not changed. Although authors argued for a lack of dynamic measurements may have masked the differences, the results nevertheless directly challenged the key provision of this report related to ACAD8 deficiency and accumulation of isobutyryl-CoA to drive epigenetic reprogramming and other defects. The elevated blood level of isobutyrylcarnitine in clinical studies can not be properly extrapolated into cardiac defects in isobutyryl-CoA metabolic defects in hearts. This is particularly relevant since isobutrylcarnitine content was not changed in post-TAC mouse heart in either WT or the KO mice. In short, the physiological relevance of isobutyrul-CoA accumulation is a key element of this report and a central data to support the overall working hypothesis. The results would make this hypothesis highly questionable.

Response: Thank you for your insightful comment. We acknowledge that the absence of demonstrable isobutyryl-CoA accumulation in TAC-induced hypertrophic hearts in mouse models represents a limitation of our current study. Here, we would like to propose several potential explanations that help reconcile these observations with our central hypothesis:

1. Direct evidence of isobutyryl-CoA accumulation in *Acad8*-deficient models. Our findings demonstrate that *Acad8* deficiency leads to increased isobutyryl-CoA levels in cardiomyocytes and mouse hearts (**Revised Fig. 3c, f**).

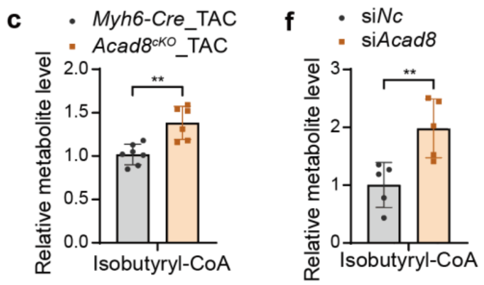

Revised Fig. 3c, Quantification of isobutyryl-CoA levels in *Myh6-Cre*-TAC and *Acad8*^{cKO}-TAC hearts (n=6~7; **p<0.01). **f,** Quantification of isobutyryl-CoA levels in NRCMs transfected with si*Nc* or si*Acad8* for 48 hours (n=5; **p<0.01).

2. ACAD8 deficiency alone leads to transcriptome changes related to hypertrophy in mouse hearts and cardiomyocytes. RNA sequencing revealed that *Acad8*^{cKO} hearts exhibited upregulation of cardiomyopathy-related genes, along with elevated expression of specific cardiac hypertrophy markers under baseline conditions (**Revised Supplementary Fig.**

1i-j), indicating a latent hypertrophic state. A similar pattern was observed in *Acad8*-deficient cardiomyocytes **Fig. R1-1**).

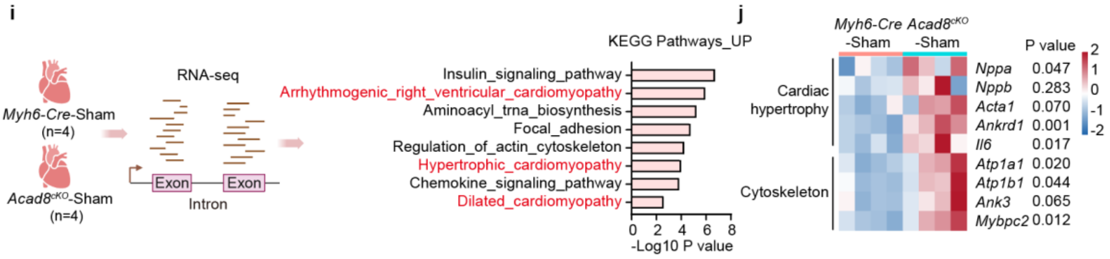

Revised Supplementary Fig. 1 i, KEGG functional enrichment analysis of up-regulated genes in *Acad8^{cKO}*-Sham hearts compared with *Myh6-Cre*-Sham hearts. **j,** Heatmaps of the relative expression of genes associated with cardiac hypertrophy and cytoskeleton in *Myh6-Cre*-Sham and *Acad8^{cKO}*-Sham hearts based on transcriptomic sequencing data.

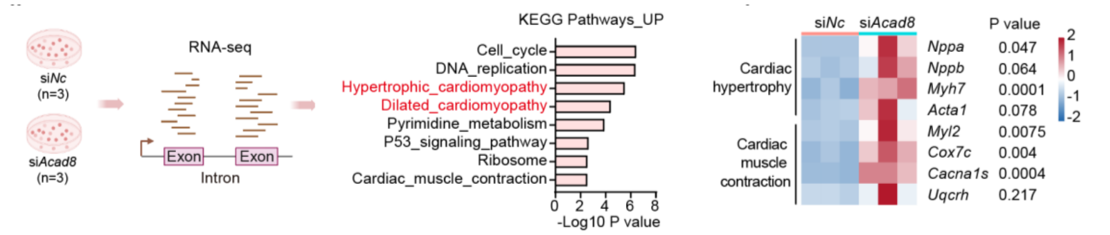

Fig. R1-1, KEGG functional enrichment analysis of up-regulated genes in NRCMs transfected with si*Acad8*. **l,** Heatmaps of the relative expression of genes associated with cardiac hypertrophy and cardiac muscle contraction in NRCMs transfected with si*Acad8* based on transcriptomic sequencing data.

3. ACAD8 deficiency predisposes the heart to exacerbated hypertrophic remodeling under pathological stress. Given that chronic ACAD8 deficiency may cause spontaneous hypertrophy over an extended period, we utilized TAC surgery to accelerate this process. Our findings conclusively show that *Acad8* deficiency predisposes the heart to heightened hypertrophic remodeling under pathological stress (**Revised Fig. 2d-e**). *Acad8*-deficient models better reflect the primary metabolic perturbation, whereas cardiac hypertrophy represents a secondary disease state with complex adaptive responses.

This phenomenon, whereby a genetic perturbation reveals a pronounced phenotype only under stressful conditions, is well established in cardiovascular research. Similar to our findings with ACAD8, cardiac-specific SIRT2 deficiency did not induce spontaneous cardiac hypertrophy in young mice but markedly exacerbated age-associated pathological hypertrophy[1]. From a clinical perspective, mutations downstream of the *CXCL12* gene show a significant correlation with coronary artery disease[2,3]. Supplementing CXCL12 significantly reduced the vulnerability of plaques to instability in *Apoe$^{-/-}$* mice, despite no baseline phenotype being evident[4]. Similarly, consistent with clinical evidence linking GSNOR dysfunction to cardiovascular disease, cardiac-specific GSNOR deficiency does not cause spontaneous cardiac dysfunction, but significantly exacerbates cardiac remodelling induced by pressure overload following TAC surgery[5].

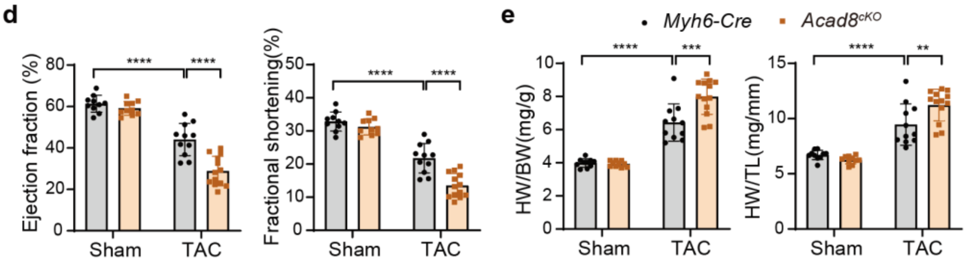

Revised Fig. 2d, Ejection fraction/fractional shortening of *Myh6-Cre* and *Acad8*^{cKO} mice 4 weeks after Sham or TAC surgery (n=10~13; ****p<0.0001). **e,** Heart weight-to-body weight and heart weight-to-tibia length of *Myh6-Cre* and *Acad8*^{cKO} mice 4 weeks after Sham or TAC surgery (n=10~13; **p<0.01, ***p<0.001, ****p<0.0001).

4. The clinical pathological significance of *ACAD8* deficiency. Clinically, the dysregulation of isobutyryl-CoA levels caused by *ACAD8* mutations is associated with cardiomyopathy[6,7]. Our findings demonstrate that *Acad8* deficiency leads to increased isobutyryl-CoA levels and promotes sensitivity to pathological cardiac hypertrophy and related phenotypes upon pressure overload. This study elucidated the previously undefined pathological significance of ACAD8 deficiency and isobutyryl-CoA metabolic dysregulation in human patients.

The absence of significant isobutyryl-CoA accumulation detected in the TAC model is potentially due to several possibilities, such as the widespread downregulation of valine metabolism genes and the potentially differential stress responses across distinct cardiac cell types[8]. As you mentioned, elevated blood levels of isobutylcarnitine in clinical studies cannot be directly attributed to a defect in cardiac isobutyryl-CoA metabolism. Future studies will specifically investigate the levels of isobutyryl-CoA in hypertrophic cardiac tissues to elucidate its clinical significance further.

Overall, the point you raised is crucial to the main findings and conclusions of this study. It prompted us to supplement the relevant experimental data and analysis, and we revised the corresponding sections of the manucsript accordingly, which greatly improved our study. To provide some in-depth insight, we added the discussion section (revised manuscript page 11 line 355).

"*This finding provides key pathological insights for ACAD8 deficiency and isobutyryl-CoA deregulation in human patients. Our results demonstrated that Acad8 deficiency exacerbates TAC-induced hypertrophy by promoting isobutyryl-CoA accumulation. Future studies will specifically investigate the levels of isobutyryl-CoA in hypertrophic cardiac tissues to elucidate its clinical significance further.*"

Comment #1-2： It is clear that cardiac tissue level of isobutyrate-CoA is relatively low. It is not clear if 0.5 mM isobutyrate treatment would lead to what levels of isobutyryl-CoA in NRVM relative to intra-cardiac tissue levels observed in vivo. Metabolic impact is heavily dependent on the level of metabolites. Therefore, a clear demonstration of physiologically relevant concentrations of these metabolites would be critical to establish their biological impact.

Response: Thank you for your comment. According to your suggestions, we conducted further analyses. The concentration of isobutyrate employed in our *in vitro* treatments reasonably approximates physiological conditions based on three layers of evidence: (1) With reference to two published studies, the concentration of isobutyryl-CoA in our data is comparable to the

levels reported in cardiac tissues and cells as measured by mass spectrometry in publications from two independent labs[9,10]. (2) The concentration of isobutyrate supplemented in our experiments resulted in an increase in intracellular isobutyryl-CoA levels that is consistent in both absolute amount and fold-change with values reported in the literature[10]. (3) We did not observe cellular cytotoxicity at the concentration of isobutyrate used for cell treatment.

The detailed response to your concern is described as follows.

1. Endogenous isobutyryl-CoA levels in cardiomyocytes and hearts are at physiologically relevant level. As established by an independent work of Prof. Swietach's group, endogenous isobutyryl-CoA levels in mouse cardiac tissue are ~ 0.5-1 ng/mg tissue (**Fig. R1-2**)[9], while our measurements in NRCMs show baseline levels of ~0.4 ng/mg (**Fig. R1-3**). Thus, the concentrations of endogenous isobutyryl-CoA in cardiomyocytes and hearts in this study are at physiologically relevant level.

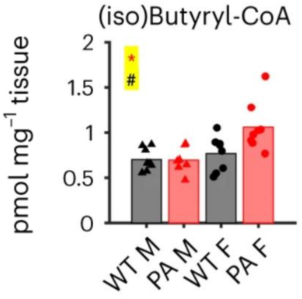

Fig. R1-2 (obtained from Nat Cardiovasc Res. 2023;2(12):1221-1245. Fig2.e). Histograms quantify changes of cardiac acyl-CoA by LC–MS, indicating a significant ($P < 0.05$) effect of genotype (GEN) or interaction between sex and genotype (SEX×GEN).[9]

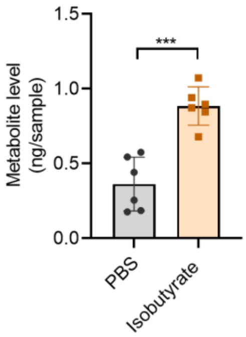

Fig. R1-3. Quantification of isobutyryl-CoA levels in NRCMs that were treated with PBS or Isobutyrate for 48 hours (n=6; ns: not significant, ***p<0.001).

2. The 0.5 mM isobutyrate elevates isobutyryl-CoA to pathophysiological levels. Published studies demonstrate that treatment with 0.5 mM exogenous isobutyrate elevates cellular isobutyryl-CoA levels to approximately 2-fold of baseline endogenous concentrations (**Fig. R1-4**)[10]. This magnitude of increase closely parallels our observation of a 1.5-fold elevation in isobutyryl-CoA content in *Acad8^{cKO}*-TAC hearts compared to controls. The quantitative concordance suggests that 0.5 mM isobutyrate treatment in NRCMs recapitulates the pathophysiological range of isobutyryl-CoA accumulation observed in genetically modified hypertrophic hearts. Importantly, 0.5 mM isobutyrate effectively induces hypertrophic gene expression in NRCMs.

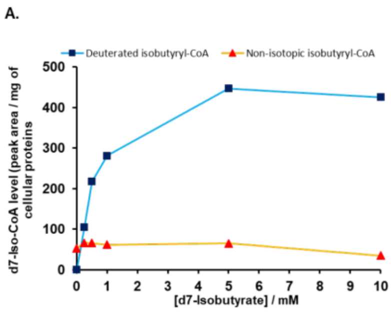

Fig. R1-4 (obtained from Nucleic Acids Res. 2021;49(1):177-189. Fig.S1). Cellular levels of isobutyryl-CoA correlate with isobutyrate. The 293T cells were incubated with different concentrations of d7-isobutyrate. After cell culture and lysis, isobutyryl-CoA from extracts was analyzed by HPLC-MS/MS.

3.The isobutyrate effectively increases hypertrophic responses but not induces cytotoxicity. Previous studies have demonstrated that treatment with 5 mM exogenous isobutyrate induces a saturated elevation of isobutyryl-CoA levels (**Fig. R1-3**)[10]. We employed this higher concentration to investigate broader transcriptional changes, following an established experimental strategy where 6 mM propionate was used to achieve saturated propionyl-CoA levels and maximal transcriptional responses[9]. In this study, we conducted additional experiments demonstrating that isobutyrate concentrations as low as 0.5 mM significantly induced hypertrophy-related gene expression (**Revised Fig. 3i**), while showing no cytotoxicity even at 5 mM (**Revised Supplementary Fig. 3e**).

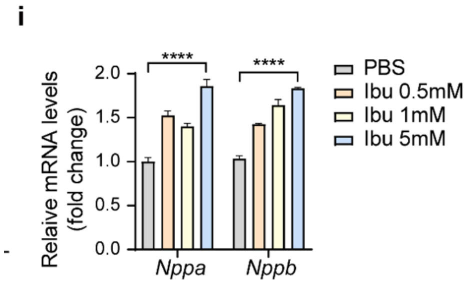

Revised Fig. 3 i, NRCMs were treated with PBS or isobutyrate for 36 hours. qRT-PCR was performed to analyse the mRNA levels of hypertrophic genes (n=3; ****$p < 0.0001$)

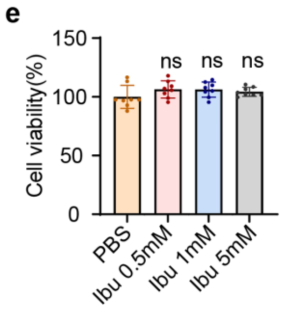

Revised Supplementary Fig. 3 e, CCK-8 assay was performed on NRCMs treated with PBS or isobutyrate at different concentrations for 36 hours (n=8; ns, not significant).

In summary, our data demonstrate that 0.5 mM isobutyrate elevates isobutyryl-CoA to

physiopathological levels consistent with *Acad8*-deficient hearts, effectively modeling the disease state *in vitro* without cytotoxicity. Based on our data and previous studies, the isobutyryl-CoA level induced by exogenous isobutyrate in cardiomyocytes *in vitro* in this study is comparable with those responses in heart tissues *in vivo*[9,10].

Based on these facts, we revised the discussion section (revised manuscript page 11 line 362):

"*Based on our data and previous studies, the isobutyryl-CoA level induced by exogenous isobutyrate in cardiomyocytes in vitro in this study is comparable with those responses in heart tissues in vivo15,18. Given the complexity of in vivo metabolic networks, isobutyrate treatment may partially recapitulate in vivo physiopathological isobutyryl-CoA-mediated effects, but we cannot completely exclude additional effects of isobutyrate.*"

Comment #1-3: The lack of spontaneous hypertrophy from KO in vivo contradicted the observation made in vitro at phenotypic level. From additional in vivo studies at earlier time points post-TAC, it is also clear that contractile dysfunction manifested in parallel with hypertrophy in the KO mice. Therefore, it remains unclear whether cardiac hypertrophy is the primary outcome of Acad8 inactivation.

Response: Thank you for your comment. According to your suggestion, we performed additional experiments and analyses. Firstly, our experimental results and bioinformatic data demonstrate that *Acad8* deficiency directly induces transcriptome reprogramming associated with cardiac hypertrophy at baseline. Second, we fully support your hypothesis that *Acad8* deficiency may also impair cardiac contractile function—a critical finding that warrants further investigation in future studies. Third, we have revised the title of the manuscript to "*ACAD8 deficiency promotes pathological cardiac hypertrophy in response to pressure overload by regulating isobutyryl-CoA levels*", based on the comments from both reviewers.

A detailed response to your concern is shown below.

1. *Acad8* deficiency leads to cardiomyocyte hypertrophy and related transcriptome changes. Our findings in this research demonstrate that *Acad8* knockdown in NRCMs promoted cellular hypertrophy and upregulated the expression of hypertrophy-associated genes (**Revised Fig. 1 l-n**). According to your comment about baseline phenotype, we performed RNA sequencing analysis of NRCMs transfected with si*Acad8*. The results showed that *Acad8* knockdown in NRCMs exhibited upregulation of cardiomyopathy-related genes, along with elevated expression of specific cardiac hypertrophy markers (e.g., *Nppa*, *Myh7*) (**Fig. R1-5**). Additionally, we observed significant enrichment of cardiac muscle contraction pathway among the upregulated genes (**Fig. R1-5**).

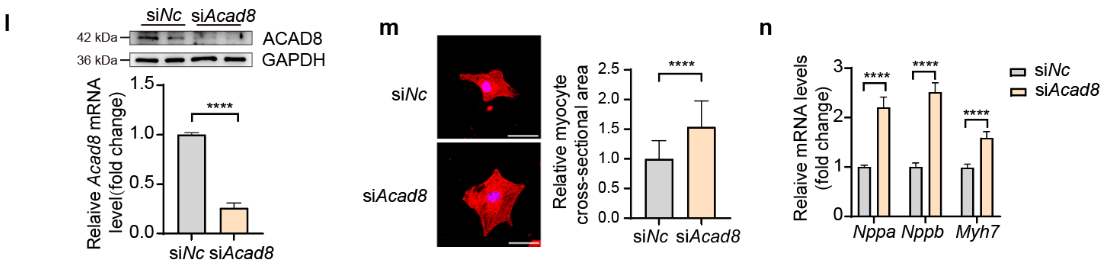

Revised Fig. 1 l, NRCMs were transfected with si*Nc* or si*Acad8* for 48 hours. Western blotting was performed to analyse ACAD8 protein knockdown efficiency (top). qRT-PCR was performed

to analyse *Acad8* mRNA knockdown efficiency (n=3; ****p<0.0001) (bottom). **m,** NRCMs were transfected with si*Nc* or si*Acad8* for 48 hours. α-Actinin staining was performed to determine cell size. Representative images (left) and quantification (right) of the sizes of approximately 40 NRCMs in each group are shown (****p<0.0001). Scale bar: 30 µm. **n,** NRCMs were transfected with si*Nc* or si*Acad8* for 48 hours. qRT-PCR was performed to analyse the mRNA expression levels of hypertrophic genes (n=3; ****p<0.0001).

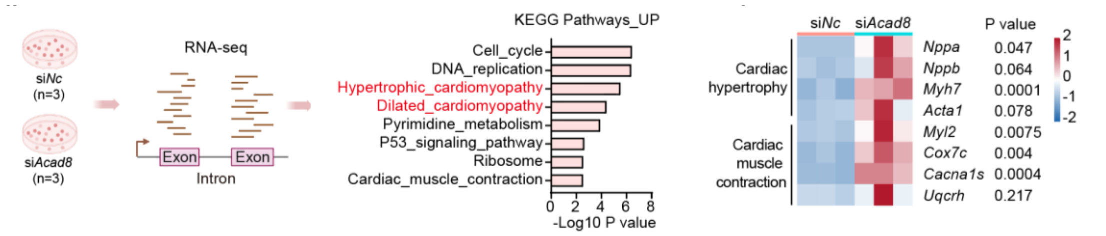

Fig. R1-5, KEGG functional enrichment analysis of up-regulated genes in NRCMs transfected with si*Acad8*. **l,** Heatmaps of the relative expression of genes associated with cardiac hypertrophy and cardiac muscle contraction in NRCMs transfected with si*Acad8* based on transcriptomic sequencing data.

2. *Acad8* deficiency in mouse hearts leads to transcriptome changes related to cardiac hypertrophy. Consistent with the cellular transcriptomic findings, RNA sequencing revealed that *Acad8*^{cKO} hearts under baseline conditions (eight weeks post-tamoxifen injection) exhibited upregulation of cardiomyopathy-related genes, along with elevated expression of specific cardiac hypertrophy markers (e.g., *Nppa*, *Ankrd1*) (**Revised Supplementary Fig. 1i-j**). Furthermore, we also observed an upregulation in the expression of genes related to myocardial contraction (**Revised Supplementary Fig. 1i-j**). These transcriptome changes may reveal that cardiomyocyte-specific *Acad8* knockout mice are more susceptible to developing cardiac hypertrophy when exposed to external stimuli, although these mice did not exhibit spontaneous cardiac hypertrophy within eight weeks of tamoxifen injection.

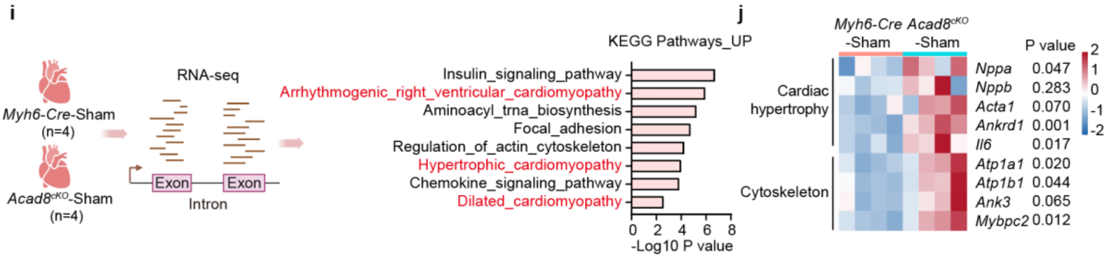

Revised Supplementary Fig. 1 i, KEGG functional enrichment analysis of up-regulated genes in *Acad8*^{cKO}-Sham hearts compared with *Myh6-Cre*-Sham hearts. **j,** Heatmaps of the relative expression of genes associated with cardiac hypertrophy and cytoskeleton in *Myh6-Cre*-Sham and *Acad8*^{cKO}-Sham hearts based on transcriptomic sequencing data.

Collectively, these findings established that *Acad8* deficiency promotes pathological cardiac hypertrophy. At the molecular level, the lack of *Acad8* drives significant upregulation of both hypertrophy-related and cardiac contraction-associated genes. While no overt cardiac phenotype was observed under non-stressed conditions during the study period, *Acad8*^{cKO} hearts exhibited exacerbated pathological hypertrophy accompanied by contractile dysfunction following TAC stimulation. This study primarily investigates the role of ACAD8 deficiency in cardiac hypertrophy. It is noteworthy that ACAD8 may also directly regulate contractile function, which remains to be fully investigated in further studies.

Based on this comment, we added the result section (revised manuscript page 11 line 345)

and the discussion section (revised manuscript page 12 line 399):

"*While no overt cardiac phenotype was observed under non-stressed conditions during the study period (eight weeks post-tamoxifen injection), Acad8 deficiency upregulated hypertrophy-related genes at baseline and increased susceptibility to pathological cardiac remodelling when exposed to pressure overload. Whether long-term Acad8 deficiency could lead to spontaneous cardiac hypertrophy warrants further investigation.*"

"*It is noteworthy that the deficiency of Acad8 not only promotes cardiac hypertrophy but also upregulates genes associated with cardiac dysfunction and impairs contractile performance, thereby exacerbating the pathological cardiac hypertrophy.*"

Comment #1-4: Adding H3 Kibu modification data is exciting and important. However, changes in the total level was not sufficient to support its biological function associated with targeted gene expression. If authors can demonstrate modified H3K9 and K3K23 directly decorate hypertrophic gene promoters in association with TAC induced hypertrophy, it would be a major finding and a significant step forward in the field.

Response: Thank you for your comment. According to your suggestion, we performed CUT&Tag-qPCR for further validation. The results indicate that both isobutyrate treatment and *Acad8* knockdown promoted the enrichment of H3K9ibu and H3K23ibu at the promoter region of the hypertrophy-related gene *Nppa* (**Revised Fig. 4 m-o and Revised Supplementary Fig. 6a-d**). This finding directly links the alterations in H3K9ibu/H3K23ibu modifications to the transcriptional control of hypertrophy-related genes, offering mechanistic support for the role of ACAD8 deficiency in driving pathological cardiac hypertrophy.

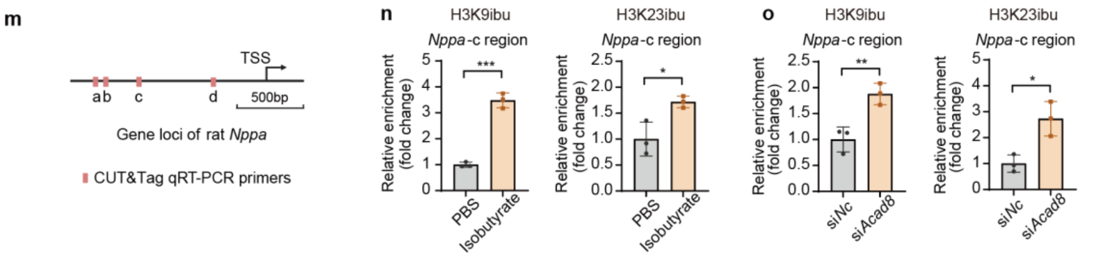

Revised Fig. 4 m, Schematic showing the validation sites for isobutyrylation modification levels in the *Nppa* promoter region. *Nppa*-a region: -882bp~-773bp; *Nppa*-b region: -838bp~-717bp; *Nppa*-c region: -735bp~-608bp; *Nppa*-d region: -317bp~-198bp. **n,** NRCMs were treated with PBS or isobutyrate for 48 hours. Subsequently, CUT&Tag assays were performed using antibodies against histone H3K9ibu or H3K23ibu, followed by qRT-PCR experiments (n=3; *p<0.05, ***p<0.001). **o,** NRCMs were transfected with si*Nc* or si*Acad8* for 48 hours. Subsequently, CUT&Tag assays were performed using antibodies against histone H3K9ibu or H3K23ibu, followed by qRT-PCR experiments (n=3; *p<0.05, **p<0.01).

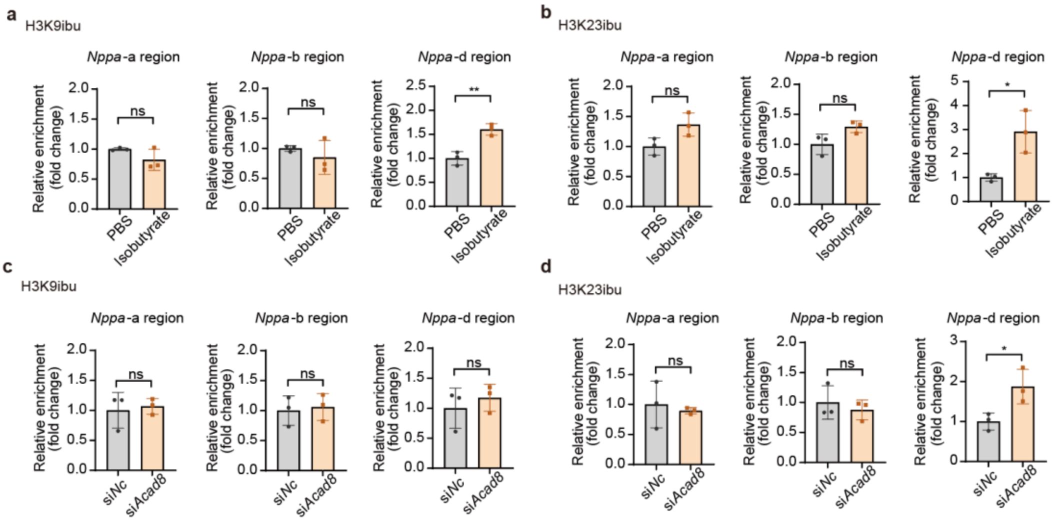

Revised Supplementary Fig. 6 a-b, NRCMs were treated with PBS or isobutyrate for 48 hours. Subsequently, CUT&Tag assays were performed using antibodies against histone H3K9ibu or H3K23ibu, followed by qRT-PCR experiments (n=3; ns, not significant, *p<0.05, **p<0.01). **c-d,** NRCMs were transfected with si*Nc* or si*Acad8* for 48 hours. Subsequently, CUT&Tag assays were performed using antibodies against histone H3K9ibu or H3K23ibu, followed by qRT-PCR experiments (n=3; ns, not significant, *p<0.05).

The key revisions are summarized as follows:

1. Regarding the changes in isobutyryl-CoA levels in TAC-challenged hearts. (1) Our findings demonstrate that *Acad8* deficiency leads to increased isobutyryl-CoA levels and promotes sensitivity to pathological cardiac hypertrophy and related phenotypes upon pressure overload. (2) We re-emphasize that this study primarily focuses on the pathological significance of *Acad8* deficiency and its associated metabolic dysregulation. A discussion has been included in the manuscript to provide appropriate context for the findings.

2. Regarding the isobutyrate concentration used *in vitro*. (1) We performed additional comparative analyses with published data to demonstrate that 0.5 mM isobutyrate treatment elevates cellular isobutyryl-CoA to pathophysiological levels, consistent with observations in *Acad8*-deficient hearts. (2) Cytotoxicity assays confirmed the safety of this concentration.

3. Regarding the relationship between *Acad8* deficiency and cardiac hypertrophy. (1) We have supplemented our analysis with results demonstrating that *Acad8* deficiency upregulates hypertrophy-related genes and cardiac muscle contraction pathway genes even under baseline conditions. (2) We added a discussion on the potential mechanisms masking spontaneous hypertrophy and the exacerbated response under stress.

4. Regarding the functional validation of H3K9ibu and H3K23ibu modifications. We performed CUT&Tag-qPCR assays confirming enriched H3K9ibu and H3K23ibu occupancy at the promoter of the hypertrophy-related gene *Nppa* upon isobutyrate treatment or *Acad8* knockdown, directly linking these modifications to transcriptional regulation.

5. Change of conclusion and manuscript title. Based on your comments above, we have revised the conclusion and changed the title of the manuscript to "*ACAD8 deficiency promotes pathological cardiac hypertrophy in response to pressure overload by regulating isobutyryl-CoA levels*", to better reflect the key findings. We believe these revisions have significantly improved the quality and clarity of our work. Thank you once again for your constructive input.

We believe these revisions have addressed your concerns and more accurately contextualized our findings. Thank you again for your kind suggestion, it imporved our manuscript greatly.

Reference

1 Tang, X. et al. SIRT2 Acts as a Cardioprotective Deacetylase in Pathological Cardiac Hypertrophy. Circulation 136, 2051-2067, doi:10.1161/circulationaha.117.028728 (2017).

2 Kiechl, S. et al. Coronary artery disease-related genetic variant on chromosome 10q11 is associated with carotid intima-media thickness and atherosclerosis. Arteriosclerosis, thrombosis, and vascular biology 30, 2678-2683, doi:10.1161/atvbaha.110.213785 (2010).

3 Mehta, N. N. et al. The novel atherosclerosis locus at 10q11 regulates plasma CXCL12 levels. European heart journal 32, 963-971, doi:10.1093/eurheartj/ehr091 (2011).

4 Akhtar, S., Gremse, F., Kiessling, F., Weber, C. & Schober, A. CXCL12 promotes the stabilization of atherosclerotic lesions mediated by smooth muscle progenitor cells in Apoe-deficient mice. Arteriosclerosis, thrombosis, and vascular biology 33, 679-686, doi:10.1161/atvbaha.112.301162 (2013).

5 Tang, X. et al. Mitochondrial GSNOR Alleviates Cardiac Dysfunction via ANT1 Denitrosylation. Circulation research 133, 220-236, doi:10.1161/circresaha.123.322654 (2023).

6 Oglesbee, D. et al. Development of a newborn screening follow-up algorithm for the diagnosis of isobutyryl-CoA dehydrogenase deficiency. Genetics in medicine : official journal of the American College of Medical Genetics 9, 108-116, doi:10.1097/gim.0b013e31802f78d6 (2007).

7 Zhuang, D. Y. et al. Identification of Six Novel Variants of ACAD8 in Isobutyryl-CoA Dehydrogenase Deficiency With Increased C4 Carnitine Using Tandem Mass Spectrometry and NGS Sequencing. Frontiers in genetics 12, 791869, doi:10.3389/fgene.2021.791869 (2021).

8 Sun, H. et al. Catabolic Defect of Branched-Chain Amino Acids Promotes Heart Failure. Circulation 133, 2038-2049, doi:10.1161/circulationaha.115.020226 (2016).

9 Park, K. C. et al. Disrupted propionate metabolism evokes transcriptional changes in the heart by increasing histone acetylation and propionylation. Nature cardiovascular research 2, 1221-1245, doi:10.1038/s44161-023-00365-0 (2023).

10 Zhu, Z. et al. Identification of lysine isobutyrylation as a new histone modification mark. Nucleic acids research 49, 177-189, doi:10.1093/nar/gkaa1176 (2021).

Response to Reviewer #2

Dear Reviewer #2,

We greatly appreciate your thoughtful comments and suggestions, which have helped us refine our conclusions and enhance the overall impact of our study. In response to your suggestions, we have conducted further experiments and analyses. The major revised parts in the manuscript have been highlighted in yellow. A point-by-point response to the comments is listed below. The figures shown below are labelled in the order as in the manuscript.

Comment #2-1: The authors show in Supplementary Figure 2 that isobutyrylcarnitine is elevated in Acad8cKO mice at baseline (sham). However TAC fails to increase the level of isobutyrylcarnitine or isobutyryl-CoA in these mice. Obviously the isobutyryl-CoA levels do not correlate with cardiac phenotype. This is puzzling and would require modifications in the conclusion. This reviewer is not convinced by the authors' conclusions. Does the baseline phenotype condition the heart in a way more susceptible to stress?

Response: Thank you for your insightful comment. We completely understand your concern. Here, we would like to propose several potential explanations that help reconcile these observations with our central hypothesis:

1. Direct evidence of isobutyryl-CoA accumulation in *Acad8*-deficient models. Our findings demonstrate that *Acad8* deficiency leads to increased isobutyryl-CoA levels in cardiomyocytes and mouse hearts (**Revised Fig. 3c, f**).

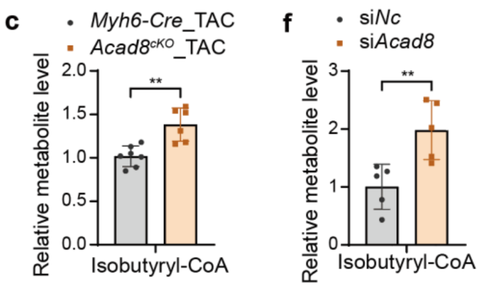

Revised Fig. 3c, Quantification of isobutyryl-CoA levels in *Myh6-Cre*-TAC and *Acad8^{cKO}*-TAC hearts (n=6~7; **p<0.01). **f,** Quantification of isobutyryl-CoA levels in NRCMs transfected with si*Nc* or si*Acad8* for 48 hours (n=5; **p<0.01).

2. *Acad8* deficiency alone leads to transcriptome changes related to hypertrophy in cardiomyocytes and mouse hearts. Based on your comment regarding the baseline phenotype, we conducted further experiments and analysis. Our findings demonstrat that *Acad8* knockdown in NRCMs promoted cellular hypertrophy and upregulated the expression of hypertrophy-associated genes (**Revised Fig. 1 l-n**). RNA sequencing revealed that *Acad8* knockdown in NRCMs exhibited upregulation of cardiomyopathy-related genes, along with elevated expression of specific cardiac hypertrophy markers (e.g., *Nppa*, *Myh7*) (**Fig. R2-1**).

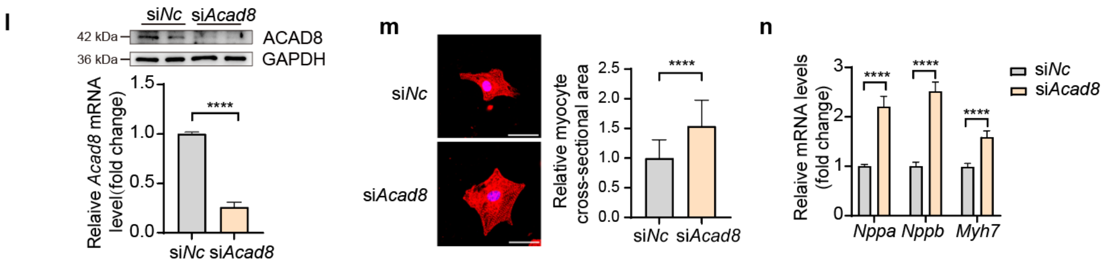

Revised Fig. 1 l, NRCMs were transfected with si*Nc* or si*Acad8* for 48 hours. Western blotting was performed to analyse ACAD8 protein knockdown efficiency (top). qRT-PCR was performed to analyse *Acad8* mRNA knockdown efficiency (n=3; ****p<0.0001) (bottom). **m,** NRCMs were transfected with si*Nc* or si*Acad8* for 48 hours. α-Actinin staining was performed to determine cell size. Representative images (left) and quantification (right) of the sizes of approximately 40 NRCMs in each group are shown (****p<0.0001). Scale bar: 30 μm. **n,** NRCMs were transfected with si*Nc* or si*Acad8* for 48 hours. qRT-PCR was performed to analyse the mRNA expression levels of hypertrophic genes (n=3; ****p<0.0001).

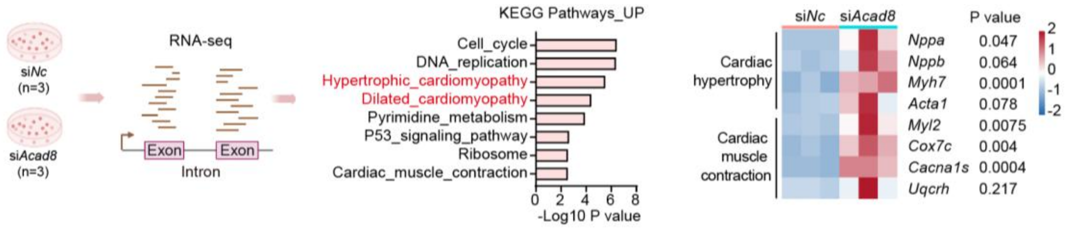

Fig. R2-1, KEGG functional enrichment analysis of up-regulated genes in NRCMs transfected with si*Acad8*. **l,** Heatmaps of the relative expression of genes associated with cardiac hypertrophy and cardiac muscle contraction in NRCMs transfected with si*Acad8* based on transcriptomic sequencing data.

Consistent with the transcriptomic findings in cells, RNA sequencing revealed that $Acad8^{cKO}$ hearts exhibited upregulation of cardiomyopathy-related genes, along with elevated expression of specific cardiac hypertrophy markers_(e.g., *Nppa*, *Ankrd1*) under baseline conditions (**Revised Supplementary Fig. 1i-j**), indicating a latent hypertrophic state.

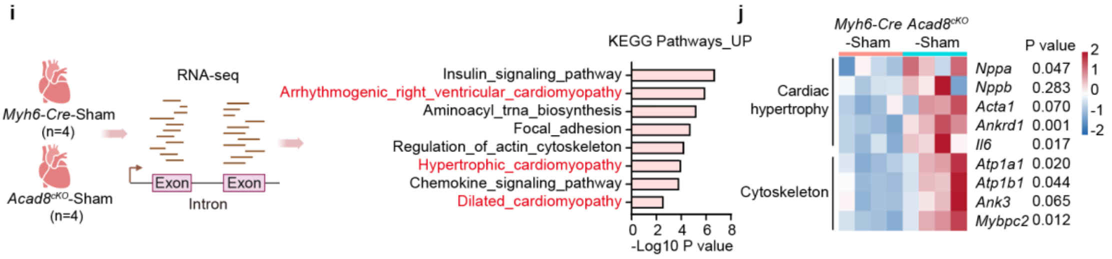

Revised Supplementary Fig. 1 i, KEGG functional enrichment analysis of up-regulated genes in $Acad8^{cKO}$-Sham hearts compared with *Myh6-Cre*-Sham hearts. **j,** Heatmaps of the relative expression of genes associated with cardiac hypertrophy and cytoskeleton in *Myh6-Cre*-Sham and $Acad8^{cKO}$-Sham hearts based on transcriptomic sequencing data.

3. ACAD8 deficiency predisposes the heart to exacerbated hypertrophic remodeling under pathological stress. Given that chronic ACAD8 deficiency may cause spontaneous hypertrophy over an extended period, we utilized TAC surgery to accelerate this process. Our findings conclusively show that *Acad8* deficiency predisposes the heart to heightened hypertrophic remodeling under pathological stress (**Revised Fig. 2d-e**). *Acad8*-deficient models better reflect the primary metabolic perturbation, whereas cardiac hypertrophy represents a secondary disease state with complex adaptive responses.

This phenomenon, whereby a genetic perturbation reveals a pronounced phenotype only under stressful conditions, is well established in cardiovascular research. Similar to our findings with ACAD8, cardiac-specific SIRT2 deficiency did not induce spontaneous cardiac hypertrophy in young mice but markedly exacerbated age-associated pathological hypertrophy[1]. From a clinical perspective, mutations downstream of the *CXCL12* gene show a significant correlation with coronary artery disease[2,3]. Supplementing CXCL12 significantly reduced the vulnerability

of plaques to instability in *Apoe*$^{-/-}$ mice, despite no baseline phenotype being evident[4]. Similarly, consistent with clinical evidence linking GSNOR dysfunction to cardiovascular disease, cardiac-specific GSNOR deficiency does not cause spontaneous cardiac dysfunction, but significantly exacerbates cardiac remodelling induced by pressure overload following TAC surgery[5].

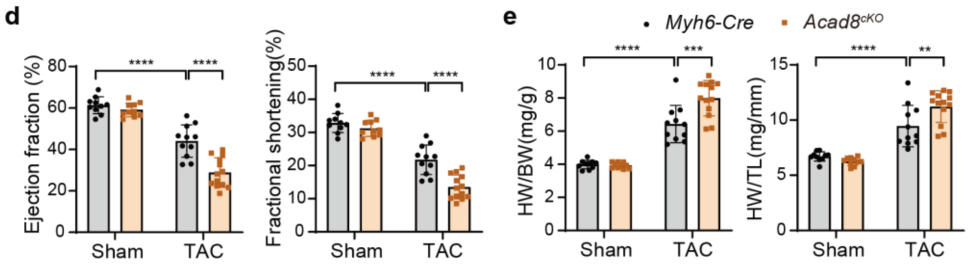

Revised Fig. 2d, Ejection fraction/fractional shortening of *Myh6-Cre* and *Acad8*^{cKO} mice 4 weeks after Sham or TAC surgery (n=10~13; ****p<0.0001). **e,** Heart weight-to-body weight and heart weight-to-tibia length of *Myh6-Cre* and *Acad8*^{cKO} mice 4 weeks after Sham or TAC surgery (n=10~13; **p<0.01, ***p<0.001, ****p<0.0001).

4. The clinical pathological significance of *ACAD8* deficiency. Clinically, the dysregulation of isobutyryl-CoA levels caused by *ACAD8* mutations is associated with cardiomyopathy[6,7]. Our findings demonstrate that *Acad8* deficiency leads to increased isobutyryl-CoA levels and promotes sensitivity to pathological cardiac hypertrophy and related phenotypes upon pressure overload. This study elucidated the previously undefined pathological significance of ACAD8 deficiency and isobutyryl-CoA metabolic dysregulation in human patients.

Collectively, these findings confirm that *Acad8* deficiency promotes sensitivity to pathological cardiac hypertrophy. While no overt phenotype was observed at baseline, significant upregulation of hypertrophy-related genes indicates a pre-pathological state. TAC stress activates latent hypertrophic programs and exacerbates cardiac remodeling in *Acad8*-deficient mice. Thus, *Acad8* deficiency creates a molecular state that predisposes the heart to stress-induced pathological hypertrophy. Based on these facts, we have revised the manuscript title to "*ACAD8 deficiency promotes pathological cardiac hypertrophy in response to pressure overload by regulating isobutyryl-CoA levels*".

Comment #2-2: Since the enhancement of pathological hypertrophy is seen only after pressure overload, the authors should modify the title of this work as "ACAD8 deficiency promotes pathological cardiac hypertrophy in response to pressure overload by regulating......."

Response: Thank you for this comment. According to your suggestion, we have revised the manuscript title to "**ACAD8 deficiency promotes pathological cardiac hypertrophy in response to pressure overload by regulating isobutyryl-CoA levels**". Besides, we also modified some conclusions based on your suggestions and the key findings. We have further emphasized the role of ACAD8 deficiency increased the sensitivity to stress-induced pathological hypertrophy in the discussion sections (revised manuscript page 11 line 346).

"*While no overt cardiac phenotype was observed under non-stressed conditions during the study period (eight weeks post-tamoxifen injection), Acad8 deficiency upregulated hypertrophy-related genes at baseline and increased susceptibility to pathological cardiac remodelling when*

exposed to pressure overload. Whether long-term Acad8 deficiency could lead to spontaneous cardiac hypertrophy warrants further investigation."

The key improvements are summarized below:

1. Regarding the isobutyryl-CoA levels and cardiac phenotype in *Acad8*-deficiency individuals. (1) We re-emphasize that *Acad8* deficiency increases isobutyryl-CoA levels and increased sensitivity to pathological cardiac hypertrophy in mice. This finding aligns with the cardiomyopathy observed in patients with ACAD8 mutations. (2) Transcriptomic analyses demonstrate that *Acad8* deficiency alone induces the expression of hypertrophy-associated genes in both cardiomyocytes and mouse hearts, establishing a latent hypertrophic state. A discussion of these limitations has been included in the manuscript to provide appropriate context for the findings.

2. Regarding the title modification. Based on your suggestion, we have revised the title to: "ACAD8 deficiency promotes pathological cardiac hypertrophy in response to pressure overload by regulating isobutyryl-CoA levels." Furthermore, we have emphasized the critical role of stress induction more prominently and modified some conclusions throughout the manuscript.

We believe these revisions have addressed your concerns and more accurately contextualized our findings. Thank you again for your valuable feedback and contributions to improving our manuscript.

Reference

1 Tang, X. et al. SIRT2 Acts as a Cardioprotective Deacetylase in Pathological Cardiac Hypertrophy. Circulation 136, 2051-2067, doi:10.1161/circulationaha.117.028728 (2017).

2 Kiechl, S. et al. Coronary artery disease-related genetic variant on chromosome 10q11 is associated with carotid intima-media thickness and atherosclerosis. Arteriosclerosis, thrombosis, and vascular biology 30, 2678-2683, doi:10.1161/atvbaha.110.213785 (2010).

3 Mehta, N. N. et al. The novel atherosclerosis locus at 10q11 regulates plasma CXCL12 levels. European heart journal 32, 963-971, doi:10.1093/eurheartj/ehr091 (2011).

4 Akhtar, S., Gremse, F., Kiessling, F., Weber, C. & Schober, A. CXCL12 promotes the stabilization of atherosclerotic lesions mediated by smooth muscle progenitor cells in Apoe-deficient mice. Arteriosclerosis, thrombosis, and vascular biology 33, 679-686, doi:10.1161/atvbaha.112.301162 (2013).

5 Tang, X. et al. Mitochondrial GSNOR Alleviates Cardiac Dysfunction via ANT1 Denitrosylation. Circulation research 133, 220-236, doi:10.1161/circresaha.123.322654 (2023).

6 Oglesbee, D. *et al.* Development of a newborn screening follow-up algorithm for the diagnosis of isobutyryl-CoA dehydrogenase deficiency. *Genet Med* 9, 108-116 (2007).

7 Zhuang, D. Y. *et al.* Identification of Six Novel Variants of ACAD8 in Isobutyryl-CoA Dehydrogenase Deficiency With Increased C4 Carnitine Using Tandem Mass Spectrometry and NGS Sequencing. *Front Genet* 12, 791869 (2021).

Reviewer #1 (Remarks to the Author):

In this revision, authors provided several additional analysis to support the validity of their central hypothesis.

Dear Reviewer,

We sincerely thank you for your thorough review and insightful comments on our revised manuscript. Your feedback has been invaluable in strengthening our study. In response to your comments, we have conducted additional experiments and analyses and revised the manuscript accordingly. The major revised parts have been highlighted in yellow. A point-by-point response to your comments is listed below. The figures shown below are labelled in the order as in the manuscript.

Comment #1: The missing key data supporting the change of isobutyryl-CoA metabolite in the setting of heart failure remains a source of concern as this is the most novel aspect of this study. Although over-expression offered interesting and potentially important support for its translational relevance.

Response: Thank you for your insightful comment. We fully appreciate your concerns regarding the need for more direct evidence linking isobutyryl-CoA to cardiac hypertrophy *in vivo*. Following your suggestion, we did additional experiments to address such questions. We also noticed the editor's suggestion to consider performing valine-loading experiments in normal *vs.* heart failure mice, along with the guidance that if such experiments are not feasible, we should provide a clear explanation. In keeping with both your comment and the editor's suggestion, we have conducted the following complementary studies and respond as follows:

1) Possible explanation for isobutyryl-CoA level in failing hearts: We acknowledge that total isobutyryl-CoA levels did not change significantly in TAC-induced hypertrophied mice hearts at four weeks post-surgery. Beyond the mechanisms discussed in the manuscript, an alternative possibility is that isobutyryl-CoA may directly modify a large number of cardiac structural proteins, thereby affecting contractile function. Such widespread protein modification could deplete the metabolic pool, preventing the accumulation of "free" isobutyryl-CoA. Similar mechanisms have been reported for other short-chain acylations (e.g., crotonylation), which we also observed to extensively modify structural proteins[1,2]. Therefore, we examined the levels of pan-isobutyryllysine (pan-Kibu) and found that both non-histone and histone H3 isobutyrylation were elevated in TAC-operated mouse hearts (**Revised Supplementary Figure 2c-d**). The increased demand for histone isobutyrylation during hypertrophy could consume the "free" isobutyryl-CoA pool, explaining the elevated modification levels without a net increase in its total metabolite concentration.

Overall, we observed that *Acad8* deficiency elevates isobutyryl-CoA levels in TAC hearts and found a significant increase in histone H3 isobutyrylation. In TAC hearts, although total isobutyryl-CoA did not rise, global protein isobutyrylation levels (including histone and non-histone) were enhanced, suggesting that the functional output of this metabolite—through widespread protein modification—better reflects its pathological role. Therefore, we have refined our focus from static metabolite levels to its role as a substrate for protein modification

its role as a substrate for protein modification. Moreover, the supporting experimental evidence in this manuscript indicates that histone isobutyrylation constitutes a key functional link between metabolic alteration and pathological phenotype. Accordingly, we change the words "isobutyryl-CoA levels" to "histone isobutyrylation" and the manuscript title will be "*ACAD8 deficiency promotes pathological cardiac hypertrophy in response to pressure overload by regulating histone isobutyrylation*".

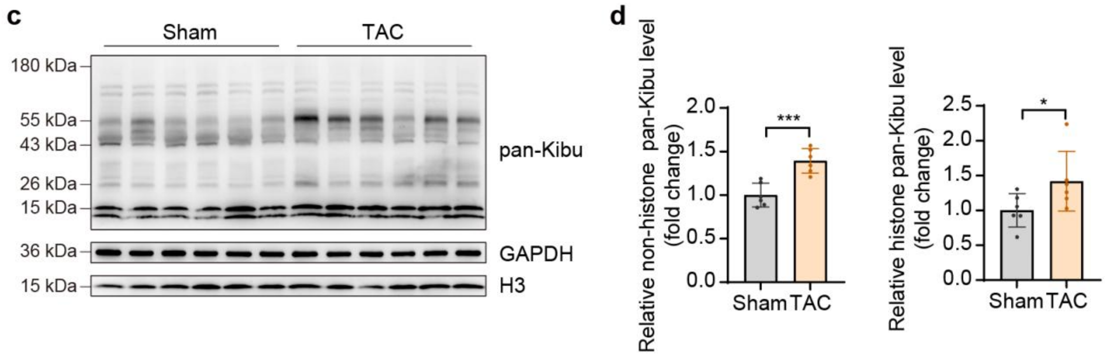

Revised Supplementary Figure 2. c, Western blotting and **d,** Quantification of non-histone and histone Kibu levels in Sham-operated and TAC-induced hypertrophic hearts for 4 weeks (n=6; **p*<0.05, ****p*<0.001).

2) Valine loading elevates isobutyryl-CoA and promotes cardiomyocyte hypertrophy: We have conducted dose-response experiments in cultured cardiomyocytes to directly investigate the valine–isobutyryl-CoA axis. NRCMs were cultured in a valine-depleted medium and then treated with increasing concentrations of valine. The results showed that NRCMs in media supplemented with increasing concentrations of valine resulted in a dose-dependent upregulation of key hypertrophy-related genes, including *Nppa*, *Nppb*, and *Myh7* (**Revised Supplementary Figure 4a**). Importantly, this effect was not attributable to cytotoxicity, as cell viability assessed by CCK-8 assay showed no significant change across the valine concentrations tested (**Revised Supplementary Figure 4b**). Mechanistically, the pro-hypertrophic effect of valine was accompanied by increased levels of the H3K9ibu and H3K23ibu in NRCMs (**Revised Supplementary Figure 4c-e**). Finally, to directly link valine metabolism to the production of the epigenetic contributor isobutyryl-CoA, we measured its intracellular levels. Given the large number of cells required for mass spectrometry analysis, we compared the two most representative conditions: the valine-free (0 mM) control and the highest concentration (10 mM) that elicited the strongest hypertrophic response. Consistent with our hypothesis, the 10 mM valine treatment led to a significant elevation in intracellular isobutyryl-CoA levels (**Revised Supplementary Figure 4f**).

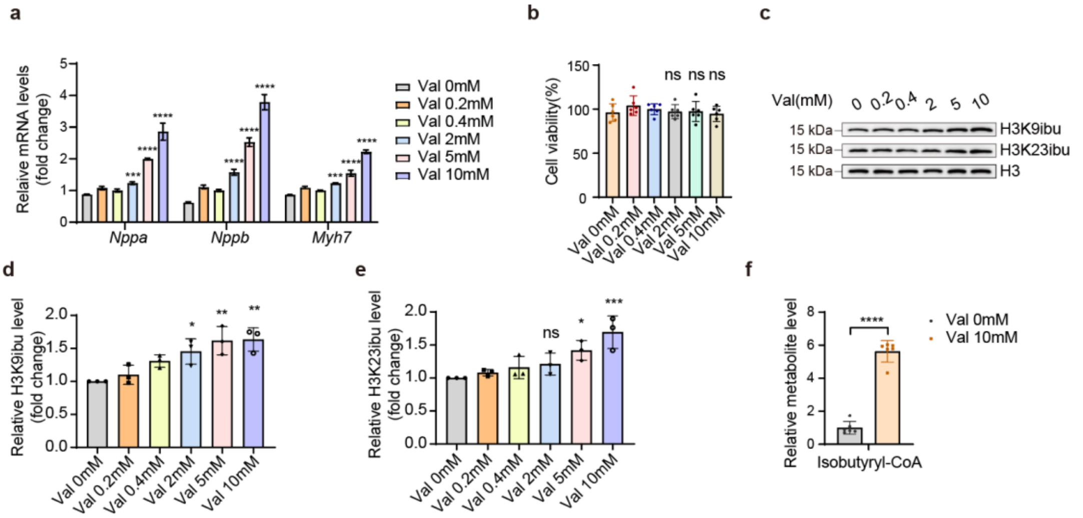

Revised Supplementary Figure 4. a, NRCMs were treated with media containing different concentrations of valine for 36 hours. qRT-PCR was performed to analyse the mRNA levels of hypertrophic genes (n=3; ****p*<0.001, *****p*<0.0001 *vs.* 0 mM). **b,** CCK-8 assay was performed on NRCMs treated with media containing different concentrations of valine for 36 hours (n=6; ns, not significant *vs.* 0mM). **c,** Western blotting and **d-e,** Qualification of H3K9ibu and H3K23ibu levels in NRCMs treated with media containing different concentrations of valine for 36 hours (n=3; ns, not significant, **p*<0.05, ***p*<0.01, ****p*<0.001 *vs.* 0 mM). **f,** Quantification of isobutyryl-CoA levels in NRCMs treated with media containing different concentrations of valine for 36 hours (n=6; *****p*<0.0001).

3) *In vivo* valine loading and isobutyryl-CoA accumulation: Given our cellular findings that valine treatment elevates isobutyryl-CoA and promotes hypertrophy in cardiomyocytes, we carefully evaluated whether a similar valine-loading approach could be meaningfully applied in vivo. While we agree that isobutyryl-CoA levels could potentially increase in failing hearts under conditions of valine excess, several key limitations complicate the interpretation of such an experiment: (*i*) *Lack of tissue specificity*: A high-valine diet may not specifically enrich valine in the heart; its systemic effects could be largely mediated by other tissues (e.g., liver), complicating the interpretation of cardiac-specific outcomes. This is supported by recent evidence showing that a high-BCAA diet increases circulating BCAA levels without affecting their concentration in cardiac tissue[3]. The cardiac functional effects of elevated dietary BCAAs are likely mediated indirectly through changes in plasma BCAA concentration rather than via direct action on cardiomyocyte metabolism[4]. (*ii*) *Confounding signaling effects*: Valine itself can act as a direct signaling molecule and influence cellular processes, complicating the interpretation of in vivo phenotypes. For example, valine has been shown to specifically bind to the SE14 domain of HDAC6 and regulate DNA damage responses[5]. Our in vitro experiments provide more direct evidence that valine elevates isobutyryl-CoA levels, promotes isobutyrylation, and induces cardiomyocyte hypertrophy. (*iii*) *Focus on a pathologically-relevant mechanism*: This study aims to elucidate the role of ACAD8—the key enzyme in cardiac valine catabolism—in pathological hypertrophy. By directly modulating ACAD8, we specifically target the cardiac metabolic flux of isobutyryl-CoA generation, offering a more precise and relevant model to define its pathological role.

In summary, the point you raised is crucial to the main findings and conclusions of this work. Your suggestion has highlighted potential limitations in our current study. Moreover, it provides

a valuable direction for further exploration of key questions in the field of BCAA metabolism and cardiac homeostasis. To provide some in-depth insight, we added the discussion section (page 13 line 417).

"*These facts suggest that ACAD8 deficiency may contribute to cardiac pathology through mechanisms beyond epigenetic reprogramming. While our study provides strong evidence linking isobutyryl-CoA accumulation to histone isobutyrylation and transcriptional activation, the current data do not exclude other contributing mechanisms. For instance, similar to other acyl CoA species, isobutyryl-CoA could directly modify non histone proteins, such as metabolic enzymes or contractile components, thereby influencing cardiac function through alternative pathways[53,54].*"

Comment #2: The data supporting the contribution of downstream histone modification as the underlying mechanism is still largely correlative. Authors indeed noted numerous changes at morphological, sub-cellular levels in addition to transcriptomic changes. While they are correlated with lower isobutyrul-CoA levels in both tissue and cells, the Cut-Tag assays were not performed in heart tissue and were not properly controlled for house-keeping genes. It is also not clear if the c-region of the Nppa gene promoter enriched for H3 Kibu was also colocalized with TEAD2 binding activities based on the Cut-Tag assay.

Response: Thank you for your comments and suggestions. We have carefully addressed each concern as follows.

1) CUT&Tag assay in heart tissues. We sincerely thank the reviewer for this insightful and constructive comment. Following your suggestion, we have consulted the relevant literature, which supports our methodological approach based on the following two key considerations: First, the heart is a complex tissue composed of diverse cell types, each with distinct epigenetic landscapes[6]. Performing CUT&Tag directly on whole tissue would introduce confounding signals from non-cardiomyocytes, thereby obscuring the cell-specific effects we aim to study. Second, standard CUT&Tag protocols are fundamentally optimized and validated for use in defined cellular systems, such as isolated nuclei or single-cell suspensions, to ensure high signal-to-noise ratio and specificity[7]. Moreover, the tissue remaining from our experimental cohorts is insufficient to conduct CUT&Tag assays with the required sample input.

To solve this problem and obtain unambiguous insights, we performed all CUT&Tag validations in NRCMs, which effectively isolates cardiomyocyte-specific epigenetic events. Our results clearly demonstrate that both isobutyrate treatment and *Acad8* knockdown promoted the enrichment of H3K9ibu and H3K23ibu at the promoter region of the hypertrophy-related gene *Nppa*, at least in the context of primary cardiomyocytes. These findings partially support our central mechanism that ACAD8 deficiency promotes cardiac hypertrophy through histone H3 isobutyrylation.

We fully acknowledge that validation in intact cardiac tissue would further strengthen the physiological relevance of our findings. Your suggestion reminds us of the potential limitations of the present work. To provide some in-depth insight, we added the discussion section (page 12 line 392).

"Future studies should further elucidate the regulatory mechanisms of histone isobutyrylation on chromatin remodelling and gene expression, including direct profiling of histone isobutyrylation in cardiac tissue to further validate their functional roles in vivo."

2) Normalization with house-keeping genes. According to your suggestion about internal control, we have re-analyzed the CUT&Tag-qPCR data using the IgG group for quality control and normalized to the *Actb* promoter region as an internal reference (**Revised Supplementary Figure 7a**). The results confirmed that the enrichment trends of H3K9ibu and H3K23ibu at the *Nppa* promoter remained consistent after normalization, while *Actb* itself did not exhibit significant changes across experimental groups (**Revised Figure 4n-o and Revised Supplementary Figure 7b-c**). These key facts support our previous conclusions.

a

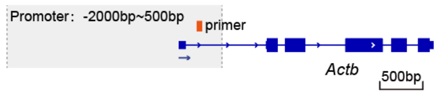

Revised Supplementary Figure 7. a, Schematic showing the *Actb* gene locus, showing the qPCR primer-binding region (238 bp–318 bp) used as an internal reference.

n o

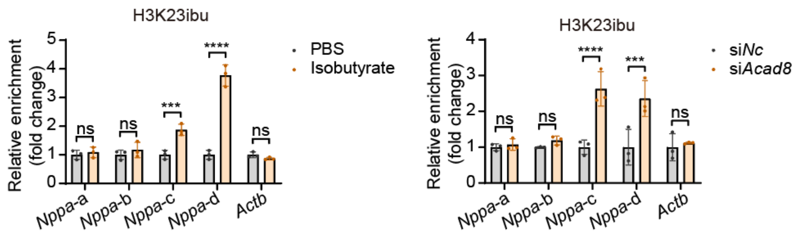

Revised Figure 4. n, NRCMs were treated with PBS or isobutyrate for 48 hours. Subsequently, CUT&Tag assays were performed using antibodies against histone H3K23ibu, followed by qRT-PCR experiments (n=3; ns, not significant, ****p*<0.001, *****p*<0.0001). **o,** NRCMs were transfected with si*Nc* or si*Acad8* for 48 hours. Subsequently, CUT&Tag assays were performed using antibodies against histone H3K23ibu, followed by qRT-PCR experiments (n=3; ns, not significant, ****p*<0.001, *****p*<0.0001).

b c

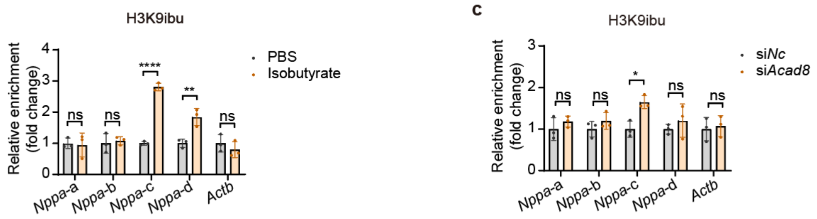

Revised Supplementary Figure 7. b, NRCMs were treated with PBS or isobutyrate for 48 hours. Subsequently, CUT&Tag assays were performed using antibodies against histone H3K9ibu, followed by qRT-PCR experiments (n=3; ns, not significant, ***p*<0.01, *****p*<0.0001). **c,** NRCMs were transfected with si*Nc* or si*Acad8* for 48 hours. Subsequently, CUT&Tag assays were performed using antibodies against histone H3K9ibu, followed by qRT-PCR experiments (n=3; ns, not significant, **p*<0.05).

3) H3Kibu enrichment colocalizes with TEAD2 binding activities. According to your suggestion about colocalization of H3Kibu with TEAD2 binding sites, we performed colocalization analysis between H3Kibu enrichment and TEAD2 binding at the *Nppa* promoter.

In our previous analyses (**Revised Figures 4f–g**), we identified that the *Nppa* promoter d-region spanning –315 bp to –221 bp contains canonical TEAD-binding motifs, as validated by CUT&Tag and qRT-PCR. Using the same primer set targeting this region, we observed that both isobutyrate treatment and *Acad8* knockdown markedly enhanced the enrichment of H3K23ibu in the identical region (**Revised Figures 4n-o**). Within the same genomic region, we observed an overlap between elevated H3K23ibu deposition and a validated TEAD2 binding site. This spatial colocalization suggests that isobutyryl-CoA-dependent histone modification could directly facilitate TEAD2 occupancy at the *Nppa* promoter.

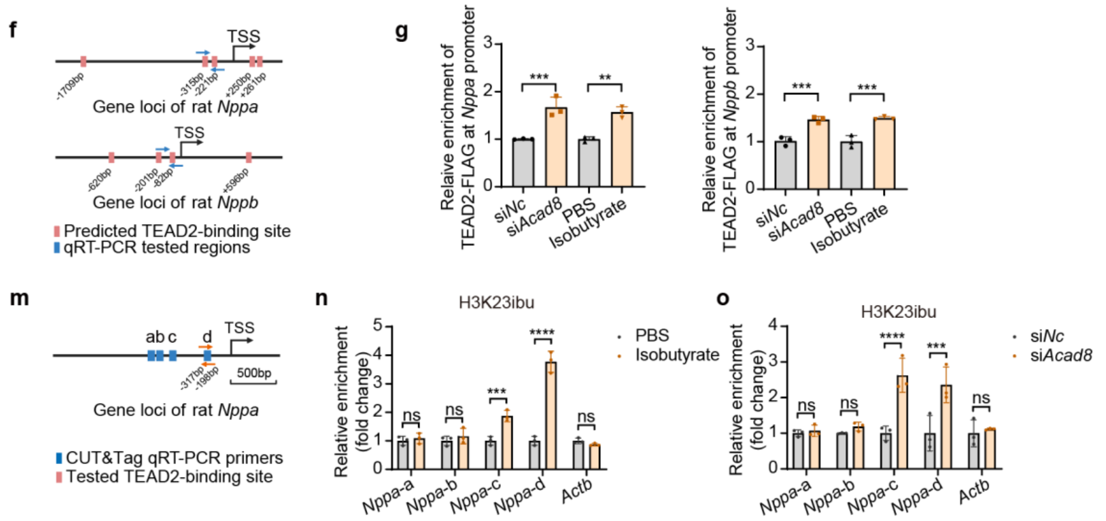

Revised Figure 4. f, Schematic showing the predicted binding sites and validation regions for TEAD2 in the *Nppa* and *Nppb* promoter regions. The exact positions relative to the TSS are labelled below. **g,** NRCMs were transfected with Ad*TEAD2*, then treated with isobutyrate or transfected with si*Nc* or si*Acad8*, followed by CUT&Tag and qRT-PCR experiments (n=3; **p<0.01, ***p<0.001). **m,** Schematic showing the validation sites for isobutyrylation levels at the *Nppa* promoter region. *Nppa*-a region: -882bp~-773bp; *Nppa*-b region: -838bp~-717bp; *Nppa*-c region: -735bp~-608bp; *Nppa*-d region: -317bp~-198bp. **n,** NRCMs were treated with PBS or isobutyrate for 48 hours. Subsequently, CUT-Tag assays were performed using antibodies against histone H3K23ibu, followed by qRT-PCR experiments (n=3; ns, not significant, ***p<0.001, ****p<0.0001). **o,** NRCMs were transfected with si*Nc* or si*Acad8* for 48 hours. Subsequently, CUT&Tag assays were performed using antibodies against histone H3K23ibu, followed by qRT-PCR experiments (n=3; ns, not significant, ***p<0.001, ****p<0.0001).

Comment #3: In addition to adding Isobutyrate (which can be quite non-specific), authors can also test the hypothesis at least in vitro to add or reduce Valine level in the media, and to determine the direct impact on hypertrophic status, isobutyral-CoA level, histone modification or hypertrophic gene expression.

Response: Thank you for your suggestion. Following your advice, we have now performed the proposed experiments to assess the direct effects of modulating extracellular valine concentration on cardiomyocyte. NRCMs were cultured in a valine-depleted medium and then treated with increasing concentrations of valine. The results showed that NRCMs in media supplemented with increasing concentrations of valine resulted in a dose-dependent upregulation of key hypertrophy-related genes, including *Nppa*, *Nppb*, and *Myh7* (**Revised Supplementary Figure 4a**). Importantly, this effect was not attributable to cytotoxicity, as cell viability assessed by CCK-8 assay showed no significant change across the valine

concentrations tested (**Revised Supplementary Figure 4b**). Mechanistically, the pro-hypertrophic effect of valine was accompanied by an increase in histone H3K9ibu and H3K23ibu in NRCMs (**Revised Supplementary Figure 4c-e**). Finally, to directly link valine metabolism to the production of the epigenetic contributor isobutyryl-CoA, we measured its intracellular levels. Given the large number of cells required for mass spectrometry analysis, we compared the two most representative conditions: the valine-free (0 mM) control and the highest concentration (10 mM) that elicited the strongest hypertrophic response. Consistent with our hypothesis, the 10 mM valine treatment led to a significant elevation in intracellular isobutyryl-CoA levels (**Revised Supplementary Figure 4f**).

These results reinforce the role of extracellular valine availability in promoting cardiomyocyte hypertrophy, enhance the specificity of our model linking valine metabolism to histone isobutyrylation, and address the concern regarding the non-specific effects of isobutyrate treatment.

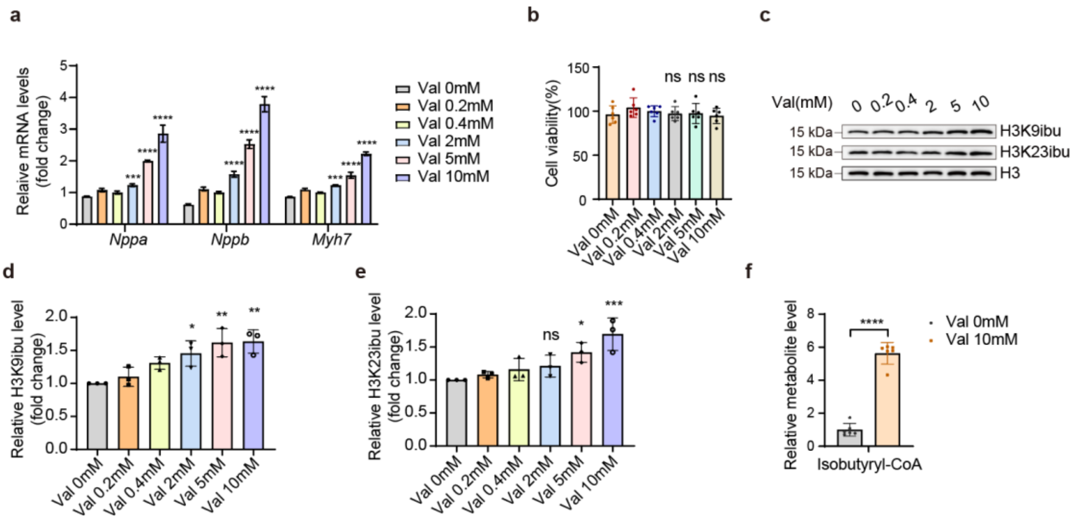

Revised Supplementary Figure 4. a, NRCMs were treated with media containing different concentrations of valine for 36 hours. qRT-PCR was performed to analyse the mRNA levels of hypertrophic genes (n=3; ns, not significant, ***p<0.001, ****p<0.0001 *vs.* 0 mM). **b,** CCK-8 assay was performed on NRCMs treated with media containing different concentrations of valine for 36 hours (n=6; ns, not significant *vs.* 0mM). **c,** Western blotting and **d-e,** Qualification of H3K9ibu and H3K23ibu levels in NRCMs treated with media containing different concentrations of valine for 36 hours (n=3; ns, not significant, *p<0.05, **p<0.01, ***p<0.001 *vs.* 0 mM). **f,** Quantification of isobutyryl-CoA levels in NRCMs treated with media containing different concentrations of valine for 36 hours (n=6; ****p<0.0001 *vs.* 0 mM).

References

1 Yu, H. *et al.* Global crotonylome reveals CDYL-regulated RPA1 crotonylation in homologous recombination-mediated DNA repair. *Science advances* **6**, eaay4697, doi:10.1126/sciadv.aay4697 (2020).

2 Xu, W. *et al.* Global profiling of crotonylation on non-histone proteins. *Cell research* **27**, 946-949, doi:10.1038/cr.2017.60 (2017).

3 Nguyen, D. C. *et al.* Dietary Branched-Chain Amino Acids Modify Postinfarct Cardiac Remodeling and Function in the Murine Heart. *Journal of the American Heart Association* **14**,

e037637, doi:10.1161/jaha.124.037637 (2025).

4 Murashige, D. *et al.* Extra-cardiac BCAA catabolism lowers blood pressure and protects from heart failure. *Cell metabolism* **34**, 1749-1764.e1747, doi:10.1016/j.cmet.2022.09.008 (2022).

5 Jin, J. *et al.* Human HDAC6 senses valine abundancy to regulate DNA damage. *Nature* **637**, 215-223, doi:10.1038/s41586-024-08248-5 (2025).

6 van Weerd, J. H., Koshiba-Takeuchi, K., Kwon, C. & Takeuchi, J. K. Epigenetic factors and cardiac development. *Cardiovascular research* **91**, 203-211, doi:10.1093/cvr/cvr138 (2011).

7 Kaya-Okur, H. S. *et al.* CUT&Tag for efficient epigenomic profiling of small samples and single cells. *Nature communications* **10**, 1930, doi:10.1038/s41467-019-09982-5 (2019).
